# Evaluating re-identification risks scores in publicly available clinical trial datasets: Insights and implications

**DOI:** 10.1177/17407745251356423

**Published:** 2025-08-22

**Authors:** Aryelly Rodriguez, Linda J Williams, Stephanie C Lewis, Pamela Sinclair, Sandra Eldridge, Tracy Jackson, Christopher J Weir

**Affiliations:** 1 Edinburgh Clinical Trials Unit (ECTU), Usher Institute, The University of Edinburgh, Edinburgh, UK; 2 Pragmatic Clinical Trials Unit, Blizard Institute, Barts and the London School of Medicine and Dentistry, Queen Mary University of London, London, UK; 3 Asthma UK Centre for Applied Research, Usher Institute, The University of Edinburgh, Edinburgh, UK

**Keywords:** Clinical trials, data anonymisation, re-identification, de-identification, datasets, risk scores, data sharing

## Abstract

**Background:**

The motivations to share anonymised datasets from clinical trials within the scientific community are increasing. Many anonymised datasets are now publicly available for secondary research. However, it is uncertain whether they pose a privacy risk to the involved participants.

**Methods:**

We located a broad sample of publicly available, de-identified/anonymised randomised clinical trial datasets from human participants and contacted their owners to request access, following their local procedures. We classified personal data within these datasets, including unique direct identifiers such as date of birth and other personal data that, on their own, does not identify an individual but may do so when combined with each other, such as sex, age and race (indirect identifiers). Combining indirect identifiers forms strata, and adding more identifiers increases granularity by dividing the data into a larger number of smaller strata. The re-identification risk score equations evaluate membership in these strata in three ways: first, by measuring the proportions of participants in strata above predetermined risk threshold levels (Ra); second, by locating the smallest stratum (Rb); third, by estimating the average membership across all strata in a dataset (Rc). The risk scores range from 0 (lowest risk) to 1 (highest risk); they do not aim to re-identify individuals in the datasets and are used for routinely collected health records. If a dataset contained a direct identifier, it automatically scored 1 in all metrics. Conversely, if a dataset contained no direct or up to one indirect identifier, it automatically scored 0 in all metrics. Finally, we explored which characteristics of the datasets were associated with the risk scores and compared the risk scores and their usability.

**Results:**

Seventy datasets from 14 data sources were analysed. Thirty-one datasets were shared with minimal restrictions (open access), while 39 were shared with varying levels of restrictions before access was granted (controlled access). Datasets had, on average, four identifiers and mean risk scores ranging from 0.47 to 0.91. The most common pieces of information present in the datasets that, when combined, may indirectly identify a participant were sex (80%) and age (72.9%).

**Conclusions:**

This study confirms that clinical trial datasets are rich in personal details and that using re-identification risk scores as a measure of this richness is feasible. These scores could inform the anonymisation process of clinical trials datasets regarding their level of granularity prior to releasing them for secondary research. We propose a strategy for employing these scores in the decision-making process for releasing clinical trials datasets.

## Background

There is a strong drive, particularly from publishers and funders, to encourage the release of anonymised trial datasets.^1^ New grant applications with funding from the National Institutes of Health,^2^ Cancer Research UK^3^ and the UK Medical Research Council^4^ must contain a concrete data-sharing plan. The International Committee of Medical Journal Editors (ICMJE) requires all clinical trials enrolling participants on or after January 1, 2019, to have a data-sharing plan in their registration.^5^ Also, the ICMJE encourages editors to prioritise publishing work from authors who have shared their data.^6^ Data sharing has become essential in clinical trials for disseminating research, enhancing knowledge through meta-analysis, enabling new investigations on existing datasets and maximising the efforts invested in data collection.^7,8^

Many anonymised datasets are currently available for secondary research via clinical data repositories^9^ or directly from researchers, using either open or controlled access models. Controlled access requires some form of approval before obtaining datasets, whereas open access imposes minimal restrictions.^10^ Anonymisation of data is complex,^10^ and complete anonymisation could result in a loss of detail necessary for appropriate analysis. Therefore, balancing the reduction of re-identification risk while retaining sufficient detail for valid research is crucial. The lack of a gold-standard anonymisation method within the clinical trial community^11^ along with limited resources and training,^12^ complicates this further, leading to uncertainty about the privacy risks posed by available datasets^13,14^ to the involved participants.

We aimed to evaluate some publicly available datasets by calculating their re-identification risk scores using El Emam’s methods.^15^ These scores numerically estimate the potential of re-identifying individuals from anonymised data, helping to assess the effectiveness of anonymisation techniques in preserving privacy. Commonly used for routinely collected health records,^16,17^ these scores theoretically indicate higher re-identification risks with higher values, without aiming to re-identify individuals. We calculated and described these scores, investigated dataset characteristics associated with increased or decreased scores and compared the scores to assess their usability. To our knowledge, no studies have directly used the proposed methods of calculating re-identification risk scores across various publicly available clinical trial datasets.

## Methods

A full protocol (Appendix 1 in the supplementary materials)^18^ was finalised on 1 December 2020.

We collected a sample of publicly available, de-identified/anonymised clinical trials datasets to estimate their re-identification risk scores, using three equations designed for this purpose.^19^

### Datasets sources

We identified 18 data sources through previous research,^18^ web searches and word of mouth. These included 16 repositories and two journals with established data-sharing policies (BMJ and PLOS One). Datasets were requested from repositories between 7 May 2021, and 23 September 2022. We also searched for randomised controlled trials (RCTs) published in the two journals from January 2013 (BMJ) and March 2014 (PLOS One) (when their data-sharing policies were introduced), up until 30 April 2022 (details in Supplementary Appendix 2).

### Types of datasets

Our inclusion criteria consisted of datasets from RCTs on human participants, deemed anonymised and/or de-identified by data holders and suitable for secondary research. We excluded datasets described as containing identifiable information protected solely by controlled access or data-sharing arrangements. We limited our selection to studies with materials available in English or Spanish due to the language skills within the writing team.

### Data collection and analysis

#### Selection and request of datasets

One investigator (AR) searched the sources (repositories or journals) and screened titles and descriptions of datasets to determine eligibility and identify duplicates. For sources with five or fewer eligible datasets, all datasets were requested. For sources with more than five eligible datasets, five were selected at random using SAS^20^ by assigning a random number to each dataset, ordering them based on these random numbers and requesting the first five. If a dataset was unavailable, it was replaced with the next on the ordered list. This exploratory sample ensured a fair representation from all sources and maximised the information we could obtain given our limited resources.

Due to the following factors, we revised this sampling strategy for some sources:

- Project Data Sphere^21^: has two levels of controlled access (researcher vetoing and researcher vetoing plus data-sharing agreements (DSAs)). Initially, five datasets were requested. The signed DSA covered four datasets, while one did not require a DSA. Additional datasets were accessible without a DSA, so we randomly chose another four, totalling nine datasets.
- Large Repositories: Requesting more than five datasets was more efficient, as some owners were unwilling to share certain datasets or the datasets were listed but not yet prepared. Oversampling helped us achieve our target of five datasets and mitigated the inflexibility of DSAs tailored to specific datasets. For instance, at ``The Yale University Open Data Access (YODA) Project’’,^22^ we signed a DSA for five datasets, but one study only contained the clinical study report without the individual participant data. Replacing this dataset would have triggered a new DSA, so YODA is represented by only four datasets.
- BMJ and PLOS One: Studies offering controlled access via DSAs were not pursued due to bottlenecks in our contracts department, and we had already acquired 39 controlled access datasets.

Once a dataset was identified as potentially meeting the inclusion criteria, it was downloaded (if open access), or access was applied for following the data owners’ procedures. The time from request to data access approval was recorded. Some datasets could only be analysed remotely in trusted research environments (TREs).

#### Data extraction and management

The selected datasets were retrieved and transferred to a secure and password-protected electronic storage area at the University of Edinburgh.^23–25^ For datasets held in TREs, AR transferred the re-identification risk scores calculation analysis code to those environments and extracted the relevant output.

Datasets were provided in multiple software formats and thoroughly explored to ensure no discrepancies among formats and to check for additional data. Whenever possible, the datasets were compared with their corresponding data dictionary, case report form and/or protocol to verify if they covered all collected data or were partial datasets.

All available metadata, from obtained datasets, was recorded on an MS Excel^26^ spreadsheet (attributes collected in Supplementary Appendix 3).

#### Data synthesis and re-identification risk calculation

We extracted the number of direct and/or indirect identifiers in the datasets as described by Hrynaszkiewicz et al.,^27^ except for ‘small denominators-population size of <100’ and ‘very small numerators-event counts of <3’ which were already considered within the risk score calculations. The data extraction process involved visual inspection by AR and verification by a second reviewer (SCL, LJW or CJW).

El Emam’s methodology^15^ combines all indirect identifiers to create strata within a dataset. For example, combining age, race and sex generate groups like ‘four 18-year-old white males’ or ‘one 79-year-old African American female’. Adding more identifiers further divides data into smaller strata, known as granularity. The goal is to minimise the number of strata while maintaining data utility. Three re-identification risk scores were calculated^15^:

1. Risk a (Ra): Measures the proportion of participants in a dataset who belong to strata with a re-identification probability higher than seven predefined thresholds (0.01, 0.05, 0.1, 0.2, 0.3, 0.4 and 0.5)^15^ (These thresholds were chosen to represent a wide variety of risks). For instance, an Ra of 0.5 at threshold 0.1 indicates that 50% of the participants in the dataset are in strata with 10 or fewer participants.
2. Risk b (Rb): Identifies the stratum with the smallest membership (regarding all indirect identifiers), representing the worst-case scenario. For example, an Rb of 0.33 indicates that there is at least one stratum with 1 in 3 chances of being re-identified.
3. Risk c (Rc): Represents the average risk score across the whole strata of the dataset, using all indirect identifiers. A dataset with two strata of 5 and 10 participants would have an Rc of 0.15, calculated as the average of 1/5 and 1/10.

Each risk score ranges from 0 to 1 and was estimated under the prosecutor and journalist scenario.^19,28^ The prosecutor re-identification risk arises when an adversary knows that a target individual (whose identifiers are known) is in the publicly available dataset. For this scenario, we assessed uniqueness within strata in each dataset. The journalist re-identification risk occurs when an adversary attempts to re-identify any individual in the dataset by matching it with another dataset, solely to prove that re-identification is possible. In this scenario, we used synthetic datasets, scaled to at least 15 times the size of the anonymised datasets, as identification sources for matching. The synthetic datasets were customised to include corresponding indirect identifiers using the algorithm by Bogle and Erickson.^29^ Supplementary Appendix 4 shows a worked example for calculating Ra, Rb and Rc.

Several assumptions guided the risk score calculations in both scenarios:

- Calculations required at least two indirect identifiers; datasets with fewer were automatically scored 0^30^ for all re-identification risks.
- Datasets containing at least one direct identifier were automatically scored 1^30^ for all re-identification risks.
- No recoding or further manipulation of datasets was allowed, except for necessary steps to prepare data for re-identification risks calculation.

Metadata from all included datasets and re-identification risk scores were summarised using descriptive statistics. There were no attempts to re-identify or contact individual participants. This was an exploratory study, so no formal statistical inference was set a priori.

SAS^20^ was used for all analysis, except for YODA’s^22^ TRE datasets, where STATA^31^ was used due to SAS unavailability.

## Results

The first dataset was received on the 7 May 2021, and the last on the 26 April 2023. Of the 18 identified data sources, three were excluded: two did not contain relevant RCTs datasets, and one no longer existed when the study protocol was executed. Consequently, 15 data sources were visited, 14,896 datasets preselected and a sample of 86 datasets requested. All data sources offered the data free of charge. The median number of requested datasets per data source was 6 (interquartile range [IQR]: 5–6). We obtained 76 out of 86 (88.4%) requested datasets, faced 9 (10.5%) rejections and received one (1.2%) duplicate. Rejections were due to: five times access was denied as our proposal was not considered a valid reason for data sharing, three datasets were listed but not yet available in their repository and one request received no response from the data owners. The median number of obtained datasets per repository was five (IQR: 5–6). (Supplementary Appendix 5).

We analysed 70 out of 76 (92.1%) datasets (representing 14 data sources) and excluded six datasets (7.9%) because four were not from RCTs, and two were summarised cluster data, instead of individual participant data (flowchart in Supplementary Appendix 6). Supplementary Appendix 5 provides a list of the 70 included studies.

### Included datasets’ characteristics

Table 1 summarises the characteristics of the included datasets. Of the 70 datasets, 39 (55.7%) were shared with varying levels of controlled access, while 31 (44.3%) were provided with minimal restrictions (open access). On average, it took 270 days to obtain controlled access datasets and 8 days for open-access datasets. Controlled access sources tended to provide entire datasets (79.5%, 31/39), whereas open access more often supplied only main analysis variables (61.3%, 19/31). This is reflected in the median number of explored variables by type of access: 449(IQR: 217–918) for controlled access vs 92 (IQR: 39–217) for open access. Datasets were provided in multiple software formats (22.9%, 16/70), followed by SAS (21.4%, 15/70) and comma separated values (CSV) (17.1%, 12/70). The most common associated documentation^10,32^ was the study protocol (78.6%, 55/70) and data dictionary (75.7%, 53/70), with 48.6% (34/70) including additional documentation such as patient information sheets, consent forms and supplementary tables.

**Table 1.** Datasets’ characteristics.

Parameter	Category/description	Controlled, N = 39	Open, N = 31	Overall, N = 70
Access, n (%)	Controlled-Vetoing	9 (23.1)	—	9 (12.8)
	Controlled-Vetoing + DSA	16 (41.0)	—	16 (22.9)
	Controlled-Vetoing + DSA + TRE	14 (35.9)	—	14 (20.0)
	Open – No restrictions	—	31 (100)	31 (44.3)
Days to obtain	mean (sd)	270 (125)	8 (43)	154 (163)
	median (IQR)	238 (231–343)	0 (0–0)	138 (0–238)
Type dataset, n (%)	Main analysis	7 (17.9)	19 (61.3)	26 (37.1)
	Complete trial	31 (79.5)	7 (22.6)	38 (54.3)
	Not enough information	1 (2.6)	5 (16.1)	6 (8.6)
No of variables explored, n (%)	mean (sd)	1610 (4076)	160 (211)	968 (3113)
	median (IQR)	449 (217–918)	92 (39–217)	237 (56–572)
File format, n (%)	CSV	6 (15.4)	6 (19.4)	12 (17.1)
	SAS	13 (33.3)	2 (6.5)	15 (21.4)
	SPSS	—	3 (9.7)	3 (4.3)
	STATA	2 (5.1)	3 (9.7)	5 (7.1)
	TXT	5 (12.8)	—	5 (7.1)
	XLSX	—	10 (32.3)	10 (14.3)
	XPT	4 (10.3)	—	4 (5.7)
	Multiple^1^	9 (23.1)	7 (22.6)	16 (22.9)
Data dictionary, n (%)	Available	35 (89.7)	18 (58.1)	53 (75.7)
Case report form, n (%)	Available	23 (59.0)	7 (22.6)	30 (42.9)
Protocol, n (%)	Available	33 (84.6)	22 (71.0)	55 (78.6)
Statistical analysis plan, n (%)	Available	16 (41.0)	7 (22.6)	23 (32.9)
Clinical study report, n (%)	Available	11 (28.2)	—	11 (15.7)
Anonymisation details,^2^ n (%)	Available	13 (33.3)	—	13 (18.6)
Other documents,^3^ n (%)	Available	17 (43.6)	20 (64.5)	34 (48.6)

Where DSA: data-sharing agreement; sd: standard deviation; IQR: interquartile range; TRE: trusted research environment; N: number of datasets; n: number of observations.

1 Multiple refers to two or more of the above formats were available for a single dataset.

2 Documentation detailing how the anonymisation was executed.

3 Other documents refer to patient information sheets, ethical approval letters, standard operating procedures, editorials, summary tables, lay summaries, supplements, consent forms, evaluation instrument manuals.

### Characteristics of the studies associated to the included datasets

Table 2 presents a summary of the observed characteristics of the studies associated with the included datasets. Clinical trial registrations were found for 92.8% (65/70) of the studies. Most registrations occurred from 2006 to 2015 (54.3%, 38/70), and the studies were primarily published between 2011 and 2020 (70.0%, 49/70). Most studies were multicentred (77.1%, 54/70), and many were multinational (32.9%, 23/70), involved adult participants (80%, 56/70) and utilised a parallel design (82.9%, 58/70). Studies were primarily from clinical trial phase III (38.6%, 27/70) and ‘not applicable’ (35.7%, 25/70). The median number of participants was 355 (IQR: 154–921). We included three studies related to rare diseases (4.3%, 3/70) as defined by Orpha.net.^33^

**Table 2.** Associated studies characteristics.

Parameter	Category/description	Controlled N = 39	Open N = 31	Overall N = 70
Year of registration, n (%)	Pre 2000	3 (7.7)	1 (3.2)	4 (5.7)
	2001–2005	7 (17.9)	3 (9.7)	10 (14.3)
	2006–2010	13 (33.3)	2 (6.5)	15 (21.4)
	2011–2015	13 (33.3)	10 (32.3)	23 (32.9)
	2016–2020	1 (2.6)	12 (38.7)	13 (18.6)
	Not required^1^	1 (2.6)	3 (9.7)	4 (5.7)
	Not found/available	1 (2.6)	—	1 (1.4)
Year published, n (%)	Pre 2000	2 (5.1)	1 (3.2)	3 (4.3)
	2001–2005	2 (5.1)	1 (3.2)	3 (4.3)
	2006–2010	—	1 (3.2)	4 (5.7)
	2011–2015	12 (30.8)	4 (12.9)	16 (22.9)
	2016–2020	16 (41.0)	17 (54.8)	33 (47.1)
	2021 and after	3 (7.7)	7 (22.6)	10 (14.3)
	Not published	3 (xx)	—	1 (1.4)
Sites, n (%)	Multicentre	37 (94.9)	17 (54.8)	54 (77.1)
	Single	1 (2.6)	14 (45.2)	15 (21.4)
	Not available	1 (2.6)	—	1 (1.4)
Location, n (%)	Africa	—	3 (9.7)	3 (4.3)
	Asia	3 (7.7)	10 (32.3)	13 (18.6)
	Europe and UK	3 (7.7)	7 (22.6)	8 (14.3)
	Multinational	18 (46.2)	5 (16.1)	23 (32.9)
	Oceania	1 (2.6)	3 (9.7)	4 (5.7)
	USA and Americas	14 (35.9)	3 (9.7)	19 (24.3)
Population, n (%)	Adults	29 (74.4)	27 (87.1)	56 (80.0)
	Adults and children	4 (10.3)	1 (3.2)	5 (7.1)
	Children	6 (15.4)	3 (9.7)	9 (12.9)
Design, n (%)	Cluster	1 (2.6)	4 (12.9)	5 (7.1)
	Crossover	3 (7.7)	1 (3.2)	4 (5.7)
	Factorial	1 (2.6)	1 (3.2)	2 (2.9)
	Parallel	33 (84.6)	25 (80.6)	58 (82.9)
	SMART	1 (2.6)	—	1 (1.4)
Phase, n (%)	I	—	2 (6.5)	2 (2.9)
	II	4 (10.3)	1 (3.2)	5 (7.1)
	III	21 (53.8)	6 (19.4)	27 (38.6)
	IV	2 (5.1)	1 (3.2)	3 (4.3)
	Not applicable	10 (25.6)	15 (48.4)	25 (35.7)
	Not available	2 (5.1)	6 (19.4)	8 (11.4)
Rare disease,^2^ n (%)	Yes	3 (7.7)	—	3 (4.3)
Number of participants	Mean (sd)	1584 (4064)	7952 (29,574)	4404 (19,988)
	Median (IQR)	470 (240–939)	240 (64–903)	355 (154–921)

N: number of datasets; n: number of observations; sd: standard deviation; IQR: interquartile range.

1 Not required refers to studies too old (pre implementation of registration) or not in the remit for registration.

2 As classified by orpha.net.

### Re-identification risk scores results

Table 3 shows that the most common indirect identifiers in the datasets were age (84.3%, 59/70) and sex (80.0%, 56/70), followed by weight (47.1%, 33/70) and height (44.3%, 31/70). Nine (12.9%, 9/70) datasets were automatically risk scored to 0 or 1. Datasets had a median of 4 (IQR: 3–6) identifiers. Mean risk scores ranged from 0.47 to 0.91. Rb was usually higher than Ra (at all thresholds) and Rc. Moreover, the more indirect the identifiers, the higher the risk scores (Supplementary Appendix 6).

**Table 3.** Identifiers and re-identification risks scores for included datasets.

Parameter	Category/description	Controlled N = 39	Open N = 31	Overall N = 70
Indirect identifiers, n (%)^1^	Age	29 (74.4)	22 (70.1)	51 (72.9)
	Age category	7 (17.9)	1 (3.2)	8 (11.4)
	Country	14 (35.9)	4 (12.9)	18 (25.7)
	Education	5 (12.8)	7 (22.6)	12 (17.1)
	Ethnicity	20 (51.3)	4 (12.9)	25 (35.7)
	Race	27 (69.2)	2 (6.5)	28 (40.8)
	Sex	37 (94.9)	21 (67.7)	56 (80.0)
	Marital status	4 (10.3)	1 (3.2)	5 (7.1)
	Occupation	4 (10.3)	1 (3.2)	5 (5.7)
	Height	25 (64.1)	6 (19.4)	31 (44.3)
	Weight	26 (66.7)	7 (22.6)	33 (47.1)
	Date of randomisation	—	3 (9.7)	3 (4.3)
	Others^2^	6	13	19
Number of identifiers, n (%)	0^3^	—	2 (6.5)	2 (2.9)
	1^4^	1 (2.6)	6 (19.4)	7 (10.0)
	2	2 (5.1)	5 (16.1)	7 (10.0)
	3	2 (5.1)	8 (25.8)	10 (14.3)
	4	10 (25.6)	2 (6.5)	12 (17.1)
	5	7 (17.9)	4 (12.9)	11 (15.7)
	6	6 (15.4)	3 (9.7)	9 (12.9)
	7	10 (25.6)	1 (3.2)	11 (15.7)
	8	1 (2.6)	—	1 (1.4)
Number of identifiers	Mean (sd)	5.1 (1.67)	3.0 (1.9)	4.2 (2.06)
	Median (IQR)	5 (4–7)	3 (1–5)	4 (3–6)
Prosecutor Ra, mean (sd)	Threshold at 0.01	0.92 (0.24)	0.86 (0.32)	0.89 (0.28)
	Threshold at 0.05	0.87 (0.31)	0.82 (0.37)	0.85 (0.34)
	Threshold at 0.1	0.84 (0.33)	0.80 (0.38)	0.82 (0.36)
	Threshold at 0.2	0.81 (0.34)	0.76 (0.40)	0.79 (0.37)
	Threshold at 0.3	0.79 (0.35)	0.75 (0.40)	0.77 (0.37)
	Threshold at 0.4	0.76 (0.35)	0.72 (0.41)	0.75 (0.37)
	Threshold at 0.5	0.70 (0.37)	0.65 (0.41)	0.68 (0.39)
Prosecutor Rb, mean (sd)	—	0.93 (0.24)	0.88 (0.33)	0.91 (0.28)
Prosecutor Rc, mean (sd)	—	0.76 (0.34)	0.71 (0.39)	0.74 (0.36)
Journalist Ra, mean (sd)	Threshold at 0.01	0.71 (0.31)	0.81 (0.26)	0.75 (0.29)
	Threshold at 0.05	0.69 (0.30)	0.80 (0.26)	0.74 (0.28)
	Threshold at 0.1	0.60 (0.31)	0.70 (0.31)	0.65 (0.31)
	Threshold at 0.2	0.53 (0.35)	0.61 (0.34)	0.57 (0.34)
	Threshold at 0.3	0.50 (0.35)	0.57 (0.36)	0.53 (0.35)
	Threshold at 0.4	0.48 (0.36)	0.52 (0.37)	0.50 (0.36)
	Threshold at 0.5	0.46 (0.36)	0.48 (0.38)	0.47 (0.37)
Journalist Rb, mean (sd)	—	0.83 (0.35)	0.80 (0.38)	0.82 (0.36)
Journalist Rc, mean (sd)	—	0.73 (0.41)	0.57 (0.43)	0.66 (0.43)

Risk a (Ra): the proportions of participants in strata above a predetermined risk threshold; Risk b (Rb): the stratum with the smallest membership in the anonymised dataset; Risk c (Rc): the average risk score across the whole strata of the anonymised dataset, using all indirect identifiers, calculated with the formulas in chapter 16 from ‘Guide to the de-identification of personal health information’ by Khaled El Emam (2013). N: number of datasets; n: number of observations; sd: standard deviation.

1 These are not mutually exclusive categories; a dataset could have more than one of these indirect identifiers. Therefore, percentages would not add to 100%.

2 Other identifiers refer to, body mass index (BMI), deprivation index, number of children in the family, living siblings, number of pregnancies, education of parents, religion, minority, student status, city, location name, income, socioeconomic status, all with a count of 1, but a dataset could have more than one of these indirect identifiers; therefore, percentage is not applicable.

3 One dataset has no identifiers, and 1 dataset has 1 identifier ‘sex’, both datasets’ risks coded automatically to zero.

4 All with a direct identifier, all datasets’ risks coded automatically to one.

To further explore these results, pre-specified plots in Supplementary Appendix 6 were used. Ra, Rb and Rc did not seem to be correlated with the number of participants. While the risk scores provided distinct aspects of the dataset’s granularity, a correlation between Ra and Rc was noticed; as the threshold increased, the correlation became stronger (Supplementary Appendix 6). Table 4 shows the re-identifications risk scores from Table 3 categorised.

**Table 4.** Re-identification risks scores categorised for included datasets.

		Prosecutor			Journalist		
	Risk interval	Controlled N = 39	Open N = 31	Overall N = 70	Controlled N = 39	Open N = 31	Overall N = 70
Ra at 0.01	0	1 (2.6)	2 (6.5)	3 (4.3)	—	2 (6.5)	2 (2.9)
n (%)	0> and ≤0.25	1 (2.6)	1 (3.2)	2 (2.9)	5 (12.8)	—	5 (7.1)
	0.25> and ≤0.5	2 (5.1)	2 (6.5)	4 (5.7)	3 (7.7)	1 (3.2)	4 (5.7)
	0.5> and ≤0.75	—	—	—	4 (10.3)	5 (16.1)	9 (12.9)
	0.75> and <1	—	1 (3.2)	1 (1.4)	23 (59.0)	14 (45.2)	37 (52.9)
	1	35 (89.7)	25 (80.6)	60 (85.7)	4 (10.3)	9 (29.0)	13 (18.6)
Ra at 0.05	0	1 (2.6)	3 (9.7)	4 (5.7)	—	2 (6.5)	2 (2.9)
n (%)	0> and ≤0.25	4 (10.3)	2 (6.5)	6 (8.6)	5 (12.8)	—	5 (7.1)
	0.25> and ≤0.5	—	1 (3.2)	1 (1.4)	3 (7.7)	1 (3.2)	4 (5.7)
	0.5> and ≤0.75	1 (2.6)	—	1 (1.4)	5 (12.8)	5 (16.1)	10 (14.3)
	0.75> and <1	4 (10.3)	1 (3.2)	5 (7.1)	23 (59.0)	15 (48.4)	38 (54.3)
	1	29 (74.4)	24 (77.4)	53 (75.7)	3 (7.7)	8 (25.8)	11 (15.7)
Ra at 0.1	0	2 (5.1)	3 (9.7)	5 (7.1)	1 (2.6)	3 (9.7)	4 (5.7)
n (%)	0> and ≤0.25	3 (7.7)	2 (6.5)	2 (2.9)	6 (15.4)	1 (3.2)	7 (10.0)
	0.25> and ≤0.5	1 (2.6)	2 (6.5)	3 (4.3)	6 (15.4)	1 (3.2)	7 (10.0)
	0.5> and ≤0.75	1 (2.6)	—	1 (1.4)	6 (15.4)	8 (25.8)	14 (20.0)
	0.75> and <1	5 (12.8)	1 (3.2)	6 (8.6)	18 (46.2)	12 (38.7)	30 (42.9)
	1	27 (69.2)	23 (74.2)	50 (71.4)	2 (5.1)	6 (19.4)	8 (11.4)
Ra at 0.2	0	2 (5.1)	4 (12.9)	6 (8.6)	4 (10.3)	3 (9.7)	7 (10.0)
n (%)	0> and ≤0.25	3 (7.7)	3 (9.7)	6 (8.6)	7 (17.9)	2 (6.5)	9 (12.9)
	0.25> and ≤0.5	2 (5.1)	—	2 (2.9)	4 (10.3)	4 (12.9)	8 (11.4)
	0.5> and ≤0.75	—	2 (6.5)	2 (2.9)	8 (20.5)	10 (32.3)	18 (25.7)
	0.75> and <1	10 (25.6)	6 (19.4)	16 (22.9)	14 (35.9)	6 (19.4)	20 (28.6)
	1	22 (56.4)	16 (51.6)	38 (54.3)	2 (5.1)	6 (19.4)	8 (11.4)
Ra at 0.3	0	3 (7.7)	4 (12.9)	7 (10.0)	4 (10.3)	4 (12.9)	8 (11.4)
n (%)	0> and ≤0.25	3 (7.7)	3 (9.7)	6 (8.6)	8 (20.5)	4 (12.9)	12 (17.1)
	0.25> and ≤0.5	1 (2.6)	—	1 (1.4)	6 (15.4)	2 (6.5)	8 (11.4)
	0.5> and ≤0.75	2 (5.1)	2 (6.5)	4 (5.7)	5 (12.8)	10 (32.3)	15 (21.4)
	0.75> and <1	10 (25.6)	7 (22.6)	17 (24.3)	14 (35.9)	5 (16.1)	19 (27.4)
	1	20 (51.3)	15 (48.4)	35 (50.0)	2 (5.1)	6 (19.4)	8 (11.4)
Ra at 0.4	0	3 (7.7)	4 (12.9)	7 (10.0)	4 (10.3)	5 (16.1)	9 (12.9)
n (%)	0> and ≤0.25	3 (7.7)	3 (9.7)	6 (8.6)	10 (25.6)	4 (12.9)	14 (20.0)
	0.25> and ≤0.5	1 (2.6)	2 (6.5)	3 (4.3)	4 (10.3)	5 (16.1)	9 (12.9)
	0.5> and ≤0.75	6 (15.4)	—	6 (8.6)	5 (12.8)	7 (22.6)	12 (17.1)
	0.75> and <1	6 (15.4)	8 (25.8)	14 (20.0)	14 (35.9)	4 (12.9)	18 (25.7)
	1	20 (51.3)	14 (45.2)	34 (48.6)	2 (5.1)	6 (19.4)	8 (11.4)
Ra at 0.5	0	3 (7.7)	4 (12.9)	7 (10.0)	5 (12.8)	5 (16.1)	10 (14.3)
n (%)	0> and ≤0.25	4 (10.3)	4 (12.9)	8 (11.4)	11 (28.2)	5 (16.1)	16 (22.9)
	0.25> and ≤0.5	5 (12.8)	2 (6.5)	7 (10.0)	3 (7.7)	7 (22.6)	10 (14.3)
	0.5> and ≤0.75	4 (10.3)	3 (9.7)	7 (10.0)	4 (10.3)	4 (12.9)	8 (11.4)
	0.75> and <1	11 (28.2)	5 (16.1)	16 (22.9)	14 (35.9)	4 (12.9)	18 (25.7)
	1	12 (30.8)	13 (41.9)	25 (35.7)	2 (5.1)	6 (19.4)	8 (11.4)
Rb	0	—	2 (6.5)	2 (2.9)	—	2 (6.5)	2 (2.9)
n (%)	0> and ≤0.25	3 (7.7)	2 (6.5)	5 (7.1)	6 (15.4)	4 (12.9)	10 (14.3)
	0.25> and ≤0.5	—	—	—	2 (5.1)	1 (3.2)	3 (4.3)
	0.5> and ≤0.75	—	—	—	—	—	—
	0.75> and <1	—	—	—	—	—	—
	1	36 (92.3)	27 (87.1)	63 (90.0)	31 (79.5)	24 (77.4)	55 (78.6)
Rc	0	—	2 (6.5)	2 (2.9)	—	2 (6.5)	2 (2.9)
n (%)	0> and ≤0.25	6 (15.4)	5 (16.1)	11 (15.7)	10 (25.6)	8 (25.8)	18 (25.7)
	0.25> and ≤0.5	1 (2.6)	1 (3.2)	2 (2.9)	1 (2.6)	4 (12.9)	5 (7.1)
	0.5> and ≤0.75	7 (17.9)	3 (9.7)	10 (14.3)	1 (2.6)	3 (9.7)	4 (5.7)
	0.75> and <1	13 (33.3)	7 (22.6)	20 (28.6)	2 (5.1)	1 (3.2)	3 (4.3)
	1	12 (30.8)	13 (41.9)	25 (35.7)	25 (64.1)	13 (41.9)	38 (54.3)

Risk a (Ra): the proportions of participants in strata above a predetermined risk threshold; Risk b (Rb): the stratum with the smallest membership in the anonymised dataset; Risk c (Rc): the average risk score across the whole strata of the anonymised dataset, using all indirect identifiers, calculated with the formulas in chapter 16 from ‘Guide to the de-identification of personal health information’ by Khaled El Emam (2013). N: number of datasets; n: number of observations.

We did not encounter any reportable critical issues with the analysed datasets; hence, there was no need for us to communicate with any of the data owners or holders.

### Re-identification risk scores in action

To understand the behaviour of the risk scores, we conducted two exploratory comparisons on three of the acquired 70 datasets. First, we used the TOPPIC^34^ trial anonymised dataset and calculated its risk score according to our protocol. We obtained 240 unique strata, matching its number of participants, and all risk scores were 1. Next, we categorised its continuous indirect identifiers (age, height and weight) in bands of 10 units. This yielded 123 unique strata, with risk scores ranging from 0.27 to 1. We then grouped them into bands of 20 units, resulting in 52 unique strata and risk scores ranging from 0.07 to 1. Then, we collapsed bands with counts less than 5 with their most adjacent band, producing 44 unique levels, with risk scores ranging from 0.05 to 1. From this last categorisation, we also removed age, then reinstated it and subsequently excluded weight. In both cases, the number of strata were 17 and 15 respectively, with risk scores from 0.01 to 1 and 0.004 to 1 (Supplementary Appendix 6).

Second, we compared risk calculations for two datasets (Supplementary Appendix 6). Although both datasets have three indirect identifiers, all risk scores are higher for the IST^35^ trial dataset (19435 participants and 2570 unique strata) compared to the RESTART^36^ trial dataset risk scores (537 participants – 8 unique levels), as the former is more granular than the latter.

## Discussion

### The experience of dataset request and extraction

Securing 76 out of 86 (88.4%) requested datasets from 15 data sources indicates a widespread willingness to share data, which is reassuring. However, our affiliation with a reputable academic institution likely improved our chances of securing them. Remarkably, we were not charged any fees by the data holders, which is encouraging given the evolving and increasingly strict regulatory environment for processing personal data.^37,38^ This situation may change in the future as data holders might start charging for the extra work involved,^30,39^ especially if this activity is not adequately funded. Conversely, well-funded data-sharing initiatives should generate pre-prepared data packs, helping minimise cost.

The time to obtain most open access datasets was short, 30 datasets received in 0 days, while one outlier taking 241 days, which the owner agreed to provide almost immediately, but it took 241 days to locate and send it. In contrast, procuring controlled access dataset, which required DSAs, was a lengthy and arduous process. Multiple forms needed to be completed before reaching the DSA stage, which often involved extensive negotiations between legal departments. This process should be reviewed, as it should not take nearly 9 months to a year to obtain the datasets.

We observed, as have other researchers^40^ before us, that controlled access is not a universal concept but involves a variety of processes. At the simplest end, we only had to fill out a request form, submit our curricula vitae and outline our research question (23.1%, 9/39 datasets). The next level of complexity involved signing DSAs alongside the initial steps (41.0%, 16/39). The most complicated process entailed the additional step of accessing the shared data in a TRE (35.9%, 14/39).

### The characteristics of the data packs

Certain characteristics slowed down our analysis: unclear or unavailable data dictionaries, variables repeated multiple times requiring consistency checks and outdated software formats. Conversely, some datasets following the Clinical Data Interchange Standards Consortium (CDISC) Study Data Tabulation Model^41^ or Analysis Data Model^42^ expedited the analysis. Datasets should be accompanied by clear data dictionaries, avoid duplicated variables and be stored in basic formats like CSV or TXT to avoid compatibility issues, or adhere to recognised standards.

### The interpretation of re-identification risk scores

We found that 10% (7/70) of the datasets were labelled as anonymised, yet they contained direct identifiers (personal details), such as date of birth and participants’ initials. These datasets were included in the analysis to highlight this issue, with all their risk scores automatically set to 1, representing the worst-case scenario. Researchers must carefully cross-check their anonymised datasets with the list provided by Hrynaszkiewicz et al.^27^ before releasing them. Certain dataset characteristics increased the risk scores, for instance, we encountered exact ages (e.g. measured in days) and dates of randomisation, which could be used to reverse-engineer dates of birth. Some datasets included the ‘Date of Death’ for participants; under the General Data Protection Regulation, personal data protection is not applicable once an individual is deceased. However, Hrynaszkiewicz et al.^27^ recommend removing all dates unique to a participant, as this could potentially impact any living relatives. Replacing exact dates with the number of days from randomisation retains analytical value while protecting privacy.

Notably, 2.9% (2/70) of the datasets had no identifiers or only one indirect identifier, resulting in risk scores automatically set to zero. Datasets with no identifiers could be freely shared without privacy implications and are a viable option for researchers. However, even if one (or no) indirect identifier remains, a holistic check must be made. For example, one obtained dataset recorded only age, but its publication indicated that all participants were female and located in a specific region of the UK.

Re-identification risk scores evaluate dataset granularity. As shown in the TOPPIC^34^ example, continuous identifiers increase granularity and risk scores more than discrete ones. Since each re-identification risk score assesses different aspects of dataset granularity, we cannot recommend one over the others. Furthermore, we cannot comment on their absolute magnitude as there are no standards or examples for comparison in clinical trials. However, smaller scores generally indicate better privacy protection.^15^ Regarding Ra, statistical disclosure control^43^ suggests suppressing table cells with counts less than five (i.e. a threshold of 0.2). Releasing datasets at this threshold could be an option, but it might reduce data usability. Researchers need to set a threshold that balances their data utility and privacy risk. More importantly, it is crucial to acknowledge that the threshold cannot ever be zero if indirect identifiers are present. This understanding emphasises that anonymisation is a spectrum, not a binary state, and some risk must be endured.^30^ Rb behaved like a discrete variable, often scoring 1 with few exceptions, regardless the number of identifiers. This outcome was expected since most included datasets had at least one stratum represented by only one participant. In the TOPPIC^34^ trial dataset comparison, Rb stubbornly remained at 1 despite attempts to reduce granularity. Lowering Rb^30^ requires more sophisticated approaches like k-anonymity,^44^ where the smallest membership per stratum should be k. Clinical trials researchers might need support in learning new techniques to address this. Finally, Rc showed that datasets not only had a few problematic strata (i.e. with 1 or 2 participants) but also many strata with low participant membership.

### Using re-identification risk scores

**Figure 1.**
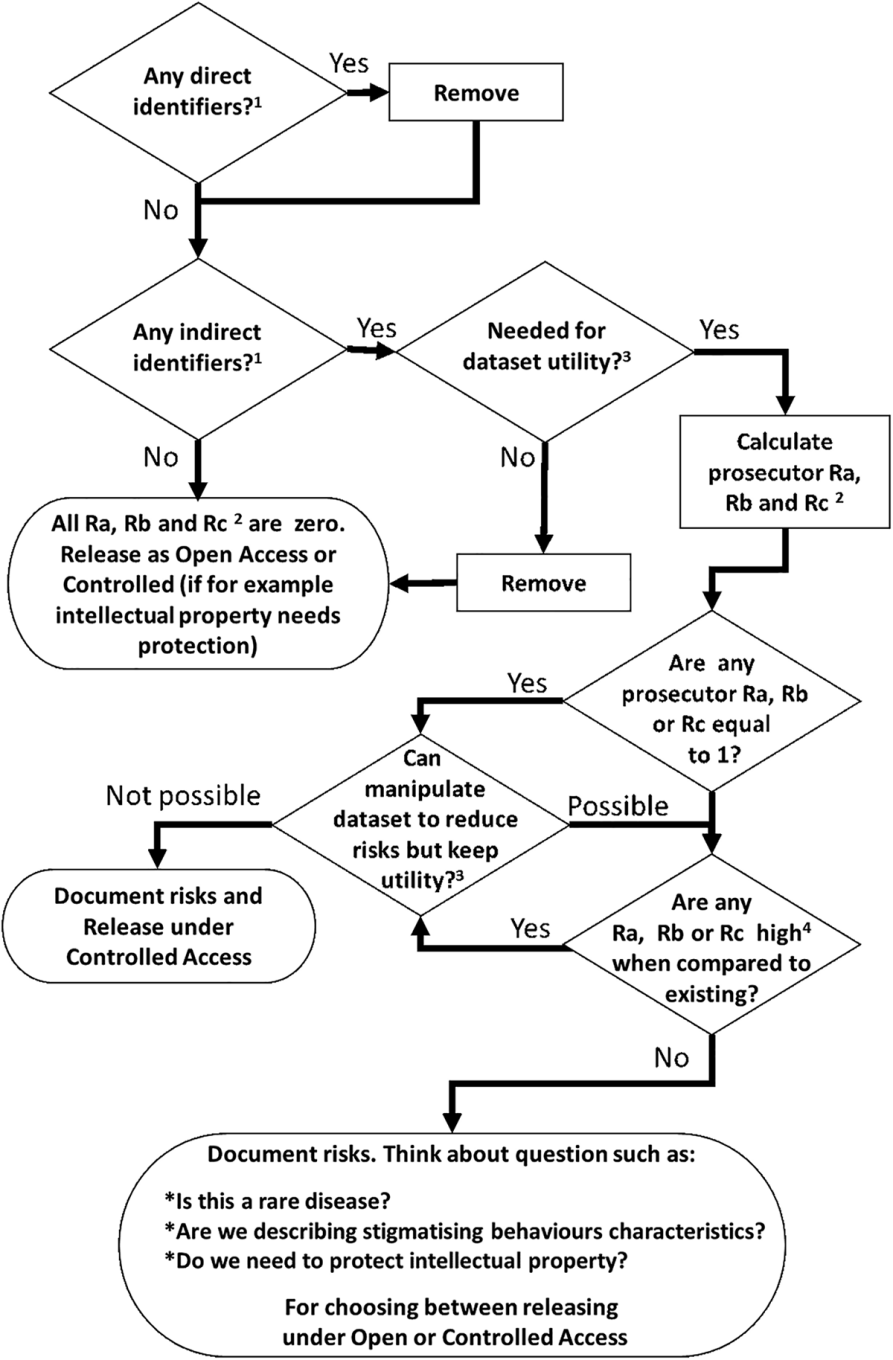
Decision cycle for releasing clinical trials datasets using re-identification risks. ^1^As described by Hrynaszkiewicz et al.^27^ ^2^Risk a (Ra): the proportions of participants in strata above a predetermined risk threshold; Risk b (Rb): the stratum with the smallest membership in the anonymised dataset; Risk c (Rc): the average risk score across the whole strata of the anonymised dataset, using all indirect identifiers, calculated with the formulas in chapter 16 from ‘Guide to the de-identification of personal health information’ by Khaled El Emam (2013). ^3^Utility is interpreted as the ability to reproduce the primary outcome analysis with the manipulated indirect identifiers. ^4^Using the parameters in Figures 2 and 3 of this article.

We recommend the process outlined in Figure 1 (adapted from El Emam)^45^ to effectively use the risk scores. First, remove all direct identifiers, followed by verifying the presence of indirect identifiers. Next, question the necessity of these indirect identifiers to maintain utility.^46^ Note, there is no universally agreed-upon interpretation of data utility, it is context-specific. Researchers must define what utility means for their study. The ability to reproduce the primary outcome analysis with the anonymised dataset can serve as a measure of utility.^40,46^ The researcher’s definition of utility^47^ is pivotal in shaping the anonymised datasets. For example, the TOPPIC^34^ trial dataset was designed to allow investigation of new research questions (423 variables, 240 participants, 240 unique levels with four indirect identifiers, Supplementary Appendix 6) while the RESTART^36^ trial dataset was designed from primary analysis replication (66 variables, 537 participants, eight unique levels with three indirect identifiers, Supplementary Appendix 6). Once utility is defined, calculate the risk scores, if they are considered high, they could be lowered by manipulating the data through perturbation, recalculation, recoding and suppression^11^ and by using privacy models.^44,48–50^ Finally, use Table 4 to compare the newly anonymised dataset’s re-identification risk scores with those from the datasets included in this research and decide the type of access to be used for release.

### Strengths and limitations

This study covers an emerging area in clinical trials methods research, where even the definition of anonymisation lacks consensus. Therefore, our selection of datasets defined as anonymised or de-identified may have introduced heterogeneity among the characteristics of the requested datasets.

The available datasets currently underrepresent Africa and Oceania, which might limit the applicability of our findings to those continents. There were no restrictions on the age of the selected datasets; however, to enhance the representation of open access datasets, we modified the inclusion criteria for datasets from BMJ and PLOS One. This may have skewed our findings and affected the analysis of publication dates and datasets ages, as our search for these sources was limited to datasets from January 2013 and March 2014 onward, respectively.

We did not find, or actively seek, trials studying risky or stigmatising behaviour, which would require special treatment beyond the scope of this research.^51–53^ Furthermore, the journalist re-identification risk scores might have been overestimated, as all the matching theoretical datasets were 15 times larger than the original dataset. This scale might not have been sufficient in some cases, especially for small datasets (fewer than 40 participants). Moreover, the probability of a real-life matching dataset existing was not evaluated, as this requires specialised expertise. Given these challenges, using only the prosecutor risk scenario may have been a more appropriate and feasible initial approach.

At least 19 open-access datasets were available when we wrote the protocol for this research, making the project feasible. However, we could not have predicted that controlled-access repositories would be as receptive to our requests as they were. In hindsight, with greater ambition, we could have requested more datasets, thereby increasing our sample size, but given limited resources, it could not have increased significantly.

The re-identification risk scores alone do not determine a dataset’s real-world vulnerability to re-identification attacks,^54–57^ factors such as an attacker’s motivations, resources and potential gains also play a role, which were outside the scope of this research.

Risk score calculations can be rapidly implemented using the freely available R package sdcMicro^58–60^ or methods outlined in this publication’s appendices. While this research was conducted by experienced clinical trial statisticians, clinical trial researchers with a strong understanding of trial datasets could also perform these calculations. The main strength of this research is its assessment of a variety of datasets using a simple methodology under the same conditions.

## Conclusion

This study confirms a strong inclination to share clinical trial datasets, which are rich in personal details. Although re-identification risk scores may appear oversensitive for clinical trial datasets, which are typically smaller than datasets from electronic health records, their high magnitudes do not necessarily translate into real-life re-identification threats for participants. Instead, these scores provide valuable summaries for understanding the granularity of clinical trial datasets, aiding decision-making for dataset release for secondary purposes.

We have demonstrated that calculating re-identification risk scores is simple and feasible. The number and type of identifiers (continuous or discrete) are crucial in controlling risk scores. More identifiers increase granularity, with continuous identifiers adding more granularity than discrete ones. While risk scores alone cannot determine if data is sufficiently anonymised or protected, they can assist in calibrating the anonymisation process of clinical trial datasets.

Researchers can use our findings to compare their anonymised datasets with those we evaluated and should consider employing the process outlined in this research to guide their anonymisation procedures before data release. This includes providing clear data dictionaries and offering datasets in simple formats. The proposed method is a cost-effective and pragmatic solution. While simple, it is highly informative and serves as a useful stopgap, especially when resources are limited.

**Table:** 

*id*	Source (included studies)	Requested acknowledgments statements
*1*	https://datacompass.lshtm.ac.uk^61^ (NCT02104232,^62^ NCT02111915,^63^ ISRCTN36436933)^64^	No statement required.
*2*	https://ctu-app.lshtm.ac.uk/freebird^65^ (ISRCTN7445979,^66^ NCT00375258,^67^ NCT00872469^68^ NCT00872469,^69^ NCT03777488)^70^	No statement required
*3*	https://datashare.is.ed.ac.uk^71^ (ISRCTN45178534,^72^ ISRCTN25765518,^73^ IST (Registration not required),^35^ ISRCTN71907627^36^ ISRCTN89489788)^34^	We gratefully acknowledge the IST-3 Collaborative Group, the trial joint sponsors (The University of Edinburgh and the Lothian Health Board) and the chief funding agencies of the study: UK Medical Research Council, Health Foundation UK, Stroke Association UK, Research Council of Norway, Arbetsmarknadens Partners Forsakringsbolag (AFA) Insurances Sweden, Swedish Heart Lung Fund, The Foundation of Marianne and Marcus Wallenberg, Polish Ministry of Science and Education, the Australian Heart Foundation, Australian National Health and Medical Research Council (NHMRC), Swiss National Research Foundation, Swiss Heart Foundation, Assessorato alla Sanita, Regione dell’Umbria, Italy, and Danube University
*4*	https://www.clinicalstudydatarequest.com^74^ (Registration not required,^75^ NCT01822899^76^ NCT01842607,^77^ NCT01405053,^78^ NCT00948766)^79^	This publication is based on research using data from the Sponsor companies GlaxoSmithKline Research & Development Ltd, Eisai Limited and Novartis Pharma AG that have been made available to us through secured access. CSDR team or Sponsors have not contributed to or approved, and are not in any way responsible for, the contents of this publication. We thank both Sponsors and CSDR for providing us data and access.
*5*	http://datadryad.org^80^ (ACTRN12616000888460,^81^ HKCTR-1848^82^ Registration no required,^83^ NCT04523831)^84^	No statement required
*6*	http://yoda.yale.edu^22^ (NCT01715285,^85^ NCT00903331^86^ NCT01004432,^87^ NCT00211133)^88^	This study, carried out under YODA Project 2022-4951, used data obtained from the Yale University Open Data Access Project, which has an agreement with JANSSEN RESEARCH & DEVELOPMENT, L.L.C. The interpretation and reporting of research using this data are solely the responsibility of the authors and does not necessarily represent the official views of the Yale University Open Data Access Project or JANSSEN RESEARCH & DEVELOPMENT, L.L.C.
*7*	https://www.projectdatasphere.org^21^(NCT00058474,^89^ NCT00033293,^90^ NCT00310180,^91^ NCT00693992,^92^ NCT00312208,^93^NCT00143455^94^ NCT00113763,^95^NCT00617669,^96^ NCT00676650)^97^	This publication is based on information obtained from https://data.projectdatasphere.org, which is maintained by Project Data Sphere, and includes information that has been made available by the National Cancer Institute and is also available through the National Clinical Trials Network (NCTN)/NCI Community Oncology Research Programme (NCORP) Data Archive. The information was collected from the following clinical trials:
		• A Clinical Trial Comparing Preoperative Radiation Therapy And Capecitabine With or Without Oxaliplatin With Preoperative Radiation Therapy And Continuous Intravenous Infusion Of 5-Fluorouracil With or Without Oxaliplatin In The Treatment Of Patients With Operable Carcinoma Of The Rectum. NCT00058474.
		• A Pilot Study Randomised Trial of Intravenous Gammaglobulin Therapy for Patients With Neuroblastoma Associated Opsoclonus-Myoclonus-Ataxia Syndrome Treated With Chemotherapy and Prednisone. NCT00033293.
		• Programme for the Assessment of Clinical Cancer Tests (PACCT-1): Trial Assigning Individualised Options for Treatment: The TAILORx Trial. NCT00310180.
		• Randomised, Phase III, Double-Blind Placebo-Controlled Trial of Sunitinib (NSC #736511) as Maintenance Therapy in Non-progressing Patients Following an Initial Four Cycles of Platinum-Based Combination Chemotherapy in Advanced, Stage IIIB / IV Non-small Cell Lung Cancer. NCT00693992.
		• A Multicenter Phase III Randomised Trial Comparing Docetaxel in Combination With Doxorubicin and Cyclophosphamide Versus Doxorubicin and Cyclophosphamide Followed by Docetaxel as Adjuvant Treatment of Operable Breast Cancer HER2neu Negative Patients With Positive Axillary Lymph Nodes. NCT00312208.
		• Open Label, Randomised Multicentre Phase III Study Of Irinotecan Hydrochloride (Campto (Registered)) And Cisplatin Versus Etoposide And Cisplatin In Chemotherapy Naive Patients With Extensive Disease–Small Cell Lung Cancer. NCT00143455.
		• An Open-label, Randomised, Phase 3 Clinical Trial of ABX-EGF Plus Best Supportive Care Versus Best Supportive Care in Subjects With Metastatic Colorectal Cancer. NCT00113763.
		• A Phase III, Randomised, Double-blind, Placebo-controlled Study to Assess the Efficacy and Safety of 10 mg ZD4054 (Zibotentan) in Combination With Docetaxel in Comparison With Docetaxel in Patients With Metastatic Hormone-resistant Prostate Cancer. NCT00617669.
		• A Multicenter, Randomised, Double-Blind, Phase 3 Study Of Sunitinib Plus Prednisone Versus Prednisone In Patients With Progressive Metastatic Castration-Resistant Prostate Cancer After Failure Of A Docetaxel-Based Chemotherapy Regimen. NCT00676650.
		All analyses and conclusions in this publication are the sole responsibility of the authors and do not necessarily reflect the opinions of the owners of the information, the clinical trial investigators, the NCTN, the NCORP, the National Cancer Institute, or Project Data Sphere. Neither the owners of the information, the clinical trial investigators, the NCTN, the NCORP, the National Cancer Institute, nor Project Data Sphere have contributed to, approved or are in any way responsible for the contents of this publication
*8*	https://biolincc.nhlbi.nih.gov/studies^98^ (NCT00650091,^99^ NCT00000589,^100^ NCT00075829,^101^ NCT01982968,^102^ NCT01134783,^103^ NCT00004562)^104^	This Manuscript was prepared using BMTCTN0102, CHOICES, PANTHER, OAT, TRAP, WRAP_IPF Research Materials obtained from the NHLBI Biologic Specimen and Data Repository Information Coordinating Centre and does not necessarily reflect the opinions or views of the BMTCTN0102, CHOICES, PANTHER, OAT, TRAP, WRAP_IPF or the NHLBI
*9*	https://nda.nih.gov/get/access-data.html^105^ (NCT00012558,^106^ Registration not found,^107^ NCT01927276,^108^ NCT01944046,^109^ NCT00005013)^110^	Data and/or research tools used in the preparation of this manuscript were obtained from the National Institute of Mental Health (NIMH) Data Archive (NDA). NDA is a collaborative informatics system created by the National Institutes of Health to provide a national resource to support and accelerate research in mental health. Dataset identifier(s): 3058, 2147, 2622, 2724, 2009, 2157. This manuscript reflects the views of the authors and may not reflect the opinions or views of the NIH or of the Submitters submitting original data to NDA.
*10*	https://vivli.org/^111^ (NCT01198756,^112^ NCT01573767,^113^ NCT01313676,^114^ NCT00400855,^115^ NCT01498822)^116^	This publication is based on research using data from data contributors GSK and UCB that has been made available through Vivli, Inc. Vivli has not contributed to or approved, and is not in any way responsible for, the contents of this publication.
*11*	https://beta.ukdataservice.ac.uk/datacatalogue/studies (reshare.ukdataservice.ac.uk) (https://www.ukdataservice.ac.uk/deposit-data) ^117^ (ISRCTN11288961,^118^ ISRCTN90749868^119^ NCT01801410,^120^ Registration not required,^121^ ISRCTN24081411)^122^	To Nottingham Clinical Trials Unit for facilitating sharing of data from the PRIDE study via ukdataservice.ac.uk
*12*	https://med.data.edu.au/find-data/ ^123^	Repository no longer available
*13*	https://dcri.org/our-approach/data-sharing/soar-data^124^ SOAR data: Available datasets: Duke cardiac catheterization datasets.	We acknowledge Michael Cohen-Wolkowiez, who is the principal investigator who conducted the original study from which the data were generated. Furthermore, we acknowledge the Eunice Kennedy Shriver National Institute of Child Health and Human Development Data and Specimen Hub for providing the Pharmacokinetics of Clindamycin and Trimethoprim-sulfamethoxazole in Infants and Children (PBPK) data that were used for this research.
*14*	https://journals.plos.org/plosone/search^125^ (NCT02700490,^126^ TCTR20201005002,^127^ NCT02185196,^128^ACTRN12616000538448,^129^ ISRCTN 71217488^130^ NCT02747524)^131^	No statement required
*15*	https://www.bmj.com/search/advanced^132^ (NCT02068885,^133^, NCT01953549,^134^ ISRCTN11980540)^135^	No statement required
*16*	https://dataverse.harvard.edu/^136^ (CTRI/2016/09/007240,^137^ PACTR201901905832601^138^ NCT02148952,^139^ SLCTR/2019/015,^140^ ANZCTR12616001367437)^141^	No statement required
*17*	https://arlg.org/studies-in-progress/ ^142^	Not applicable as RCT data was not located in this repository
*18*	https://repository.niddk.nih.gov/studies/ ^143^	Not applicable as RCT data was not located in this repository

## Supplemental Material

sj-docx-1-ctj-10.1177_17407745251356423 – Supplemental material for Evaluating re-identification risks scores in publicly available clinical trial datasets: Insights and implications Supplemental material, sj-docx-1-ctj-10.1177_17407745251356423 for Evaluating re-identification risks scores in publicly available clinical trial datasets: Insights and implications by Aryelly Rodriguez, Linda J Williams, Stephanie C Lewis, Pamela Sinclair, Sandra Eldridge, Tracy Jackson and Christopher J Weir in Clinical Trials

sj-docx-2-ctj-10.1177_17407745251356423 – Supplemental material for Evaluating re-identification risks scores in publicly available clinical trial datasets: Insights and implications Supplemental material, sj-docx-2-ctj-10.1177_17407745251356423 for Evaluating re-identification risks scores in publicly available clinical trial datasets: Insights and implications by Aryelly Rodriguez, Linda J Williams, Stephanie C Lewis, Pamela Sinclair, Sandra Eldridge, Tracy Jackson and Christopher J Weir in Clinical Trials

sj-docx-3-ctj-10.1177_17407745251356423 – Supplemental material for Evaluating re-identification risks scores in publicly available clinical trial datasets: Insights and implications Supplemental material, sj-docx-3-ctj-10.1177_17407745251356423 for Evaluating re-identification risks scores in publicly available clinical trial datasets: Insights and implications by Aryelly Rodriguez, Linda J Williams, Stephanie C Lewis, Pamela Sinclair, Sandra Eldridge, Tracy Jackson and Christopher J Weir in Clinical Trials

sj-docx-4-ctj-10.1177_17407745251356423 – Supplemental material for Evaluating re-identification risks scores in publicly available clinical trial datasets: Insights and implications Supplemental material, sj-docx-4-ctj-10.1177_17407745251356423 for Evaluating re-identification risks scores in publicly available clinical trial datasets: Insights and implications by Aryelly Rodriguez, Linda J Williams, Stephanie C Lewis, Pamela Sinclair, Sandra Eldridge, Tracy Jackson and Christopher J Weir in Clinical Trials

sj-docx-5-ctj-10.1177_17407745251356423 – Supplemental material for Evaluating re-identification risks scores in publicly available clinical trial datasets: Insights and implications Supplemental material, sj-docx-5-ctj-10.1177_17407745251356423 for Evaluating re-identification risks scores in publicly available clinical trial datasets: Insights and implications by Aryelly Rodriguez, Linda J Williams, Stephanie C Lewis, Pamela Sinclair, Sandra Eldridge, Tracy Jackson and Christopher J Weir in Clinical Trials

sj-docx-6-ctj-10.1177_17407745251356423 – Supplemental material for Evaluating re-identification risks scores in publicly available clinical trial datasets: Insights and implications Supplemental material, sj-docx-6-ctj-10.1177_17407745251356423 for Evaluating re-identification risks scores in publicly available clinical trial datasets: Insights and implications by Aryelly Rodriguez, Linda J Williams, Stephanie C Lewis, Pamela Sinclair, Sandra Eldridge, Tracy Jackson and Christopher J Weir in Clinical Trials

sj-docx-7-ctj-10.1177_17407745251356423 – Supplemental material for Evaluating re-identification risks scores in publicly available clinical trial datasets: Insights and implications Supplemental material, sj-docx-7-ctj-10.1177_17407745251356423 for Evaluating re-identification risks scores in publicly available clinical trial datasets: Insights and implications by Aryelly Rodriguez, Linda J Williams, Stephanie C Lewis, Pamela Sinclair, Sandra Eldridge, Tracy Jackson and Christopher J Weir in Clinical Trials
