# Supplementary figures and images for: Evaluating re-identification risks scores in publicly available clinical trial datasets: Insights and implications
Source: Clin Trials. 2025 Aug 22;22(6):649–66. doi: 10.1177/17407745251356423 (PMC12647387; doi:10.1177/17407745251356423)

Appendix 1 Study protocol


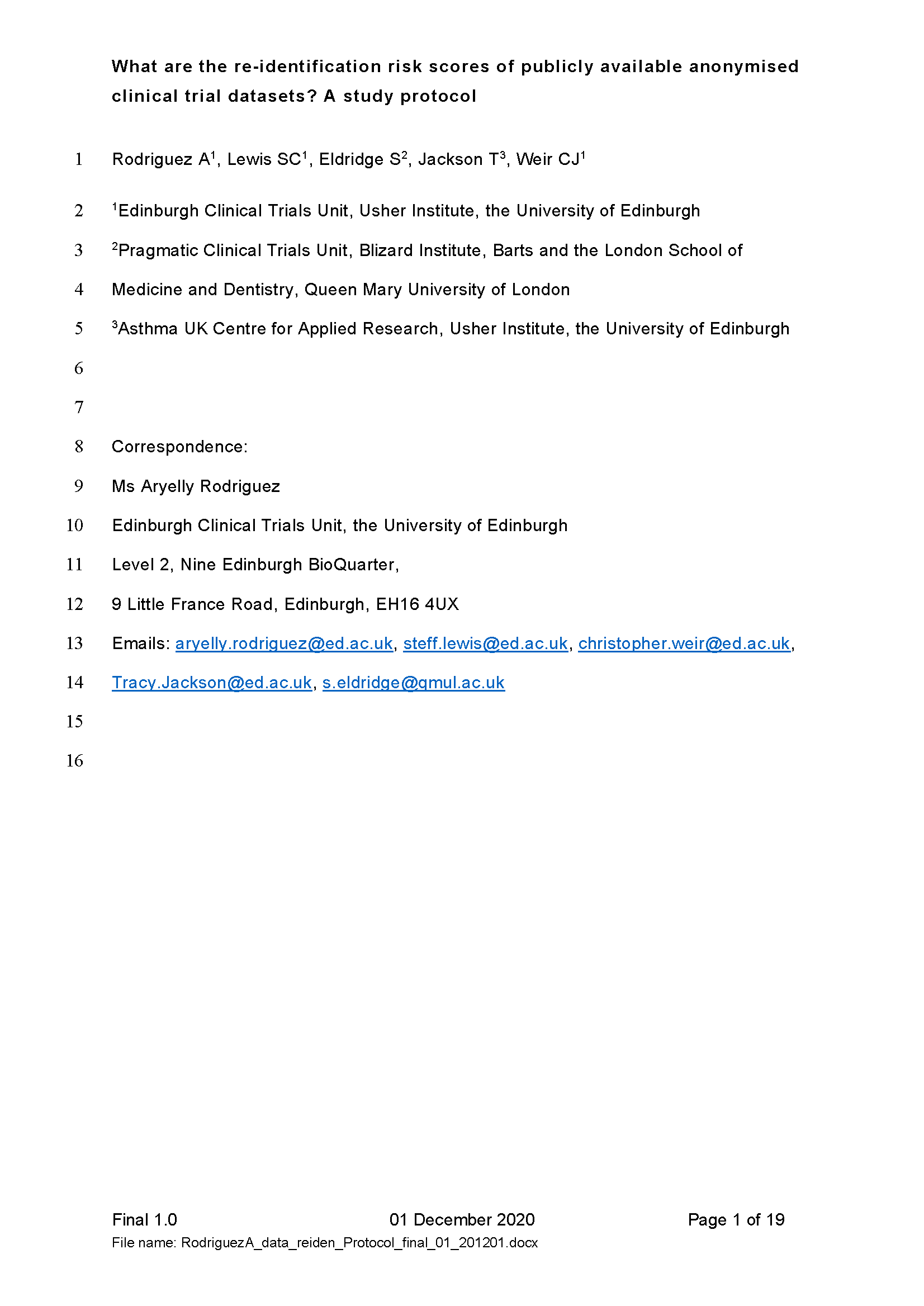


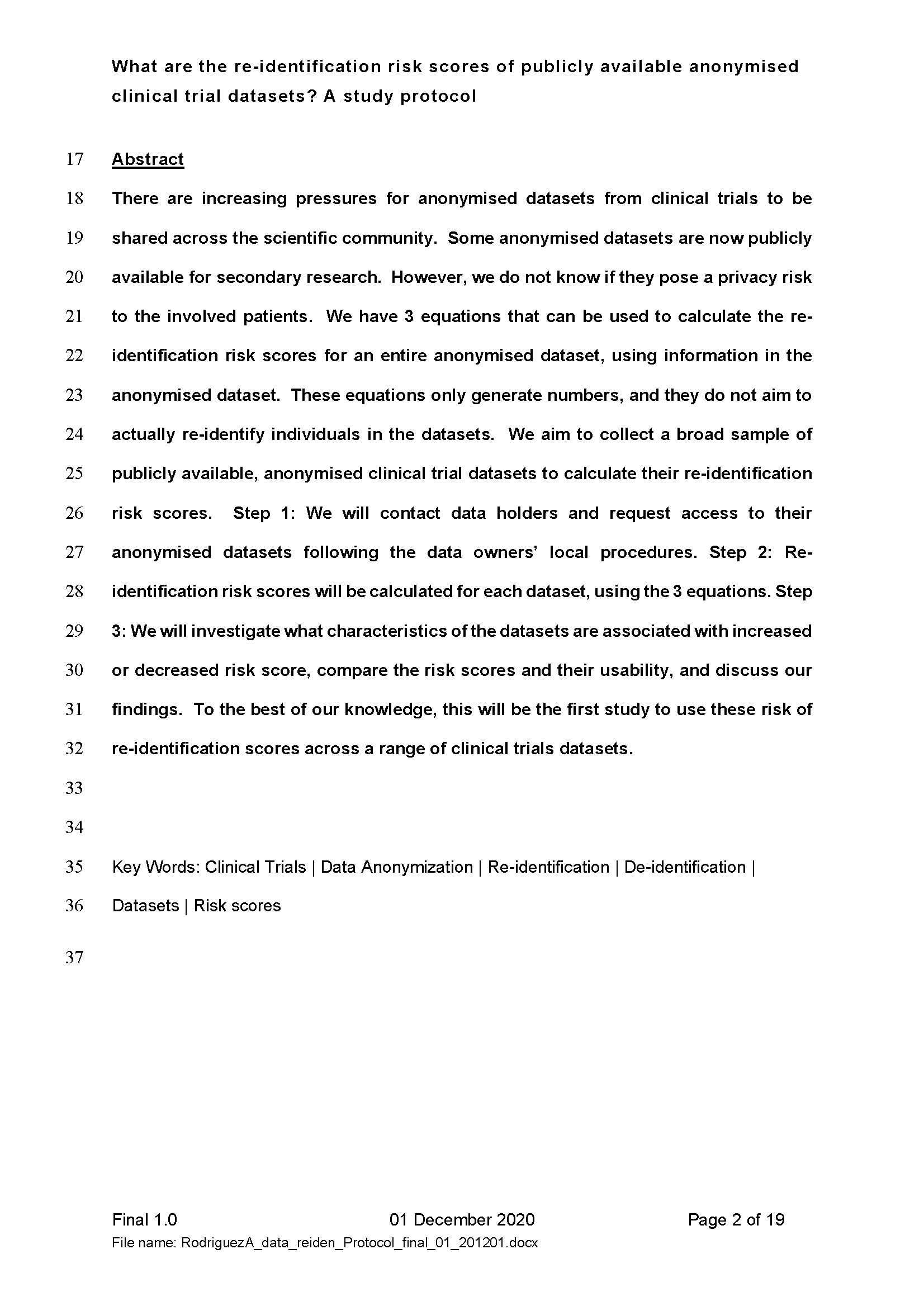


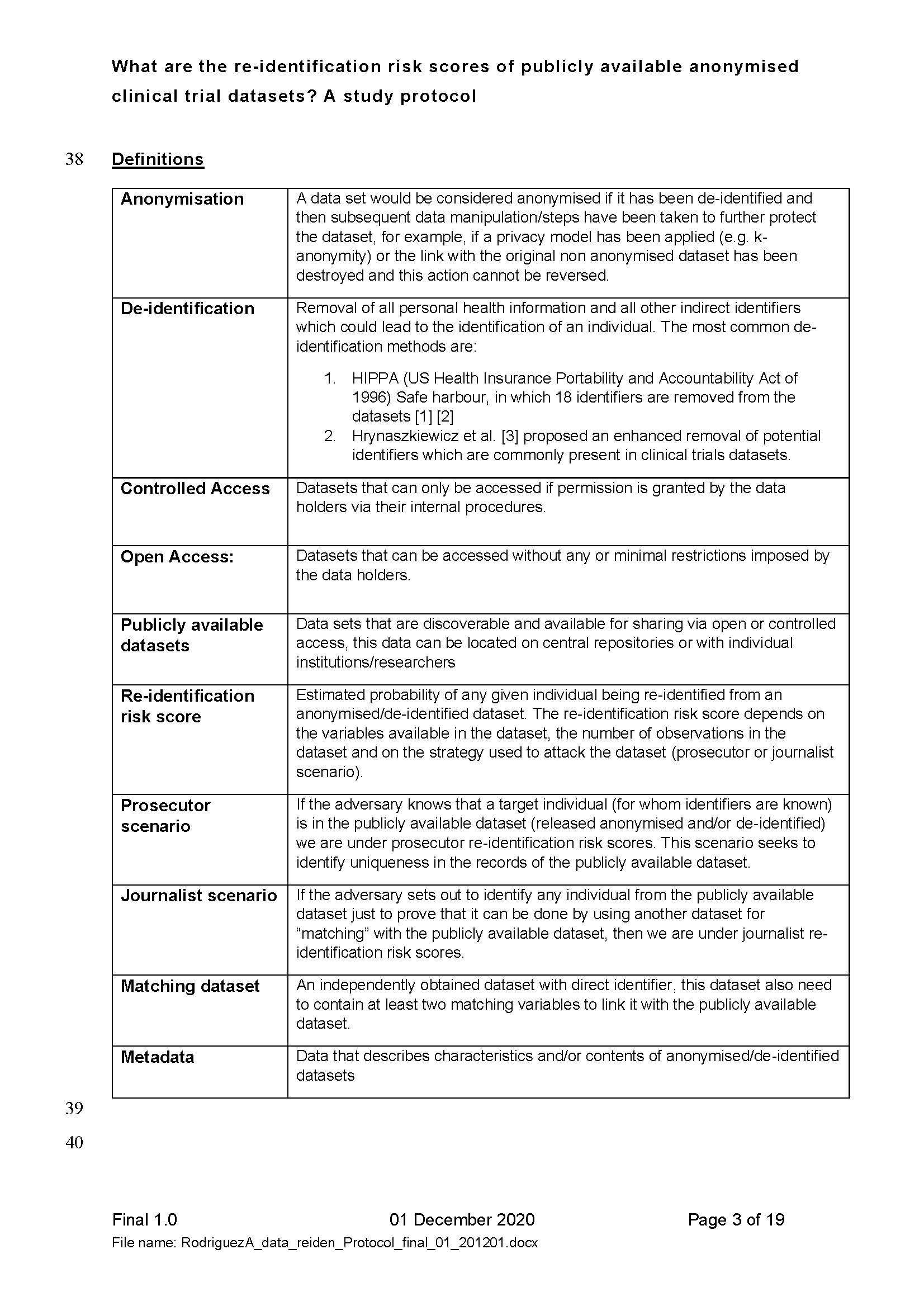


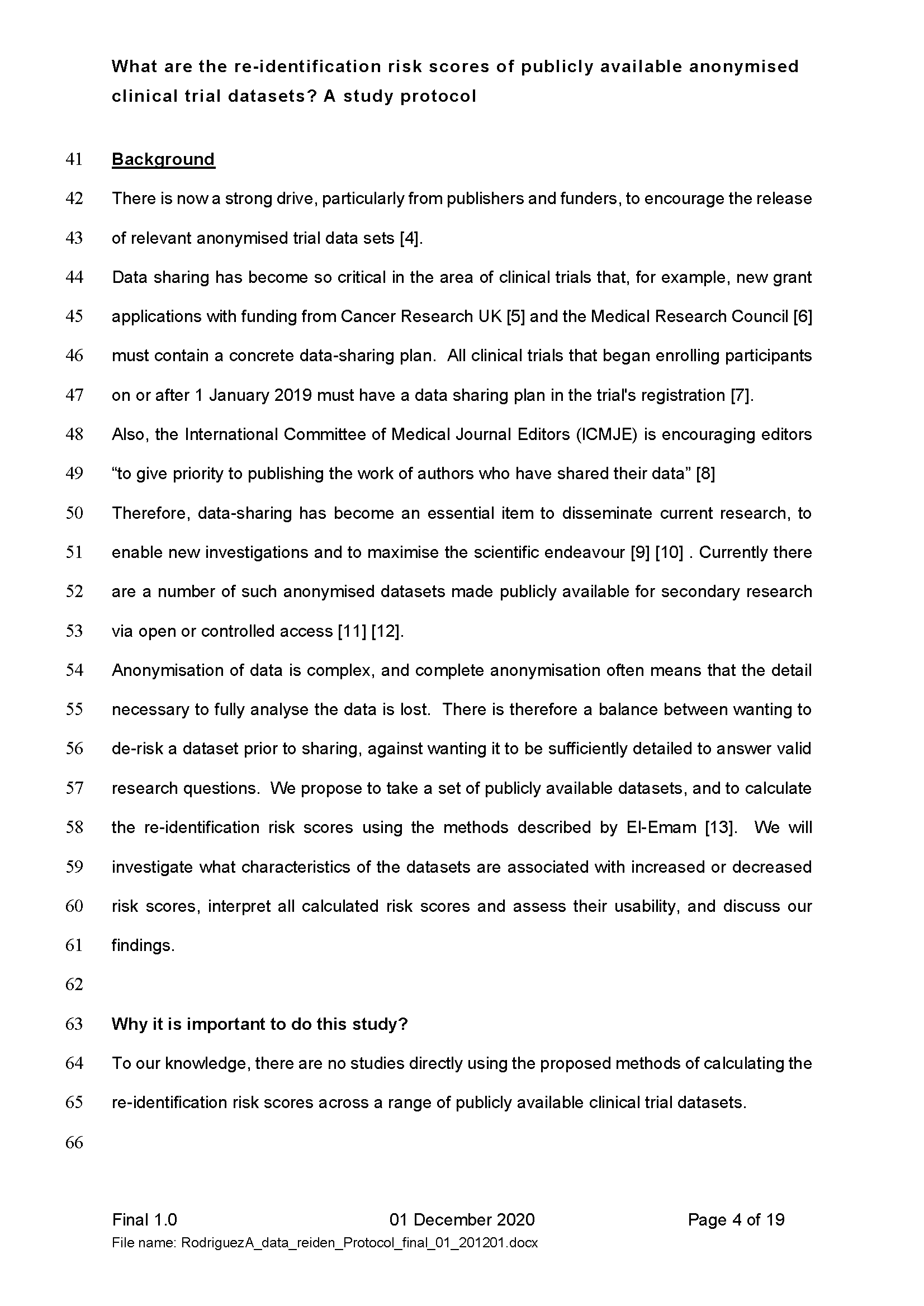


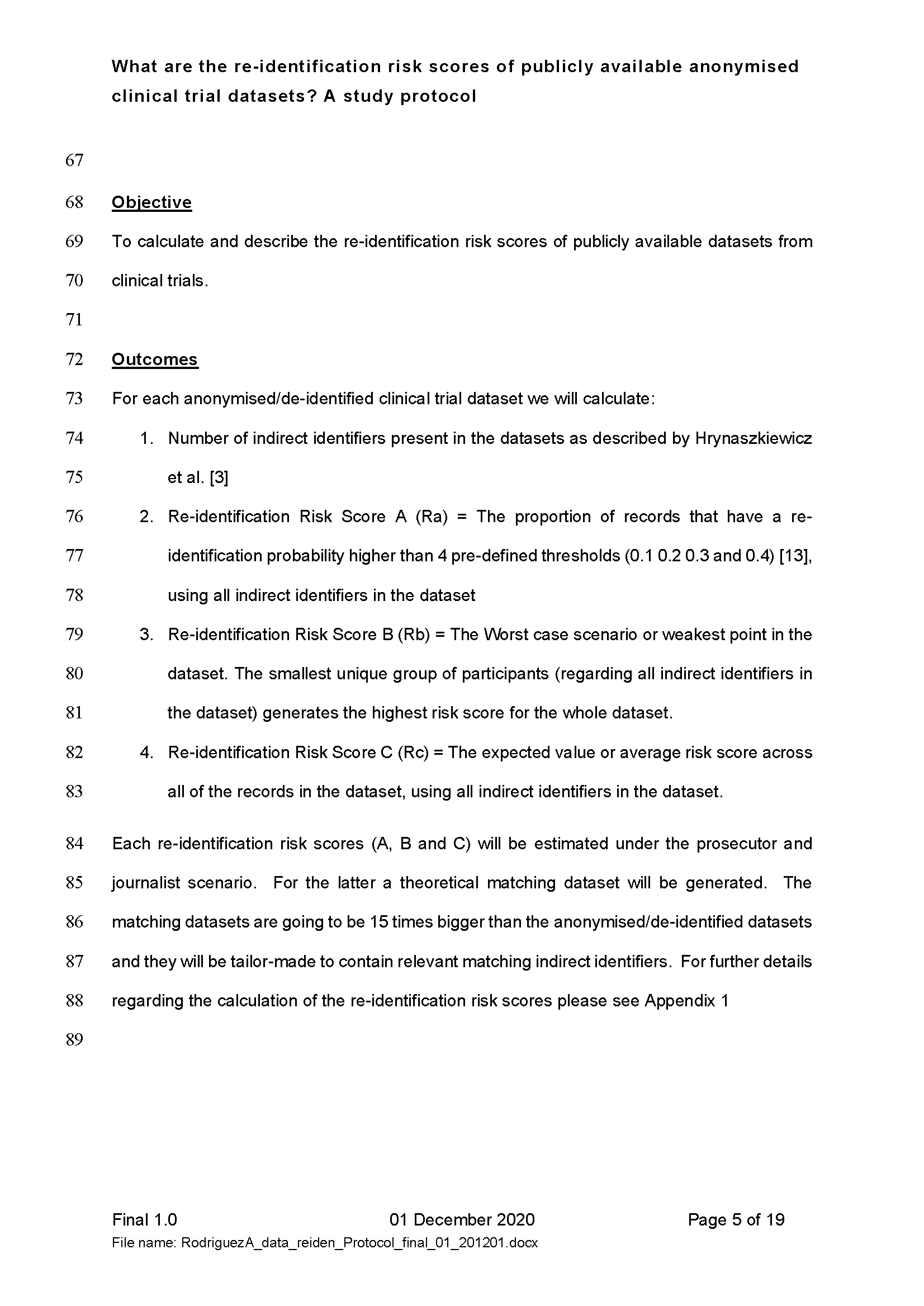


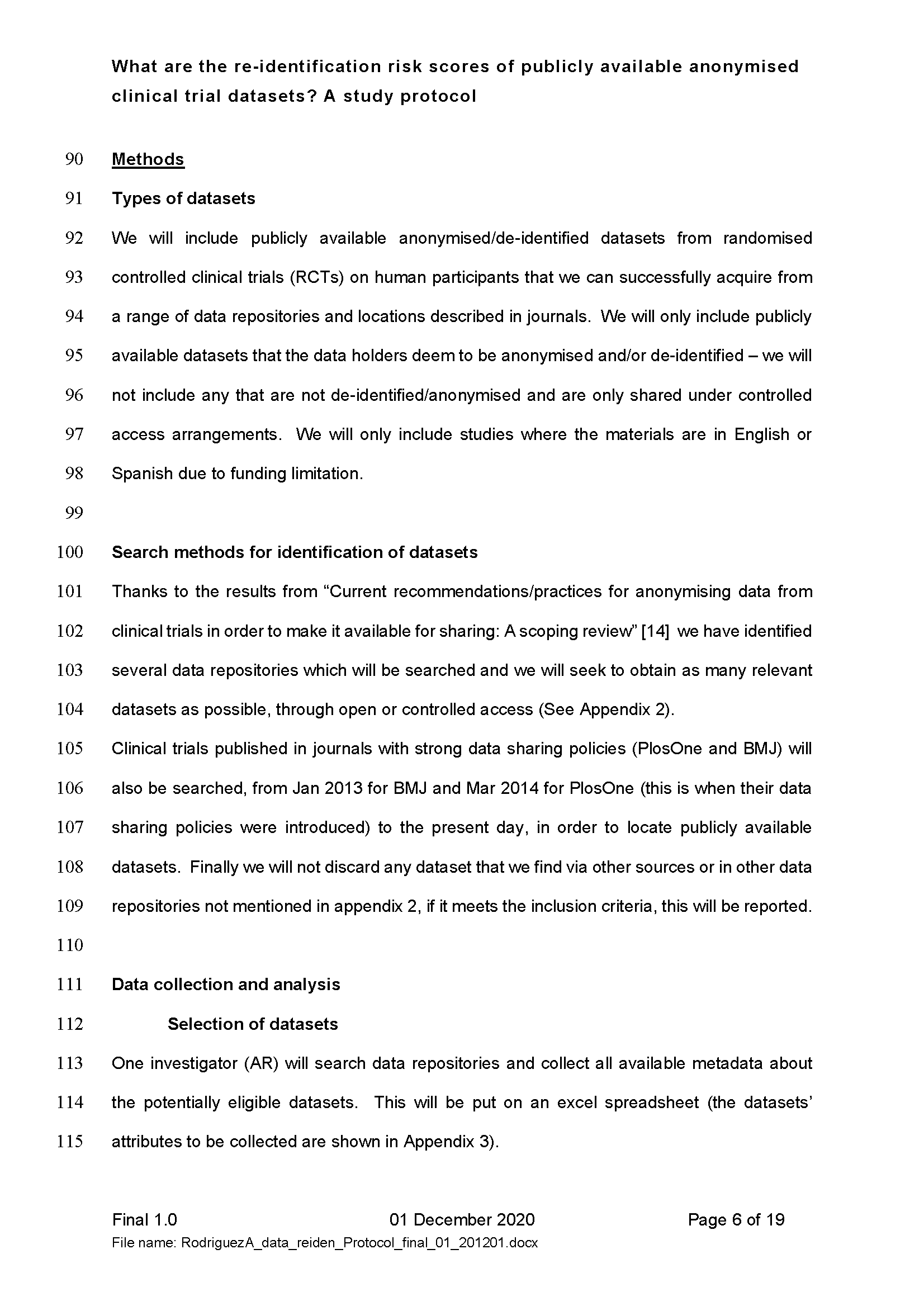


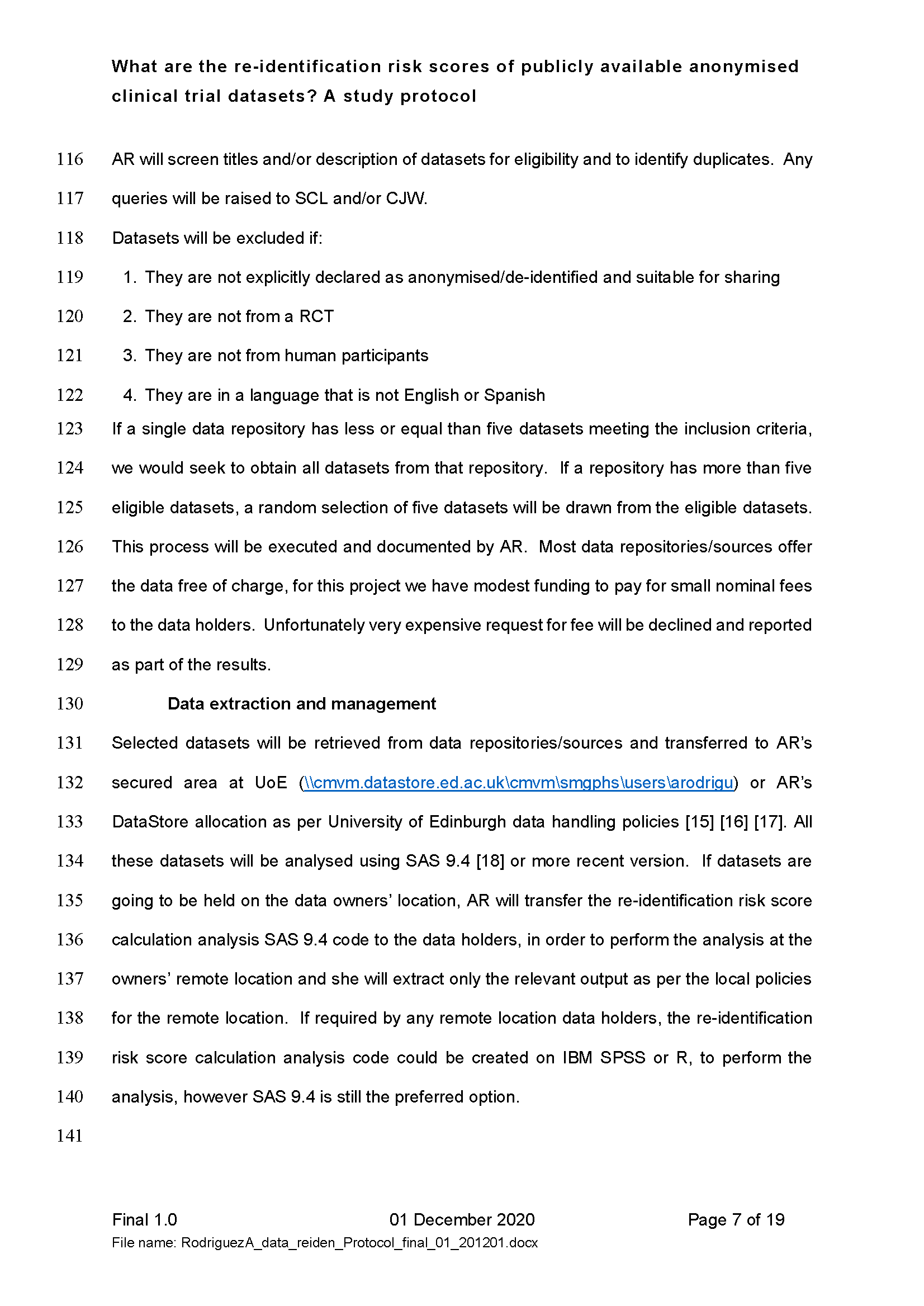


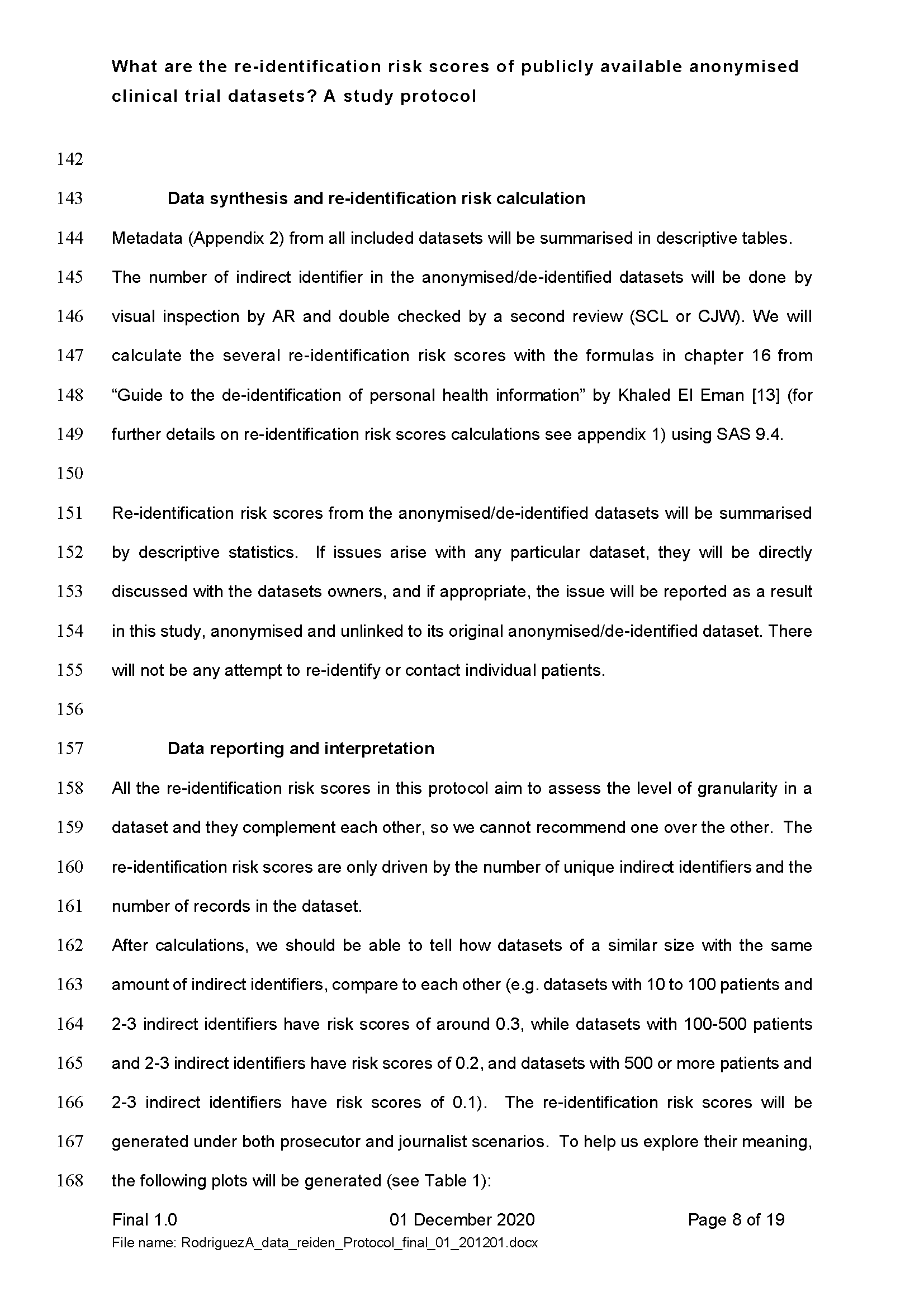


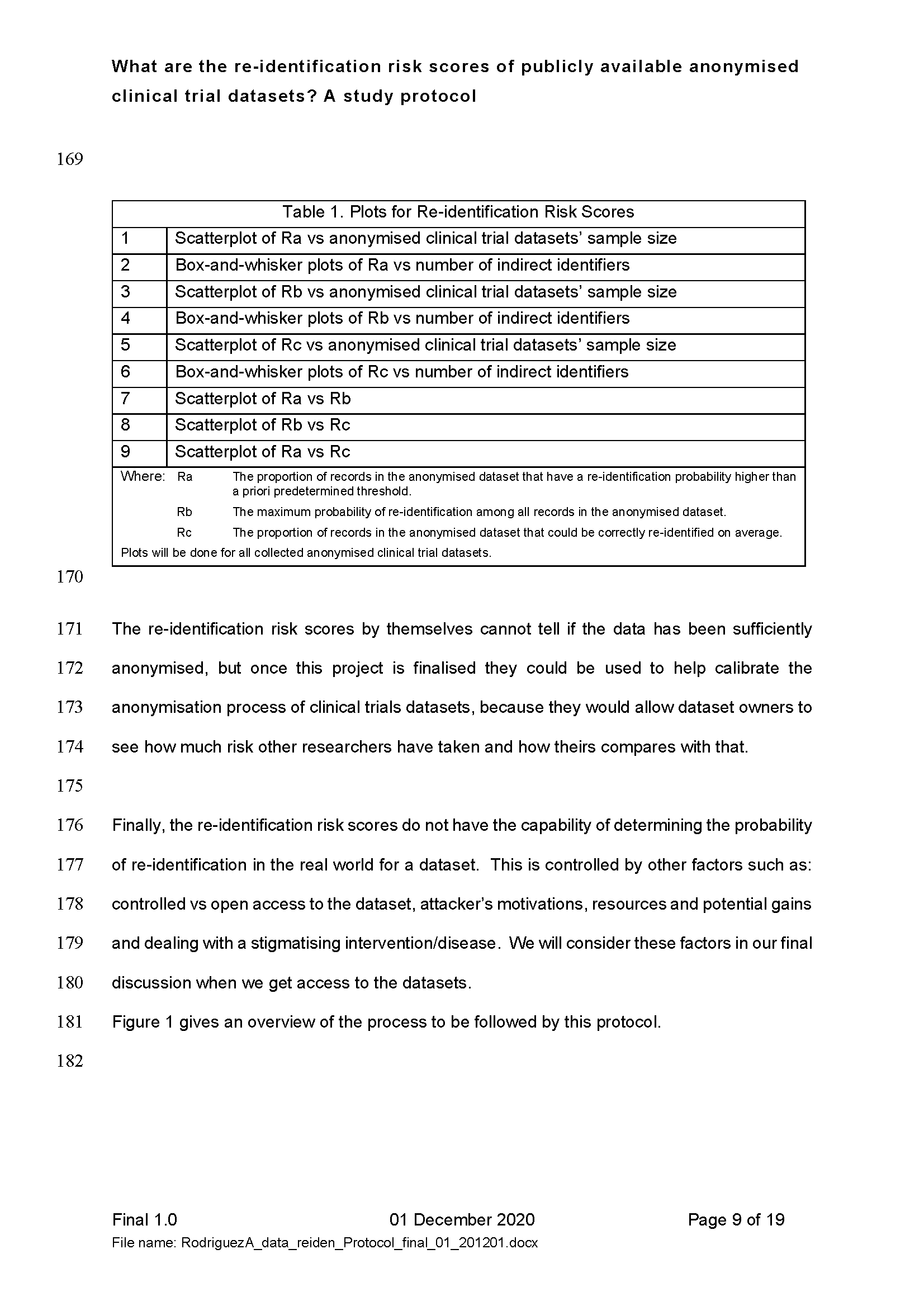


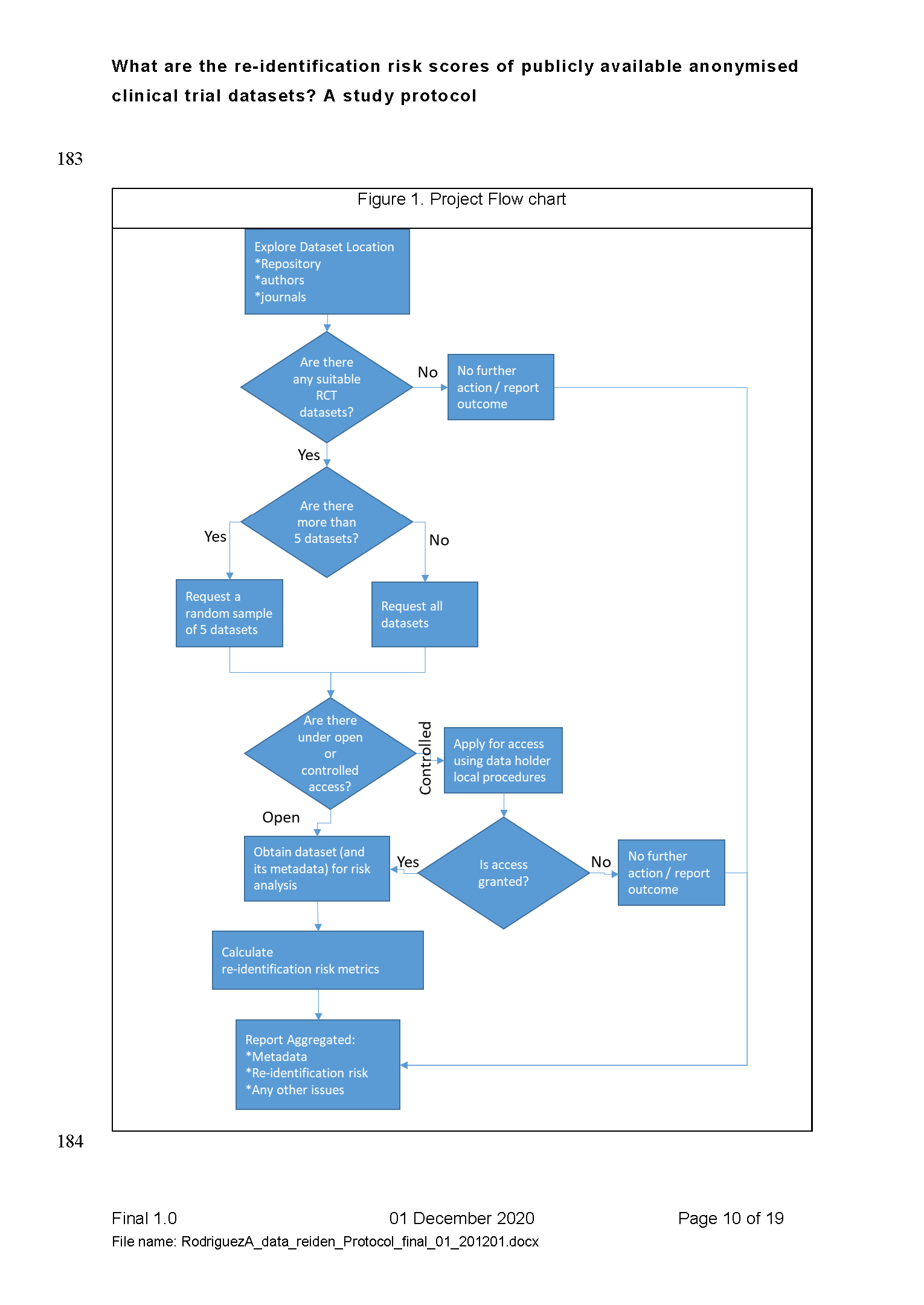


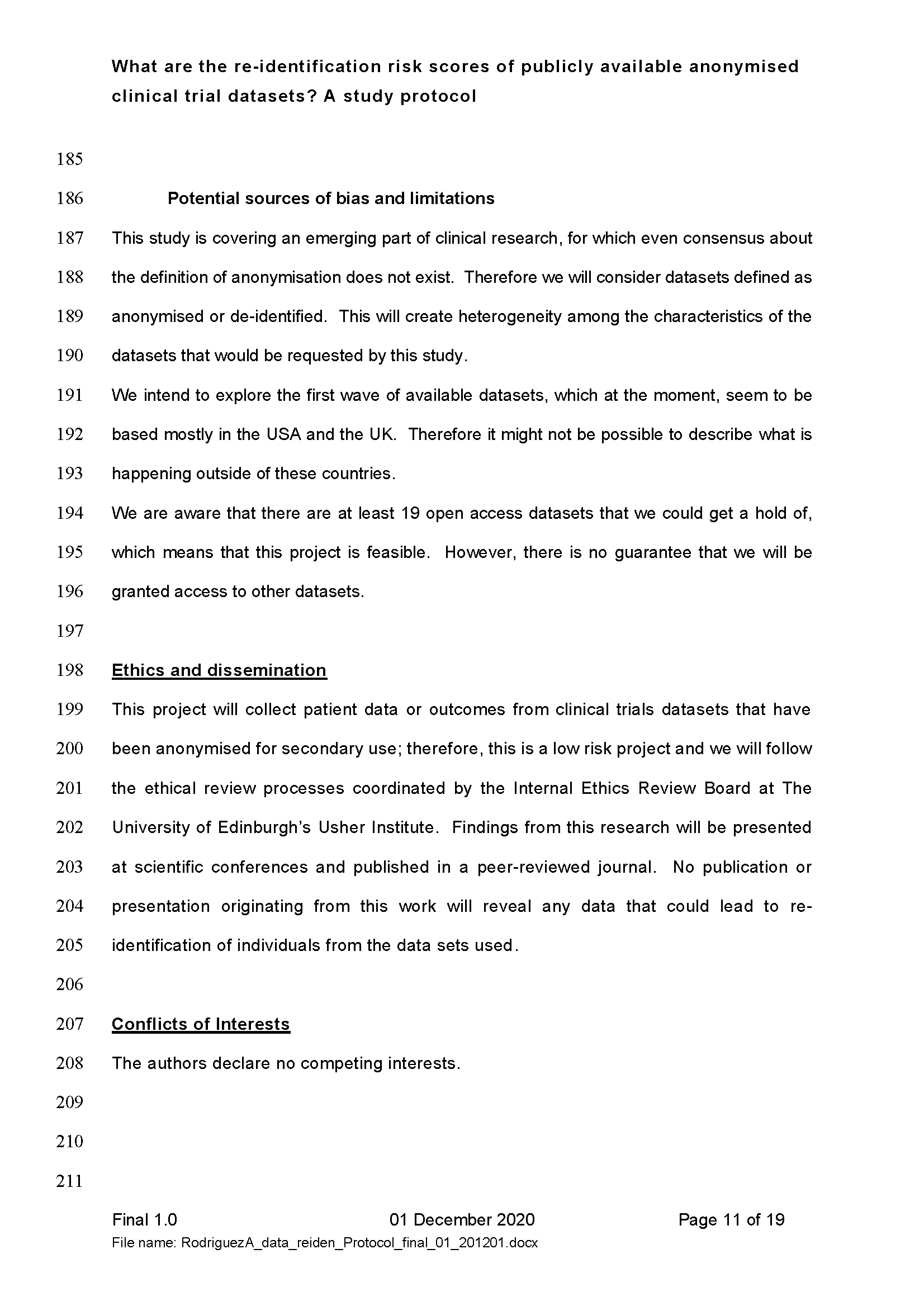


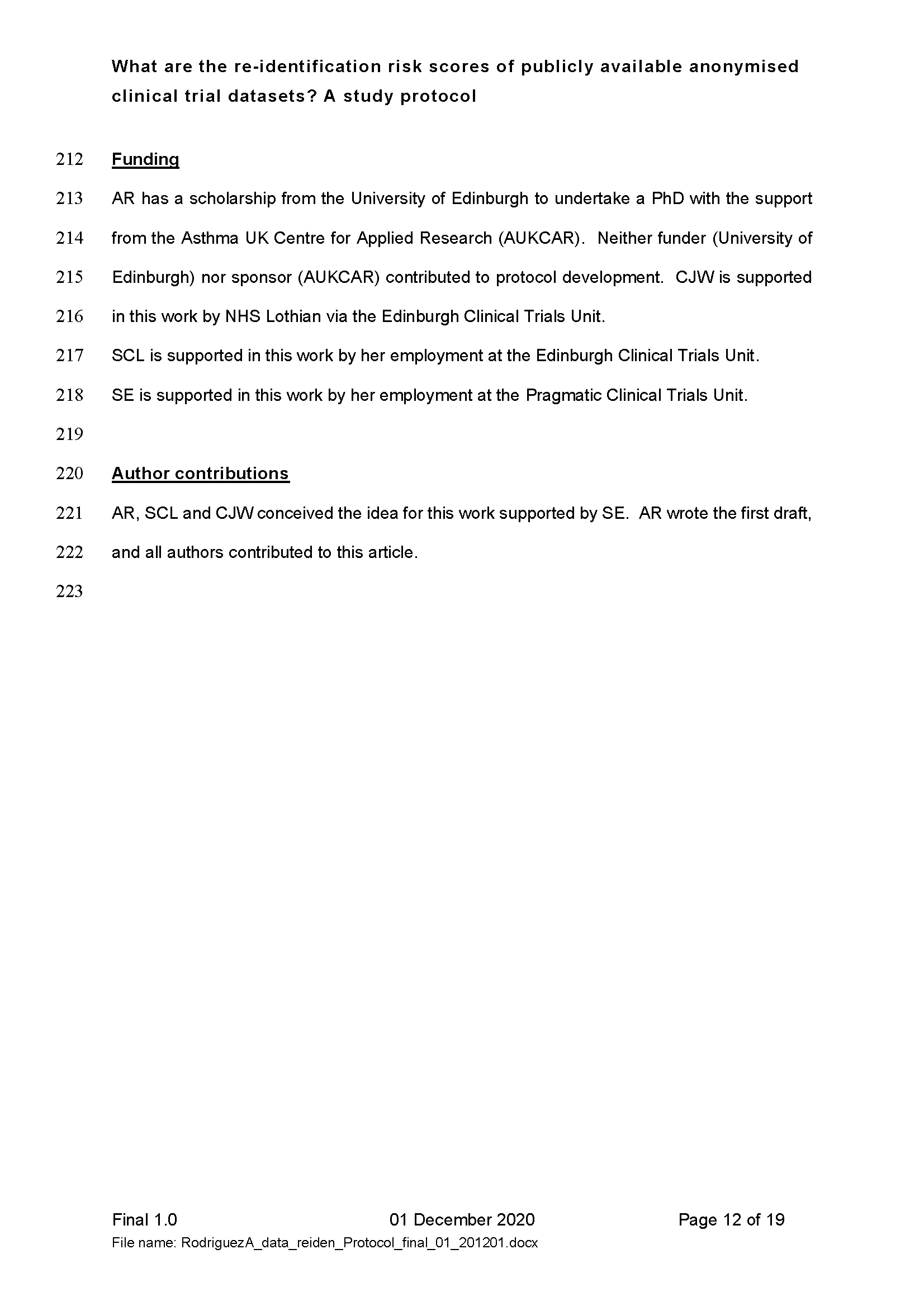


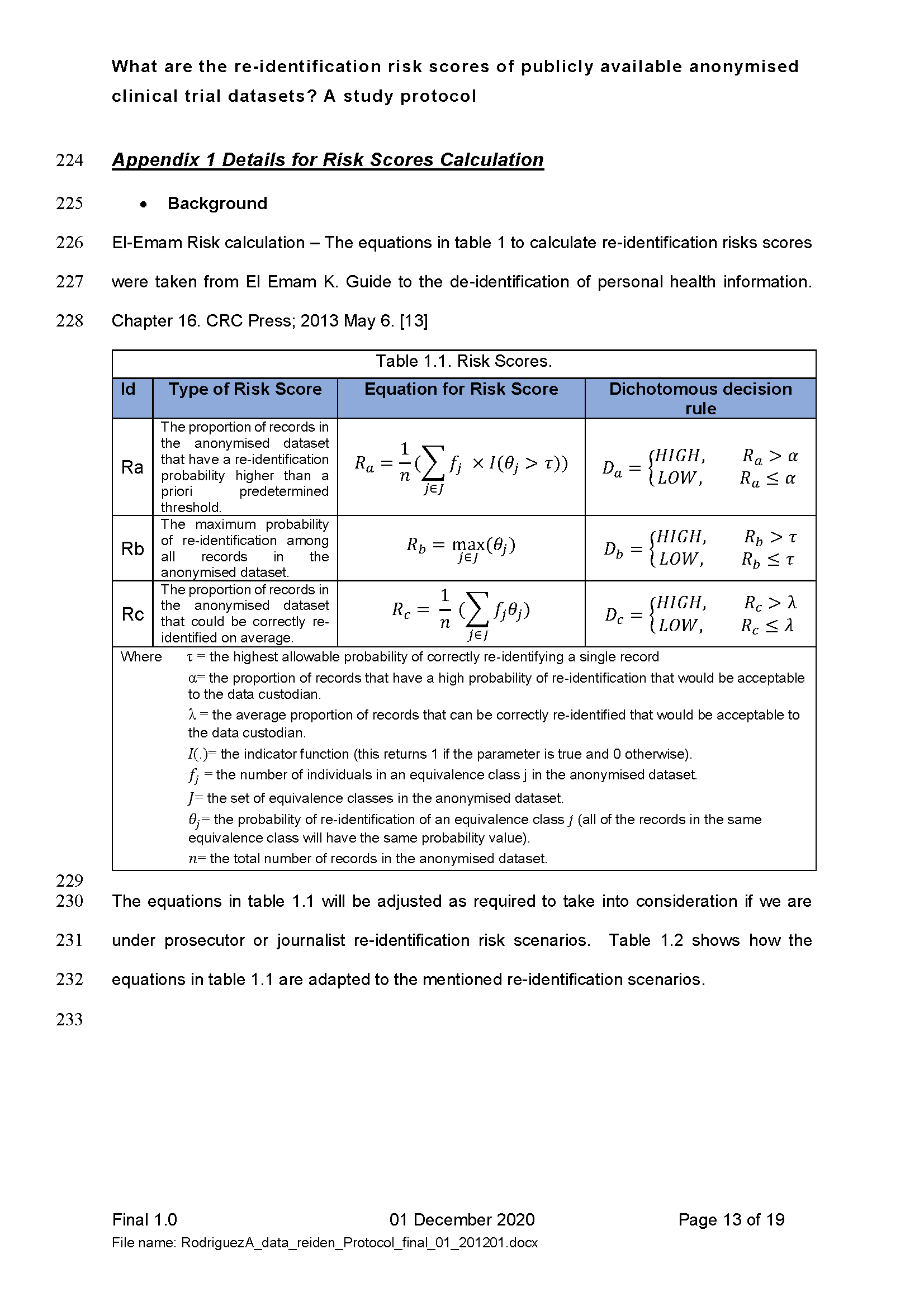


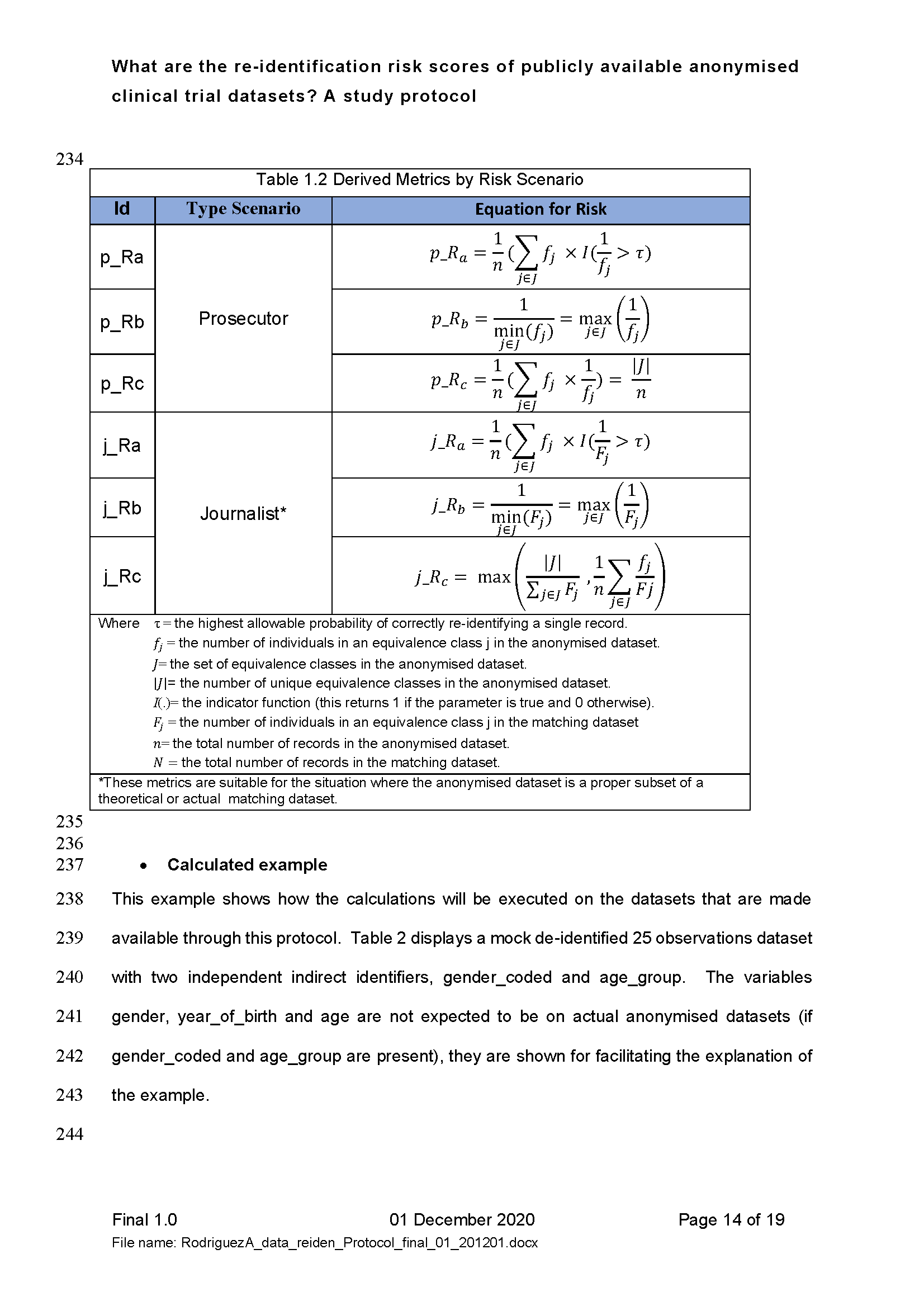


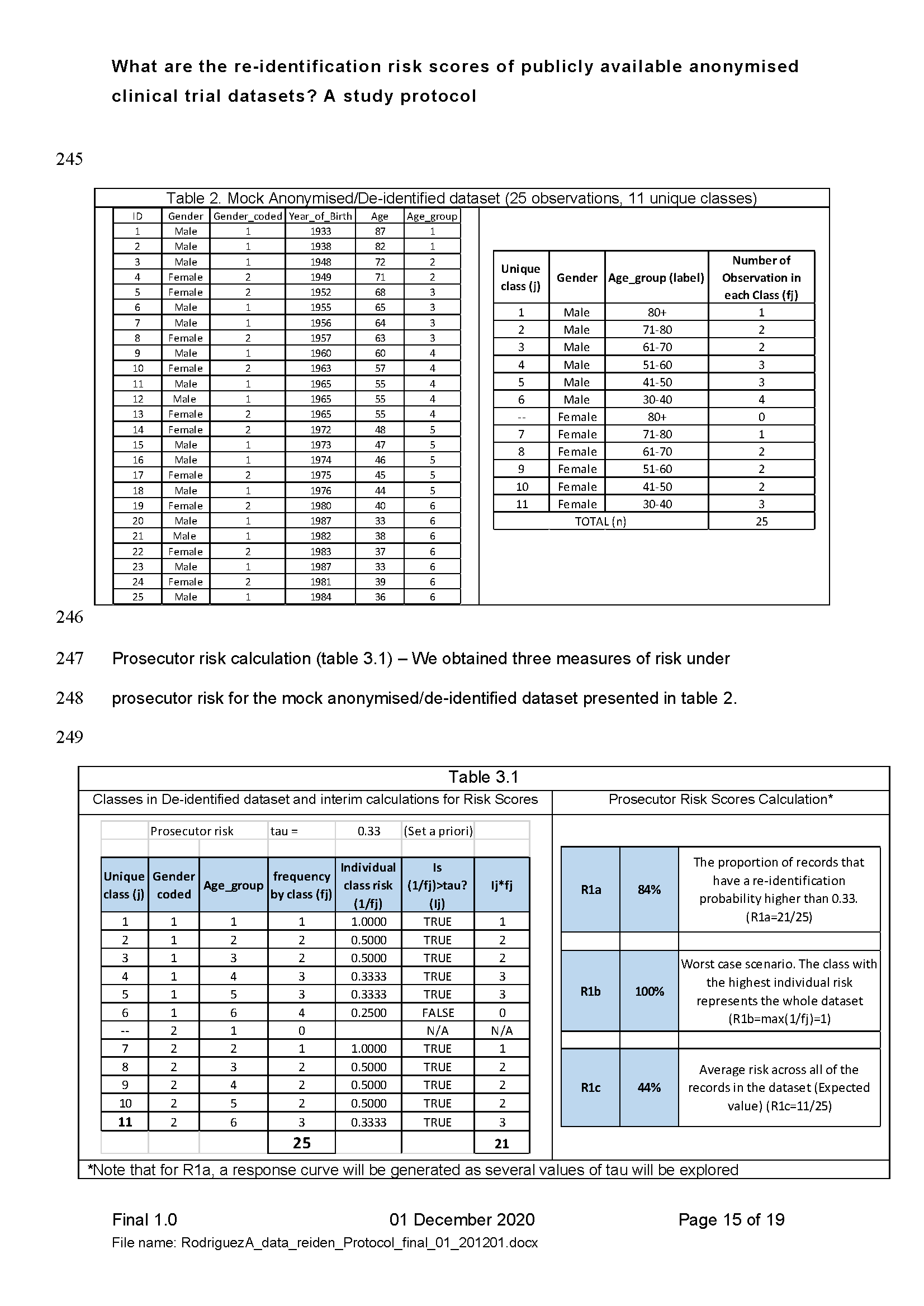


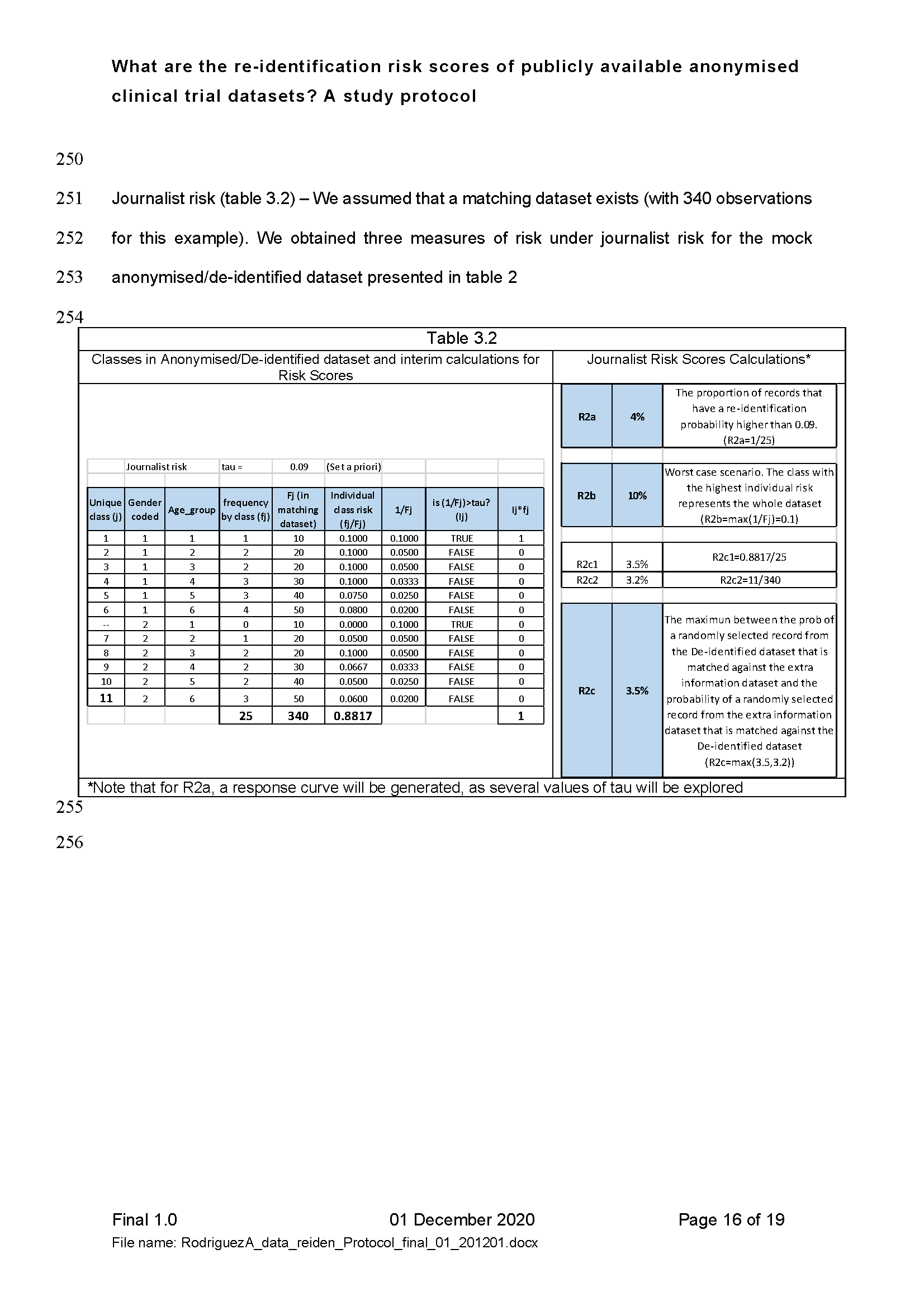


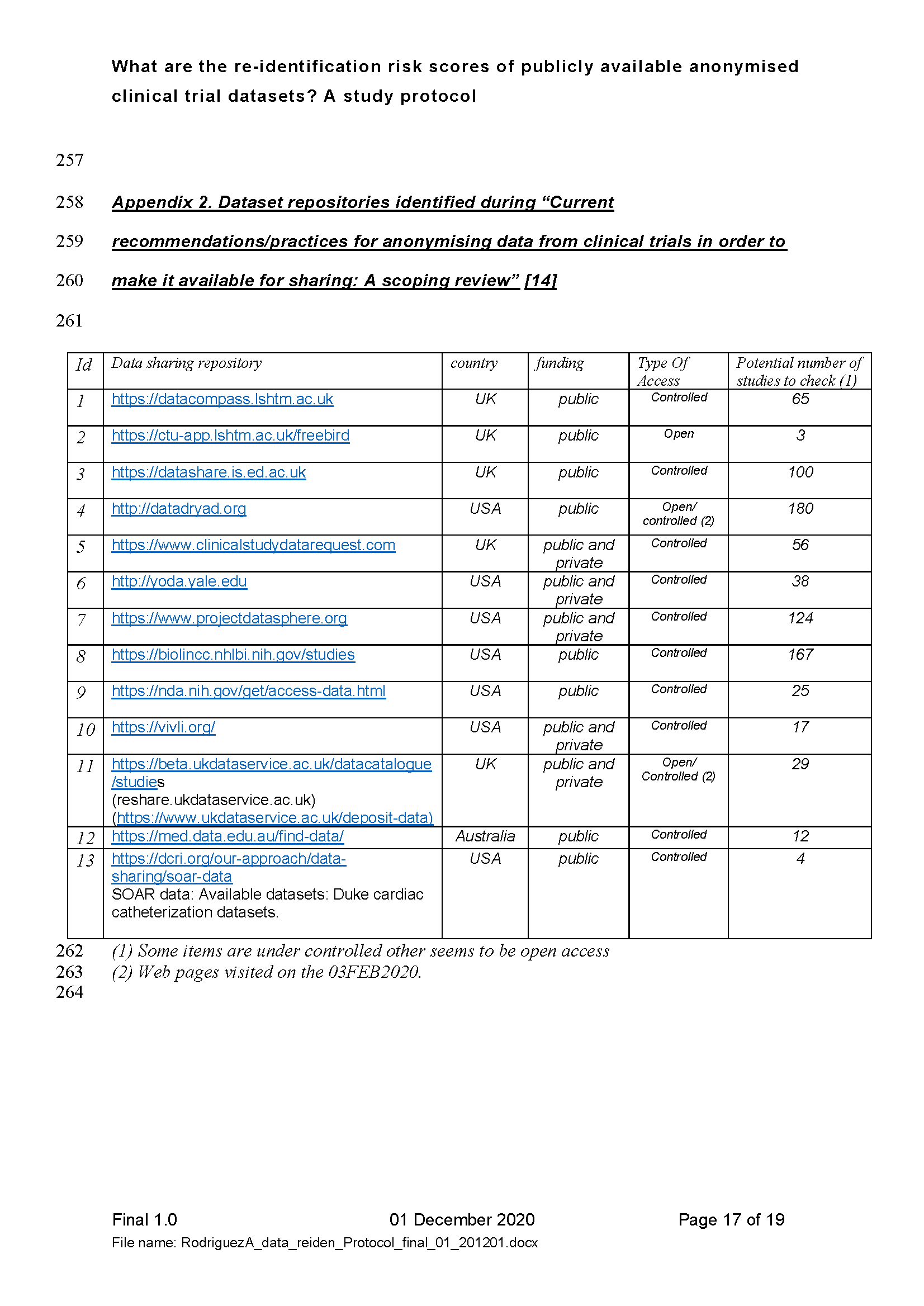


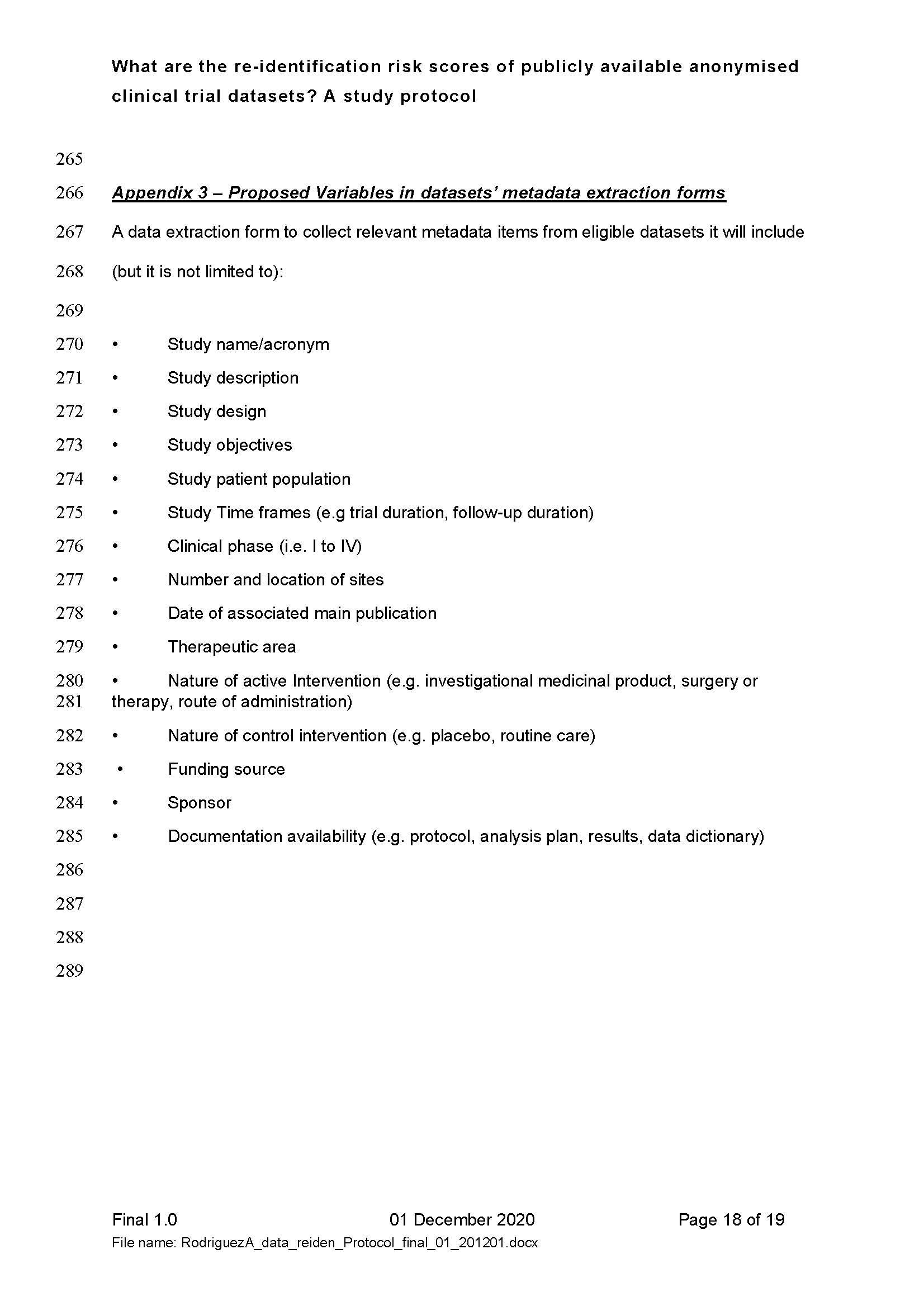


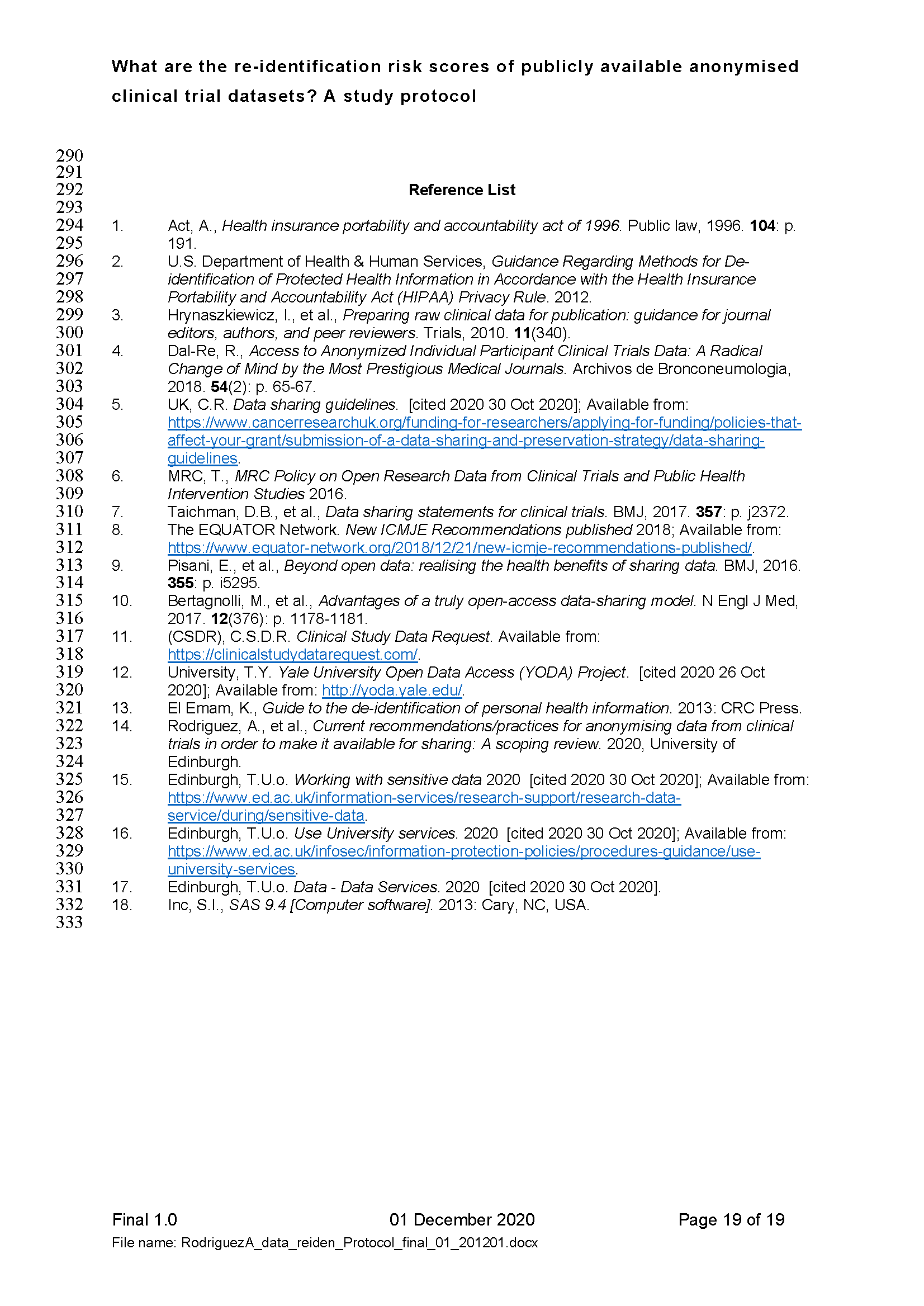

Supplement: sj-docx-1-ctj-10.1177_17407745251356423 – Supplemental material for Evaluating re-identification risks scores in publicly available clinical trial datasets: Insights and implications [file sj-docx-1-ctj-10.1177_17407745251356423.docx]

Appendix 7 Ethics/data protection application


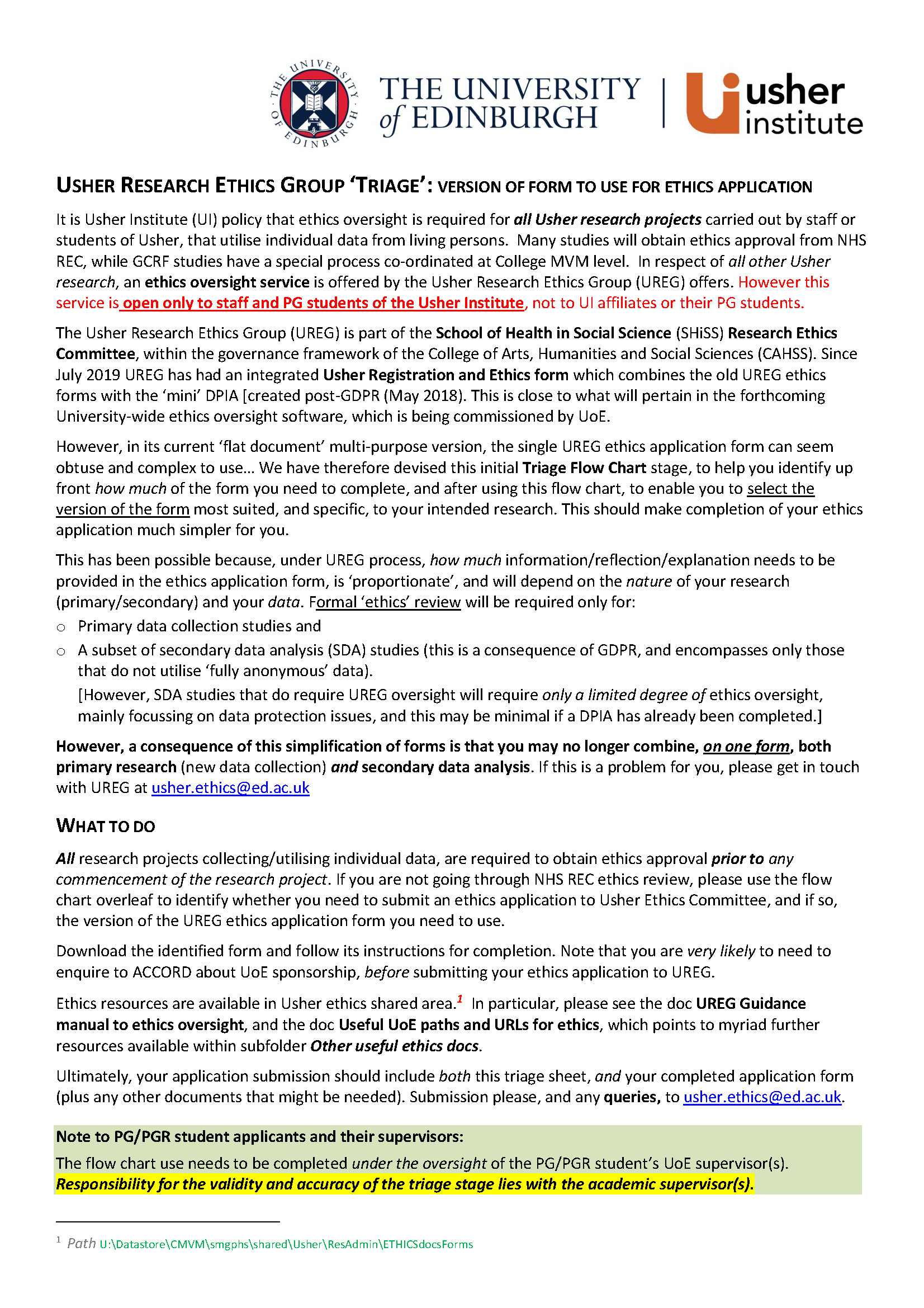


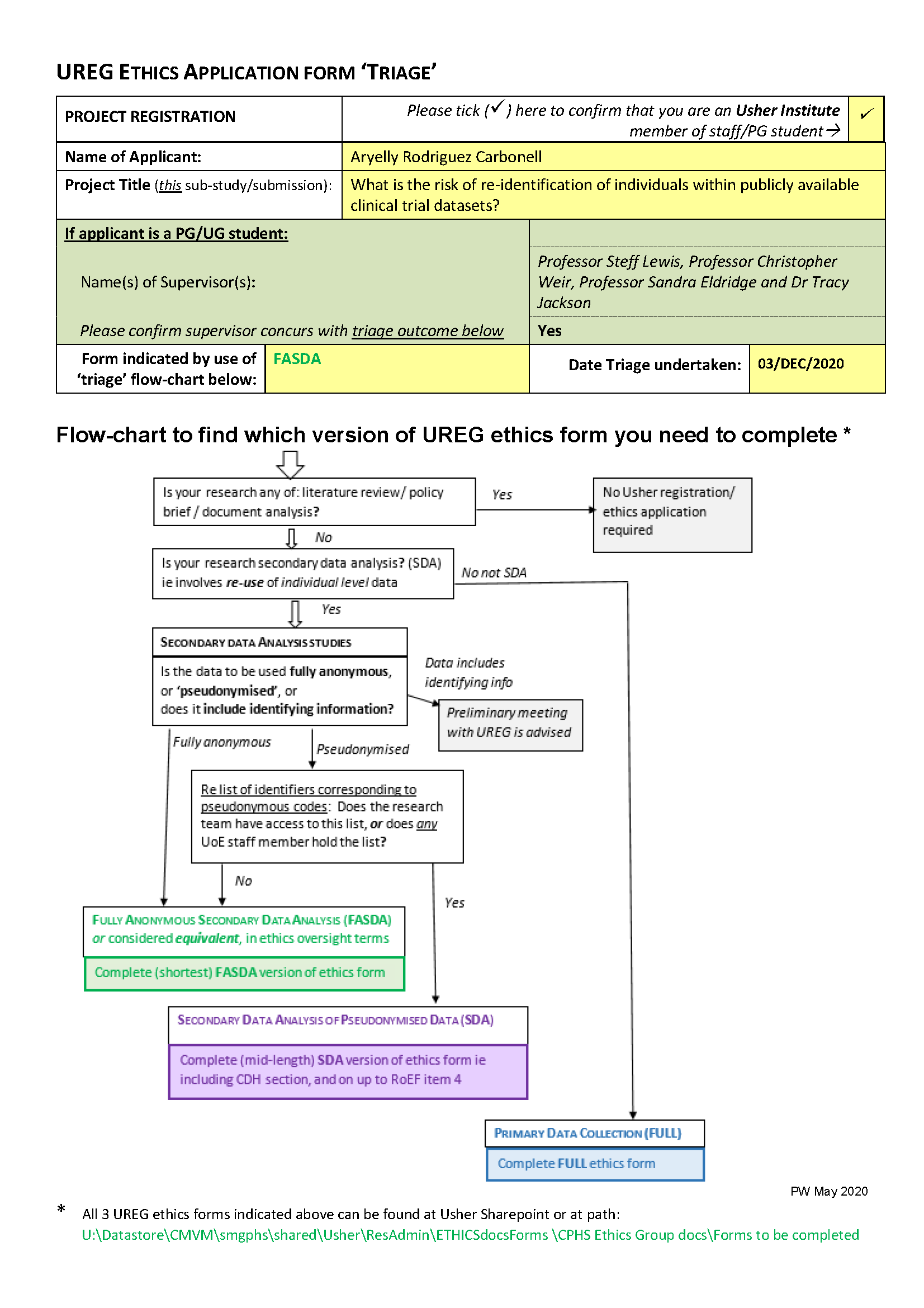


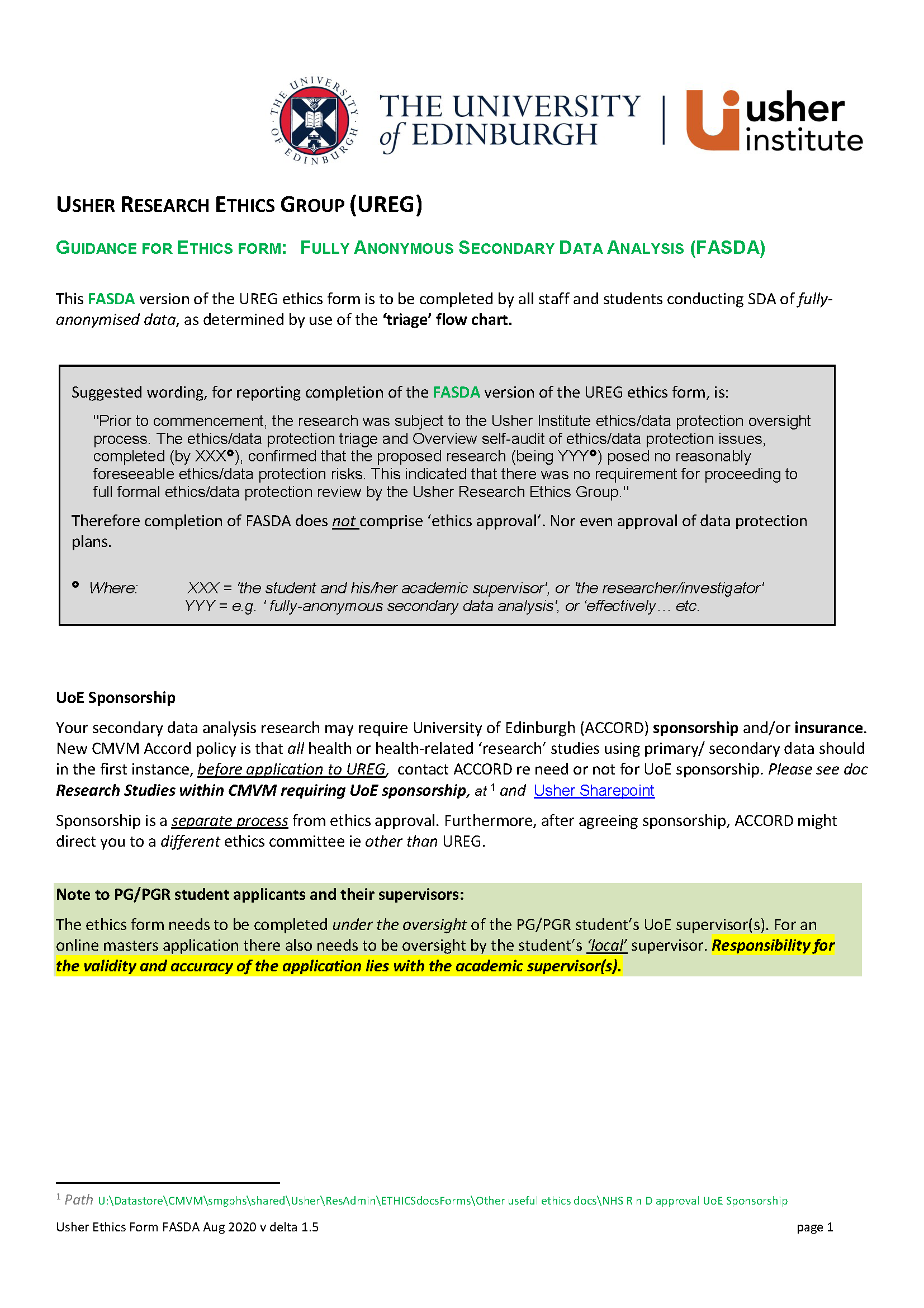


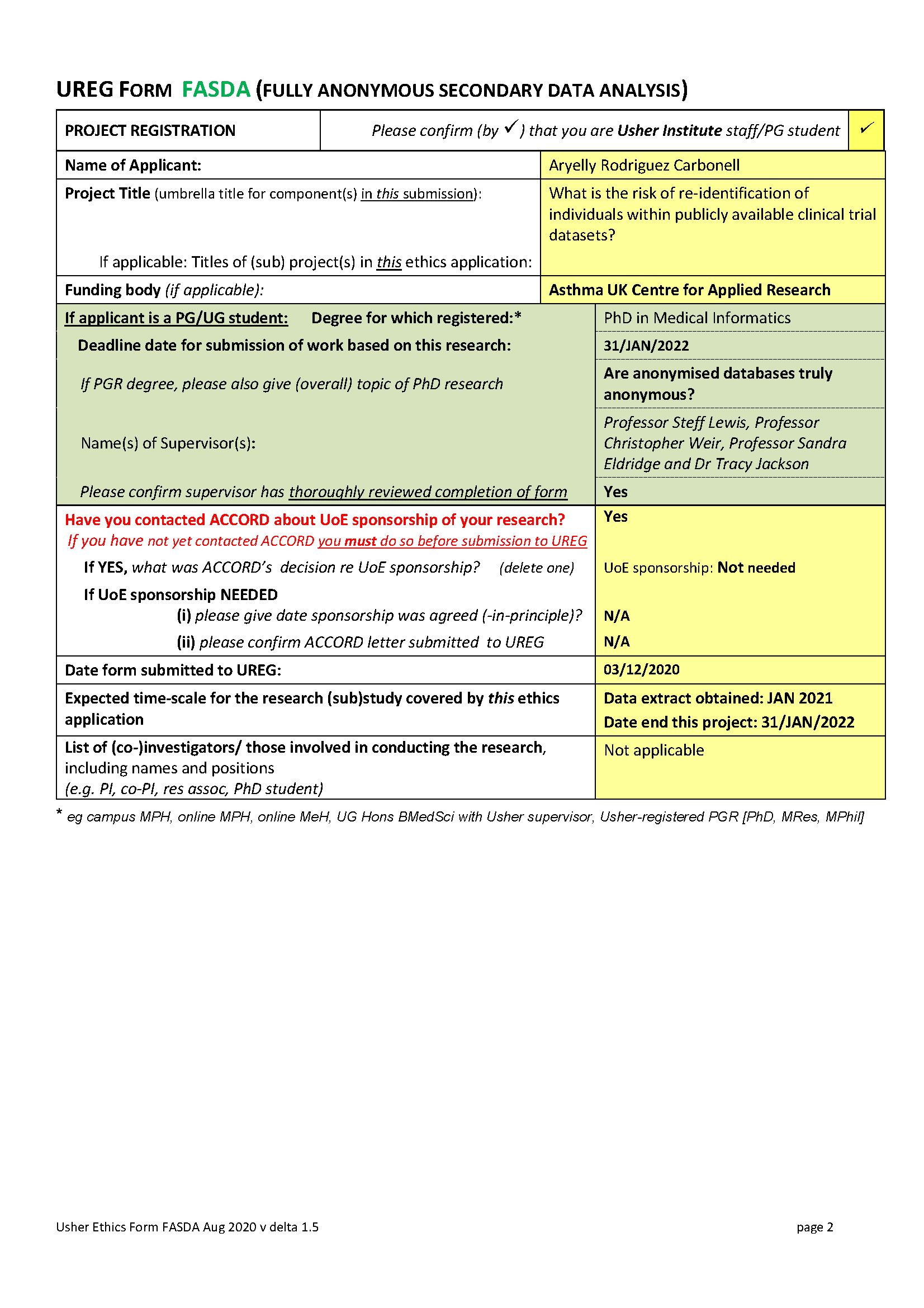


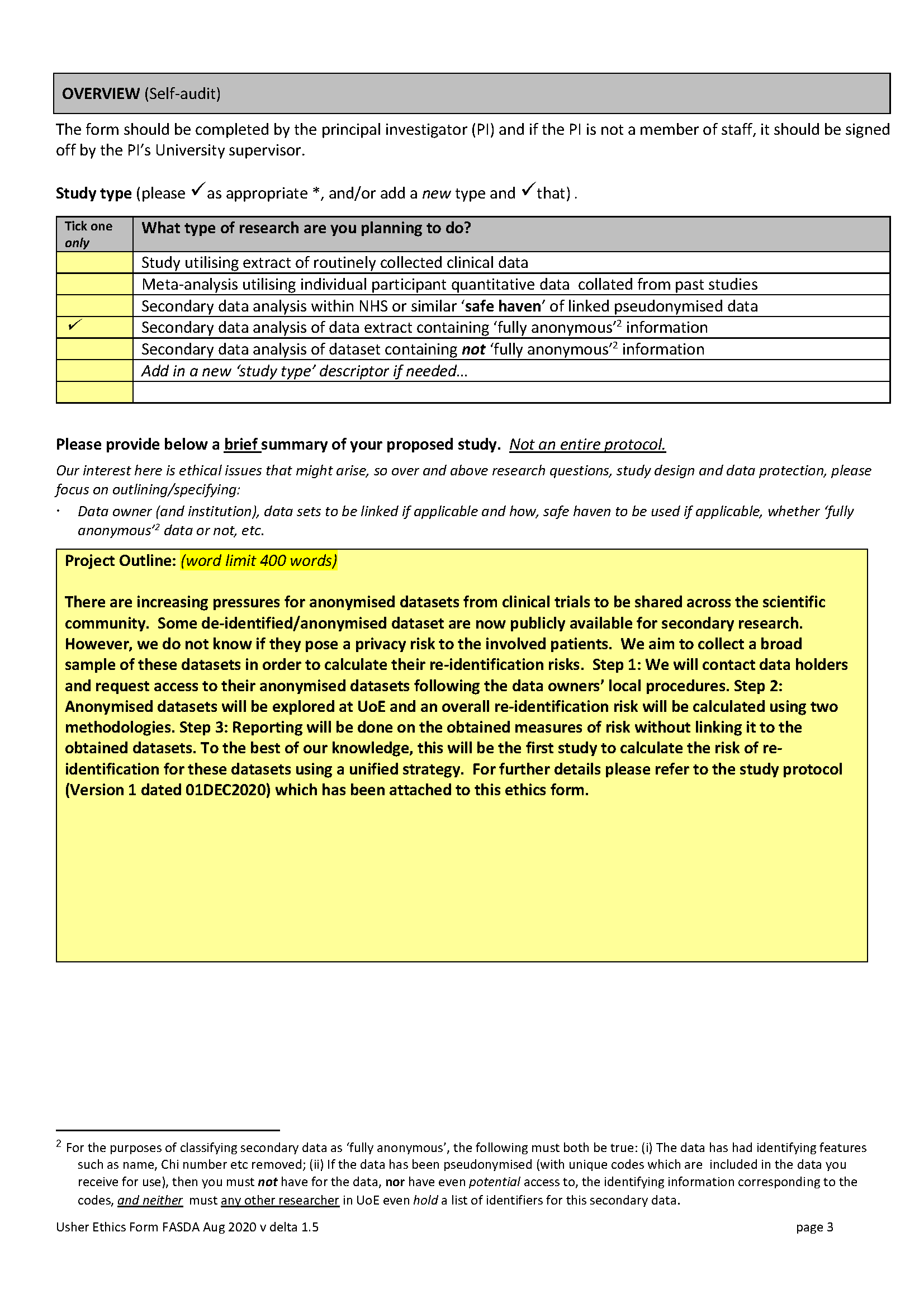


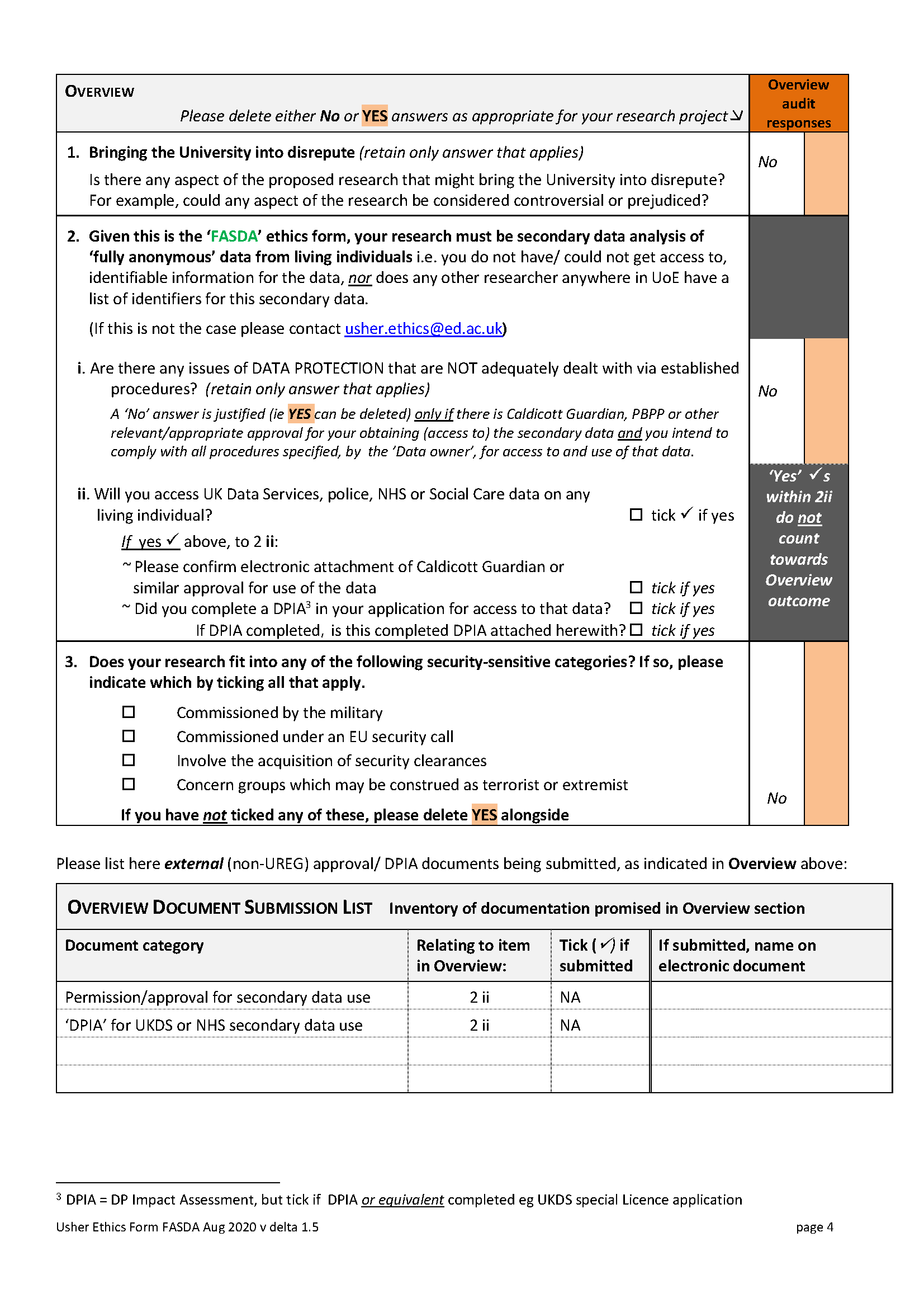


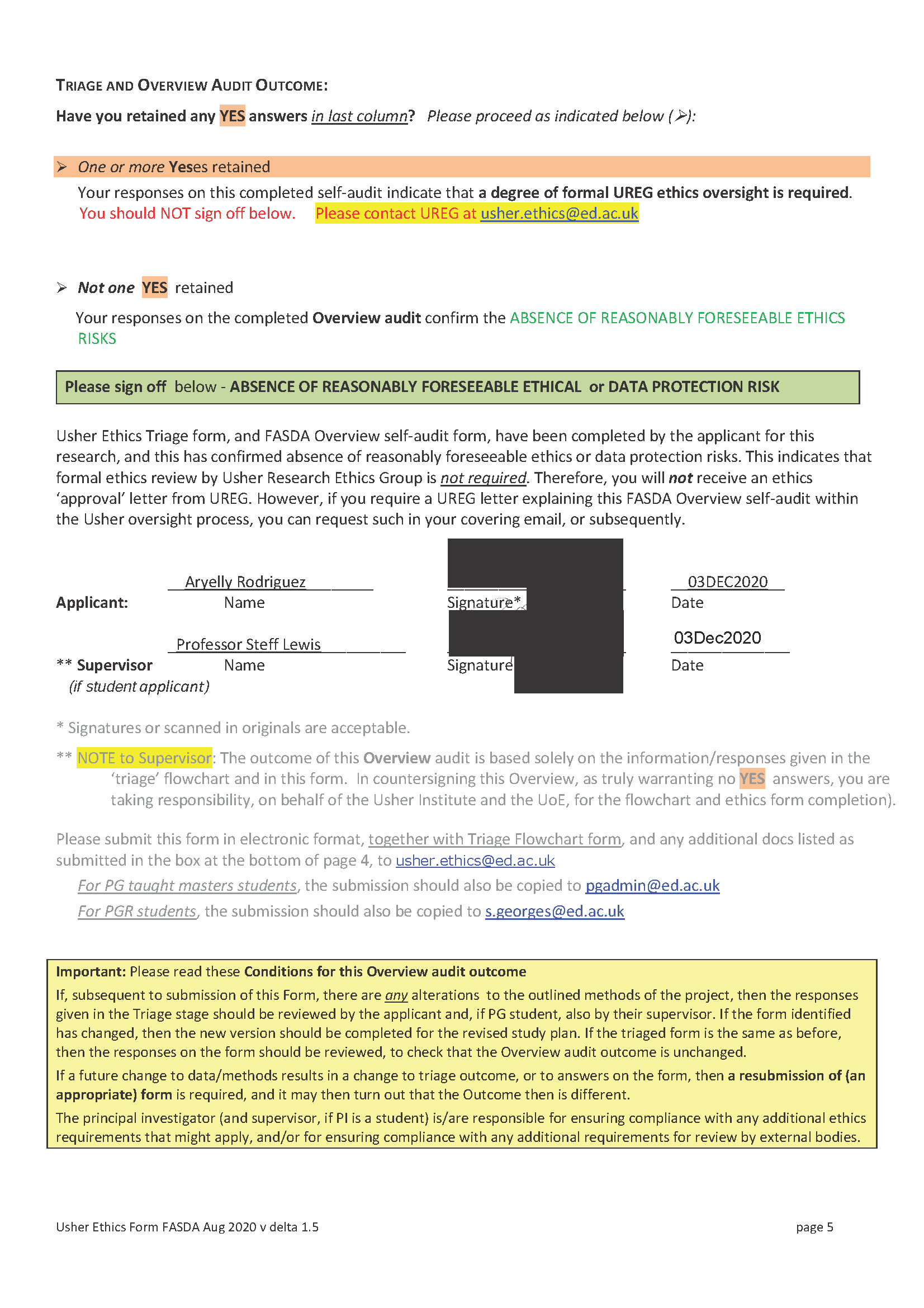

Supplement: sj-docx-2-ctj-10.1177_17407745251356423 – Supplemental material for Evaluating re-identification risks scores in publicly available clinical trial datasets: Insights and implications [file sj-docx-2-ctj-10.1177_17407745251356423.docx]
