# Supplementary material for: Evaluating re-identification risks scores in publicly available clinical trial datasets: Insights and implications
Source: Clin Trials. 2025 Aug 22;22(6):649–66. doi: 10.1177/17407745251356423 (PMC12647387; doi:10.1177/17407745251356423)
Supplement: sj-docx-3-ctj-10.1177_17407745251356423 – Supplemental material for Evaluating re-identification risks scores in publicly available clinical trial datasets: Insights and implications [file sj-docx-3-ctj-10.1177_17407745251356423.docx]

Appendix 6 Additional figures and tables

| Figure S6.1.1 | | Flow chart for the selection and analysis for clinical trials’ datasets |
| --- | --- | --- |
| 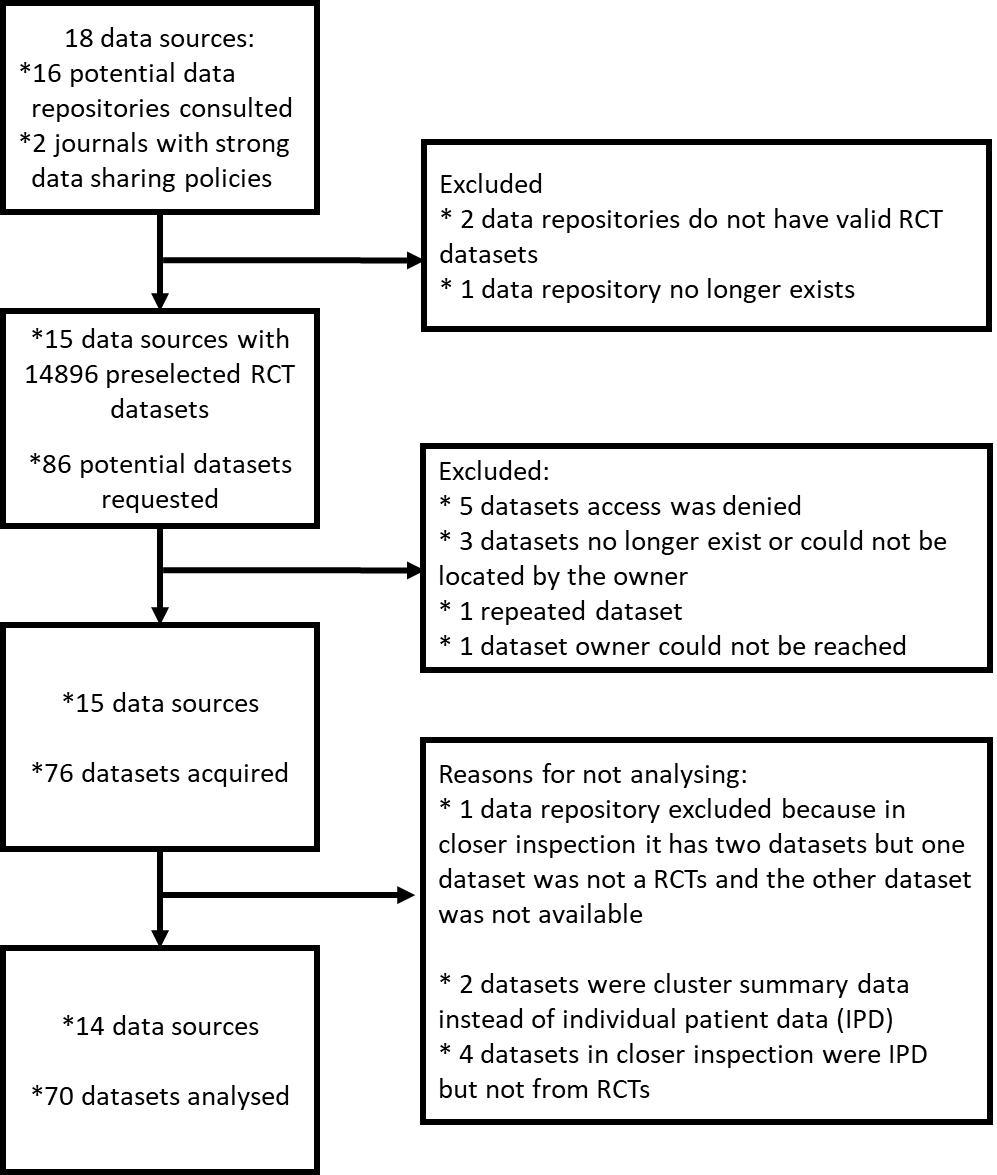 | | |
| Where: | RCT Randomised controlled trial  IPD Individual patient data | |

| Figure. S6.1.2 Mean re-identification Risks scores | | |  |
| --- | --- | --- | --- |
|  | | Prosecutor scenario | Journalist scenario |
| Mean with 95% CI | | 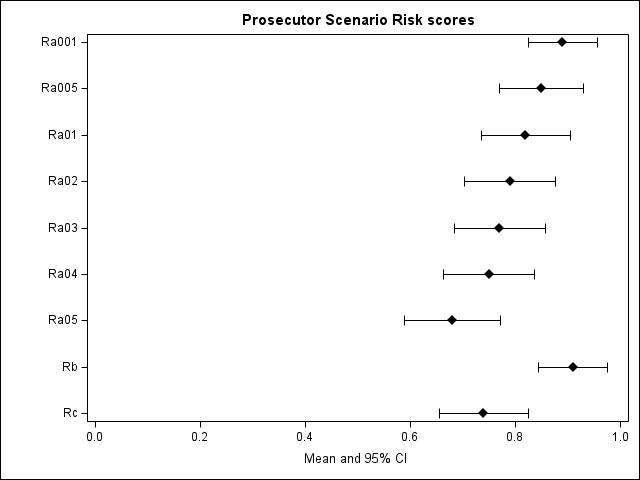 | 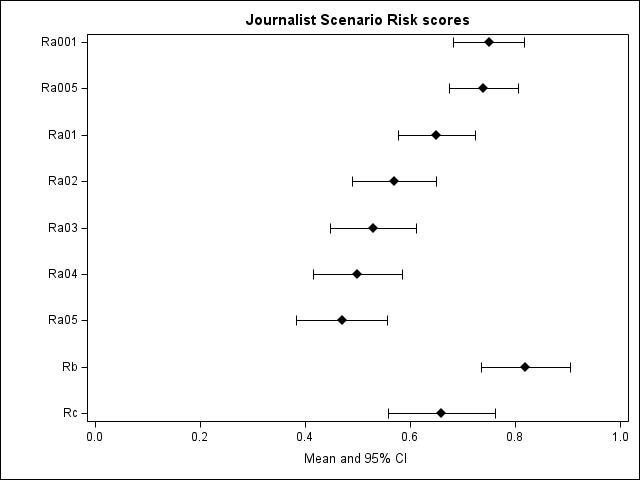 |
| By number of identifiers in clinical trial datasets | | 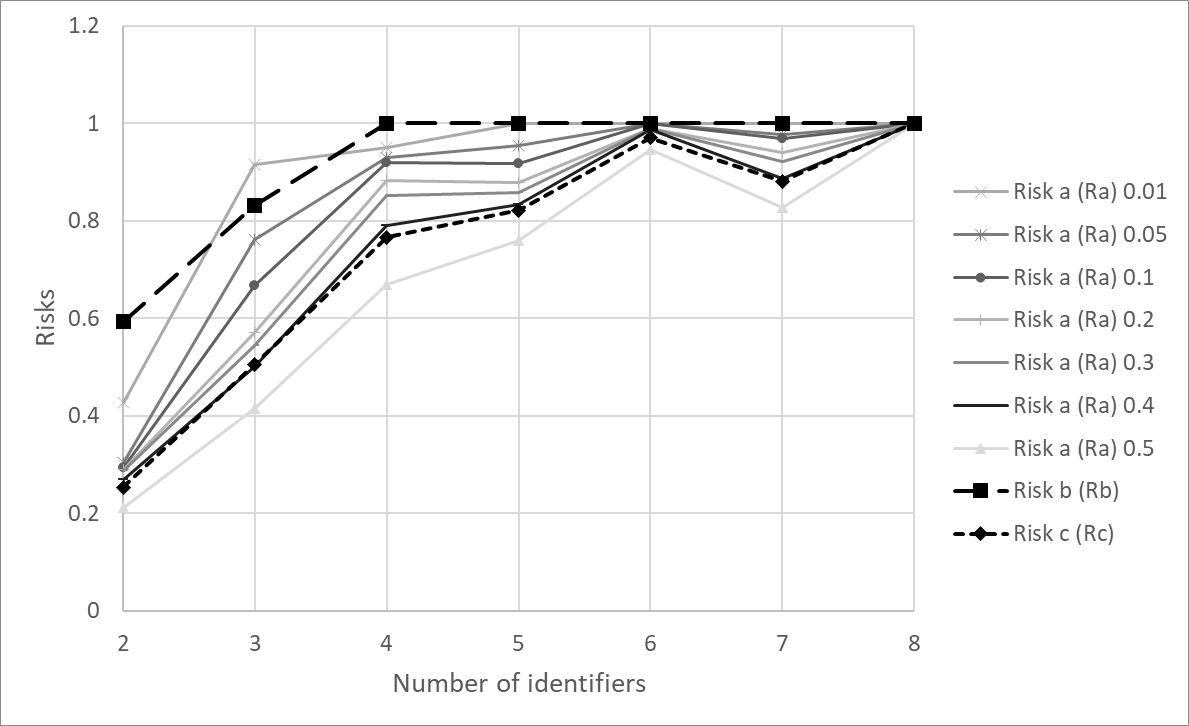  Note: automatic calculations of risk scores are not included in this graphs | 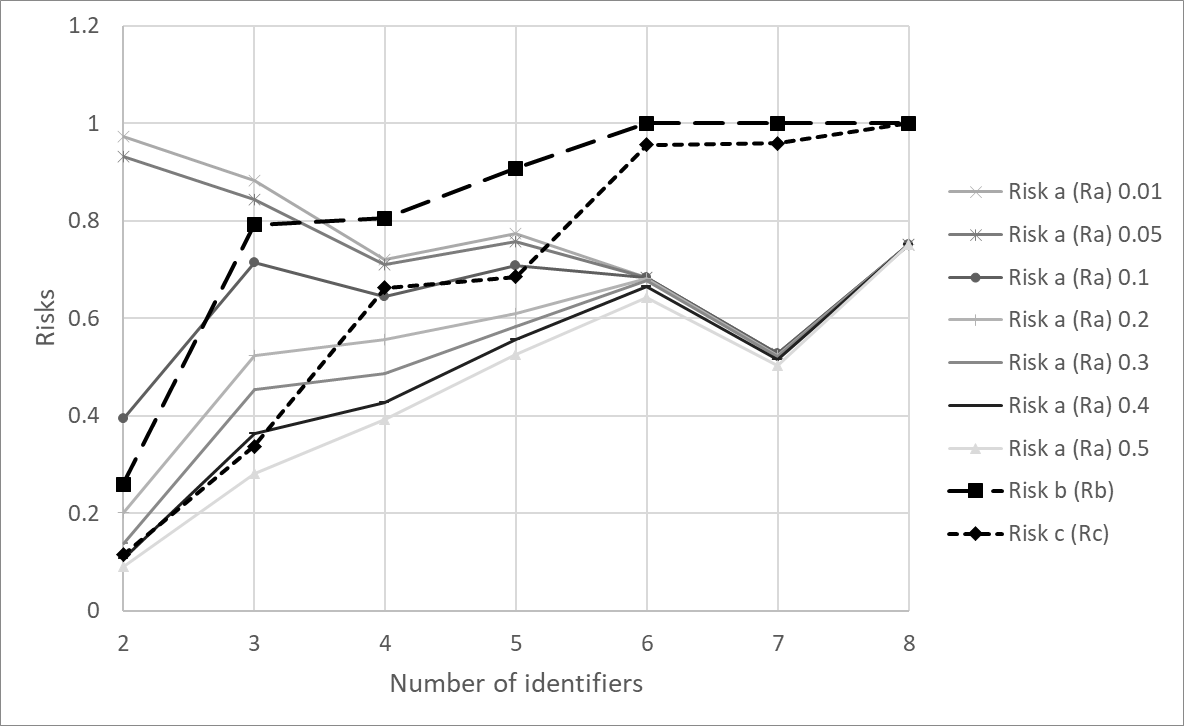  Note: automatic calculations of risk scores are not included in this graphs |
| Risks a (Ra) vs levels of threshold by scenario | | 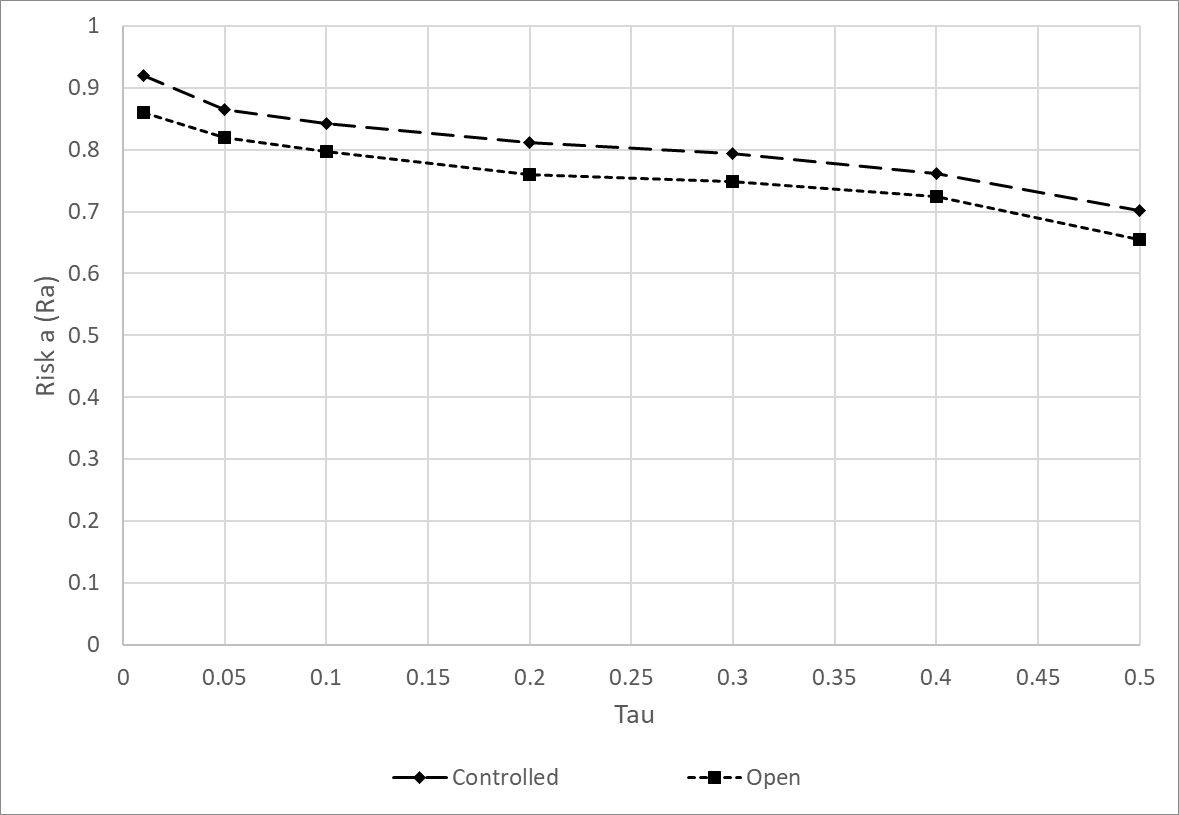 | 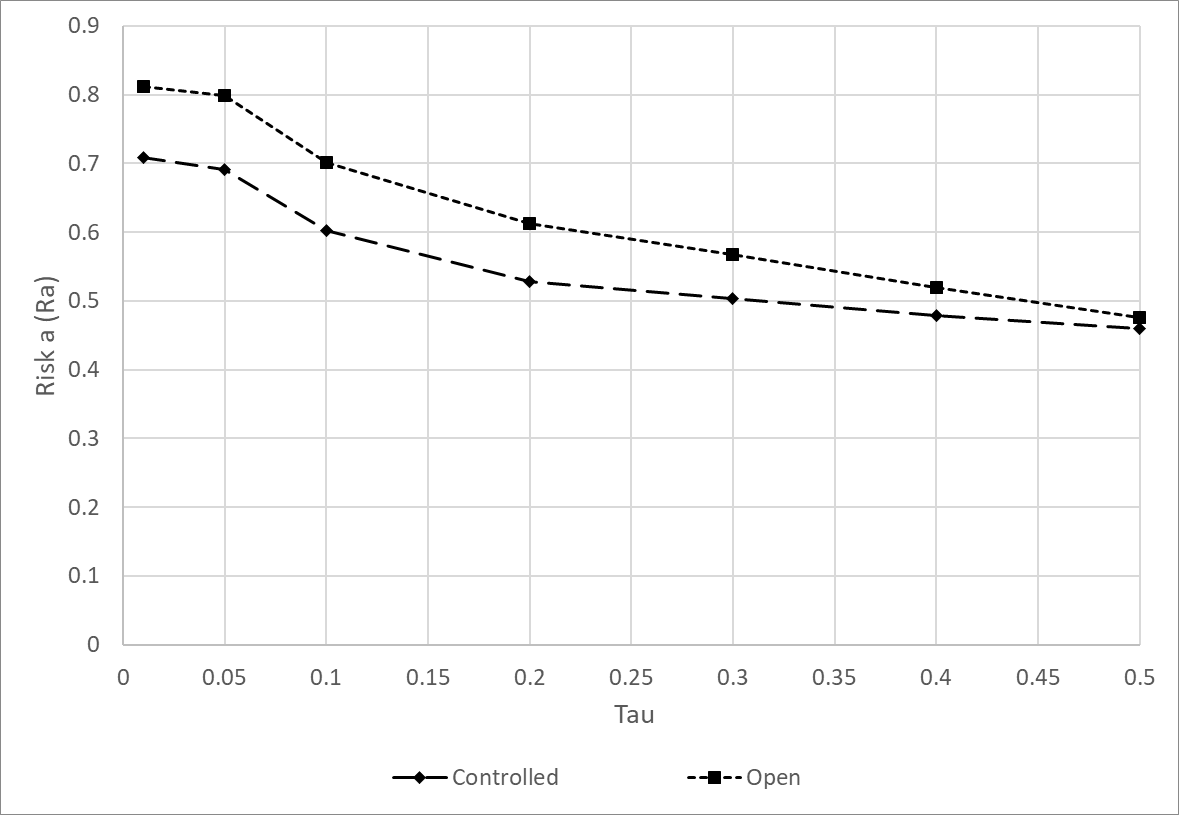 |
| Where: | Risk a (Ra): the proportions of participants in strata above a predetermined risk threshold, Risk b (Rb): the stratum with the smallest membership in the anonymised dataset, and Risk c (Rc): the average risk score across the whole strata of the anonymised dataset, using all indirect identifiers, calculated with the formulas in chapter 16 from “Guide to the de-identification of personal health information” by Khaled El Emam (2013) | | |

| Index of Pre-planned Plots for Re-identification Risk Scores in clinical trials’ datasets | | |
| --- | --- | --- |
| Figure S6.2.1 | | Scatterplots of Ra vs anonymised clinical trial datasets’ sample size |
| Figure S6.2.2 | | Box-and-whisker plots of Ra vs number of indirect identifiers |
| Figure S6.2.3 | | Scatterplot of Rb vs anonymised clinical trial datasets’ sample size |
| Figure S6.2.4 | | Box-and-whisker plots of Rb vs number of indirect identifiers |
| Figure S6.2.5 | | Scatterplot of Rc vs anonymised clinical trial datasets’ sample size |
| Figure S6.2.6 | | Box-and-whisker plots of Rc vs number of indirect identifiers |
| Figure S6.2.7 | | Scatterplots of Ra vs Rb |
| Figure S6.2.8 | | Scatterplots of Ra vs Rc |
| Figure S6.2.9 | | Scatterplots of Rb vs Rc |
| Where: | Risk a (Ra): the proportions of participants in strata above a predetermined risk threshold, Risk b (Rb): the stratum with the smallest membership in the anonymised dataset, and Risk c (Rc): the average risk score across the whole strata of the anonymised dataset, using all indirect identifiers, calculated with the formulas in chapter 16 from “Guide to the de-identification of personal health information” by Khaled El Emam (2013) | |

| Figure S6.2.1 | Scatterplots of Ra vs anonymised clinical trial datasets’ sample size | |
| --- | --- | --- |
| Prosecutor scenario | | Journalist scenario |
| 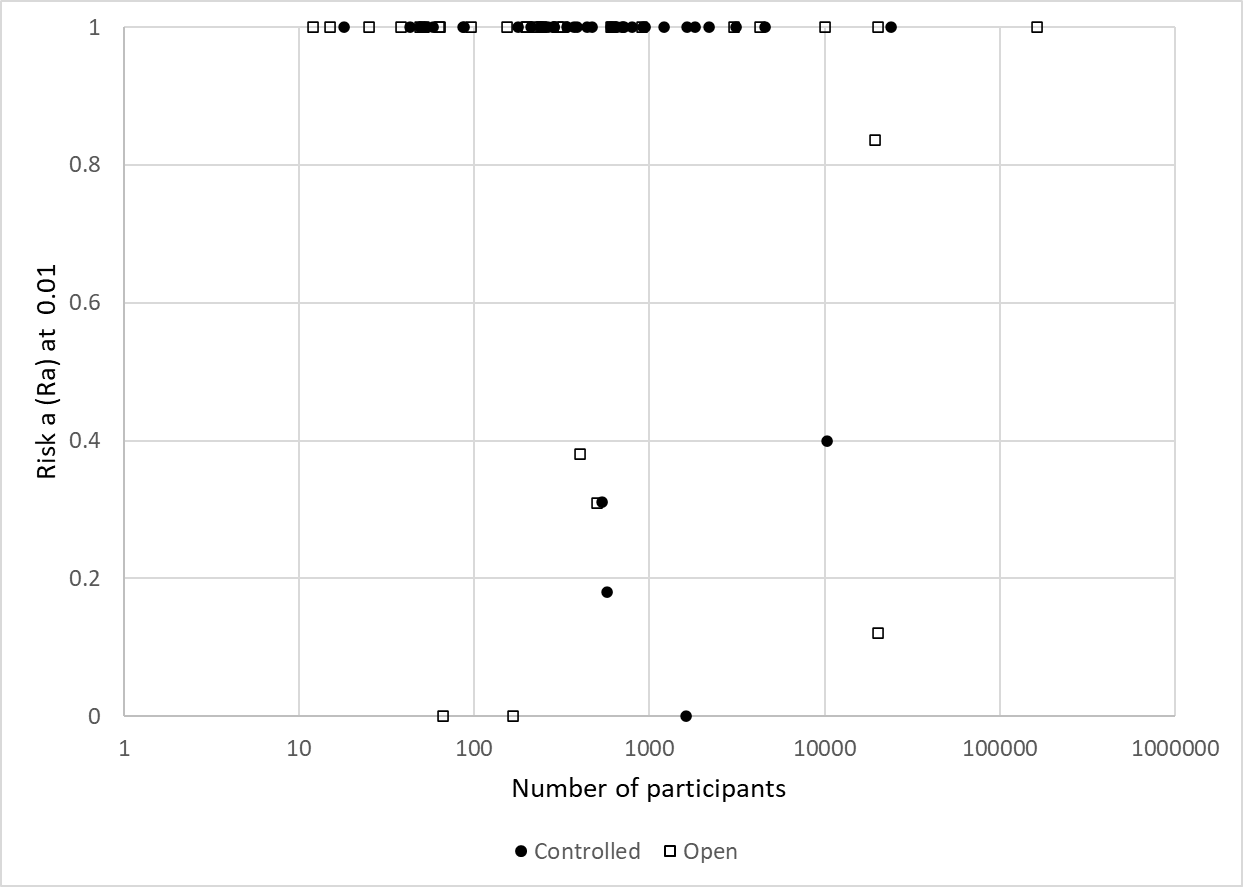 | | 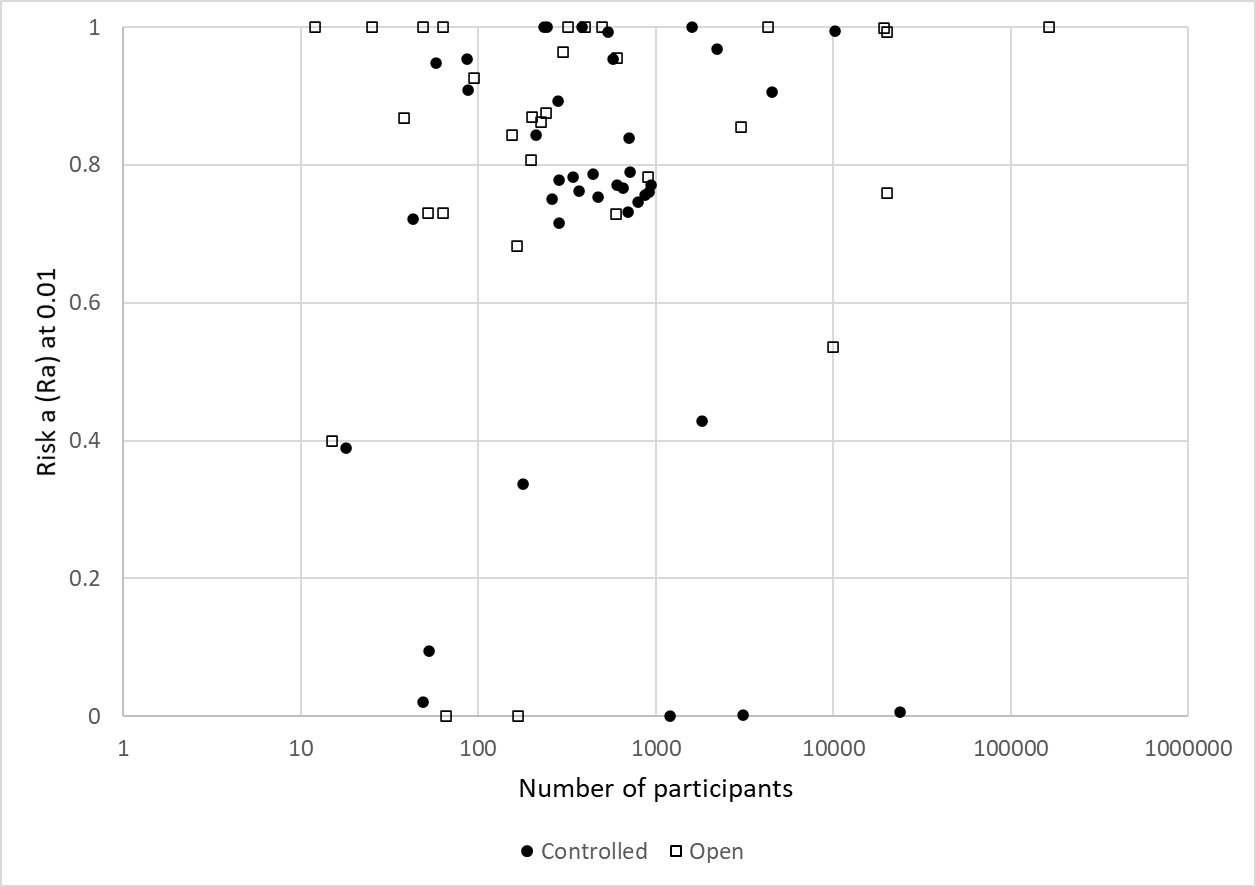 |
| 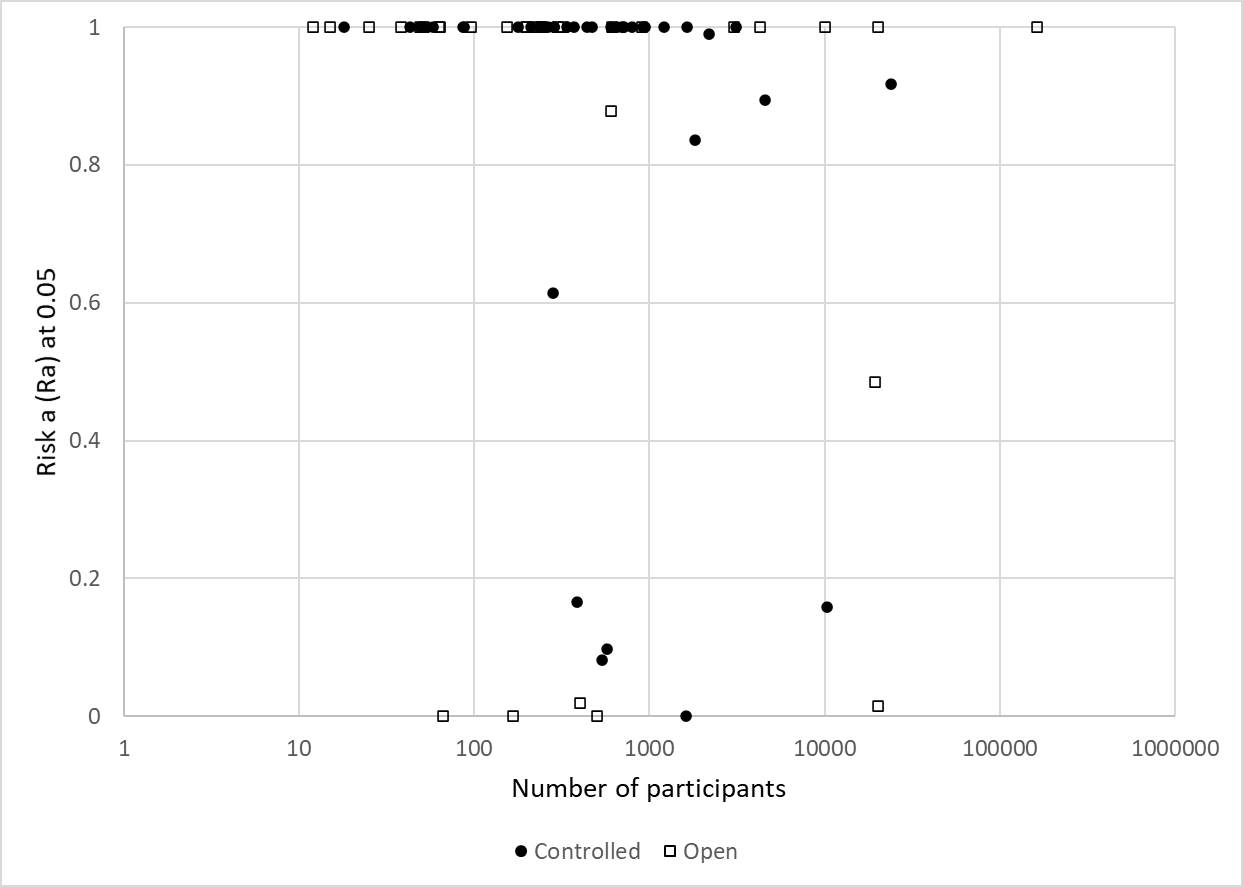 | | 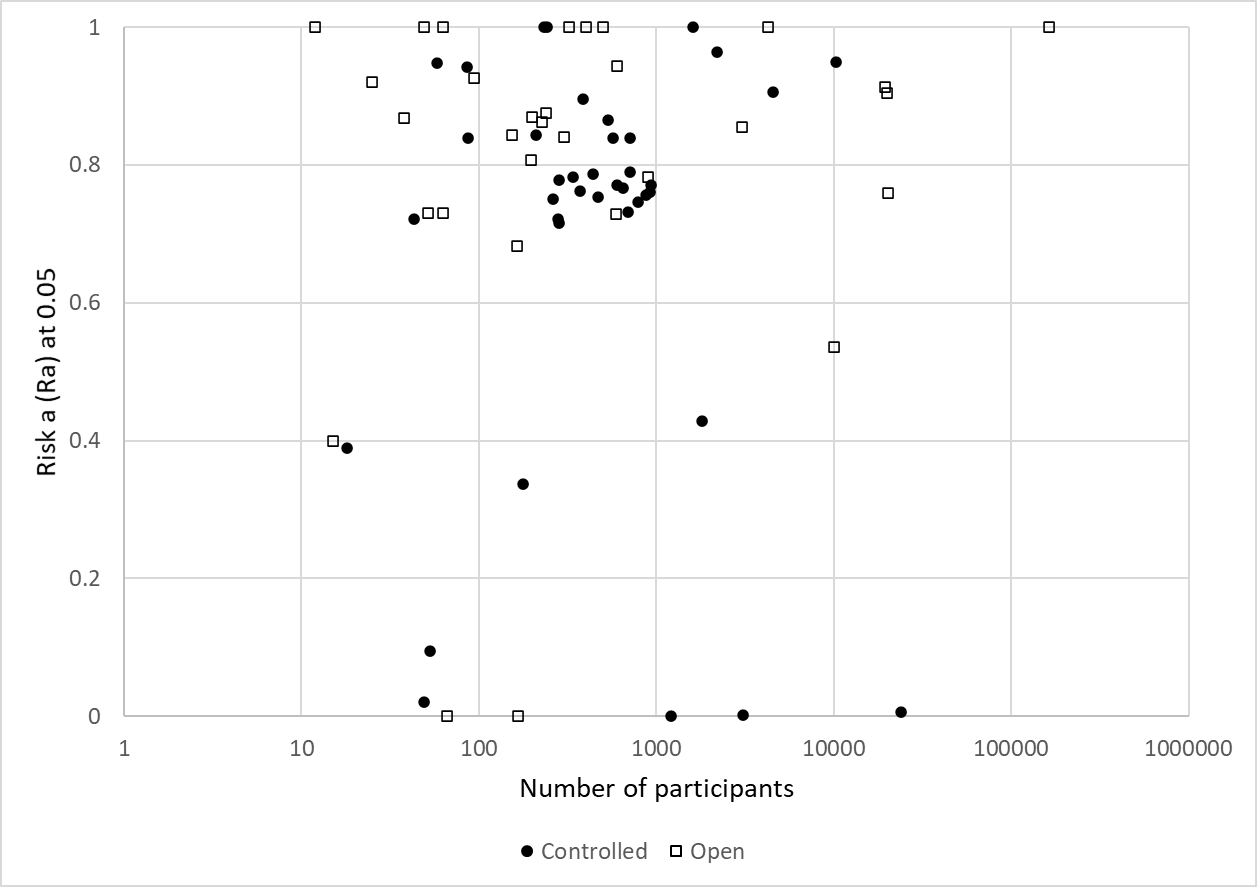 |
| 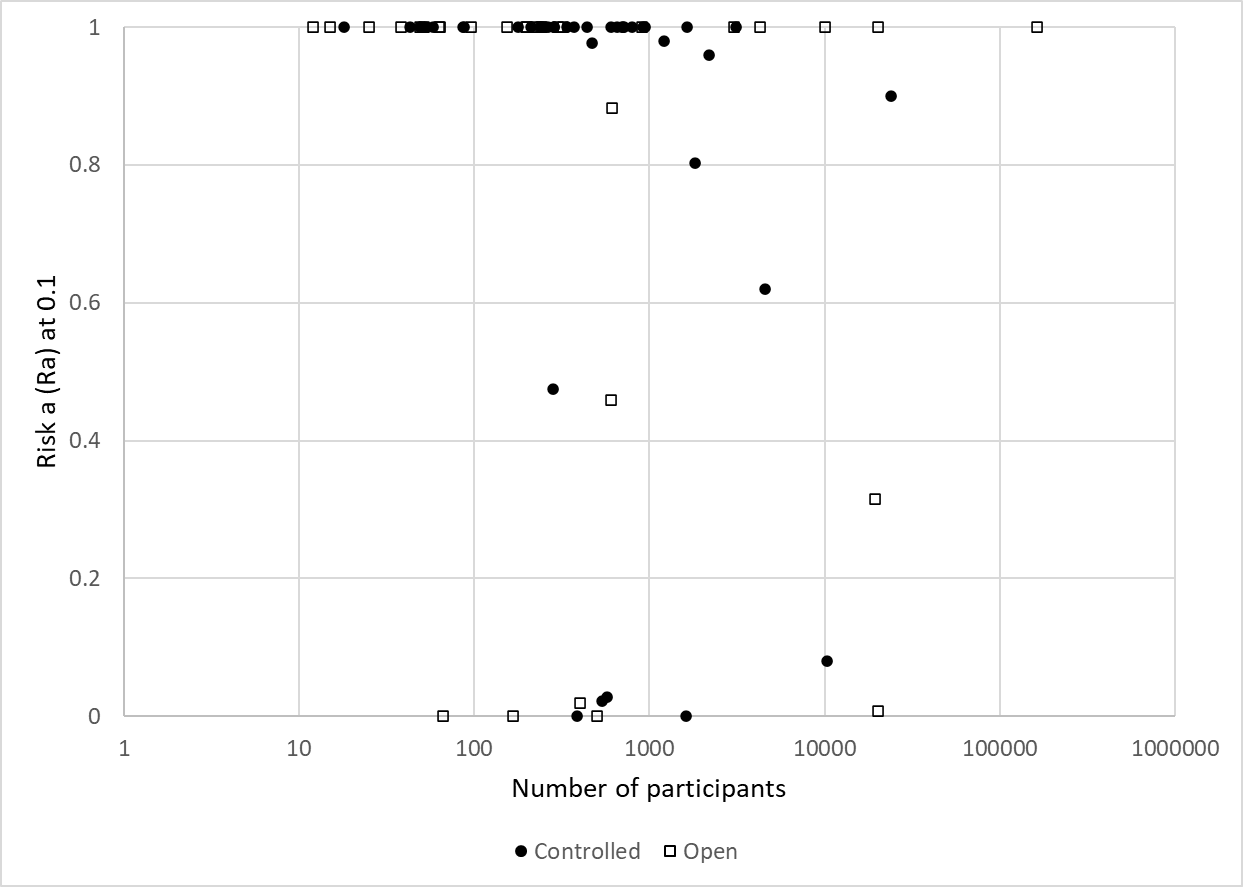 | | 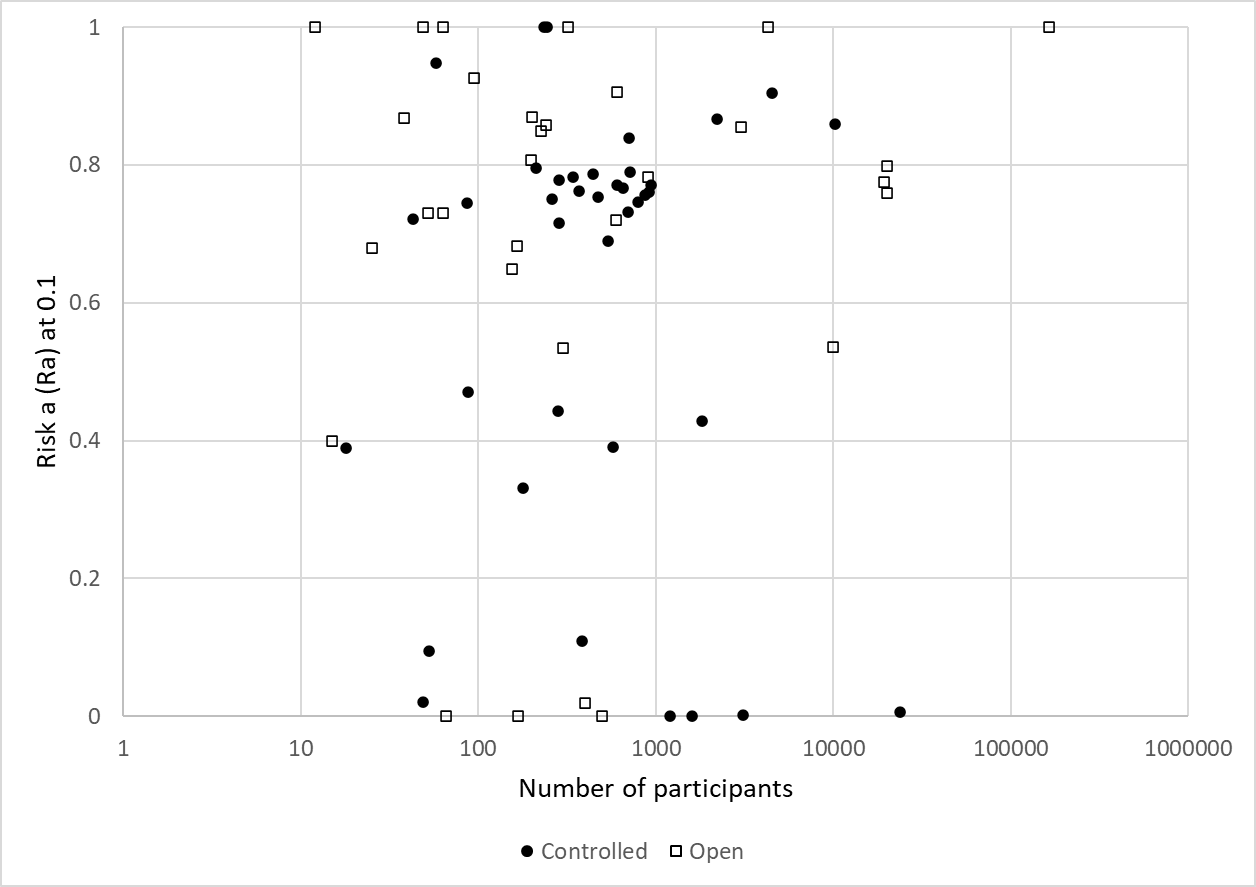 |
| 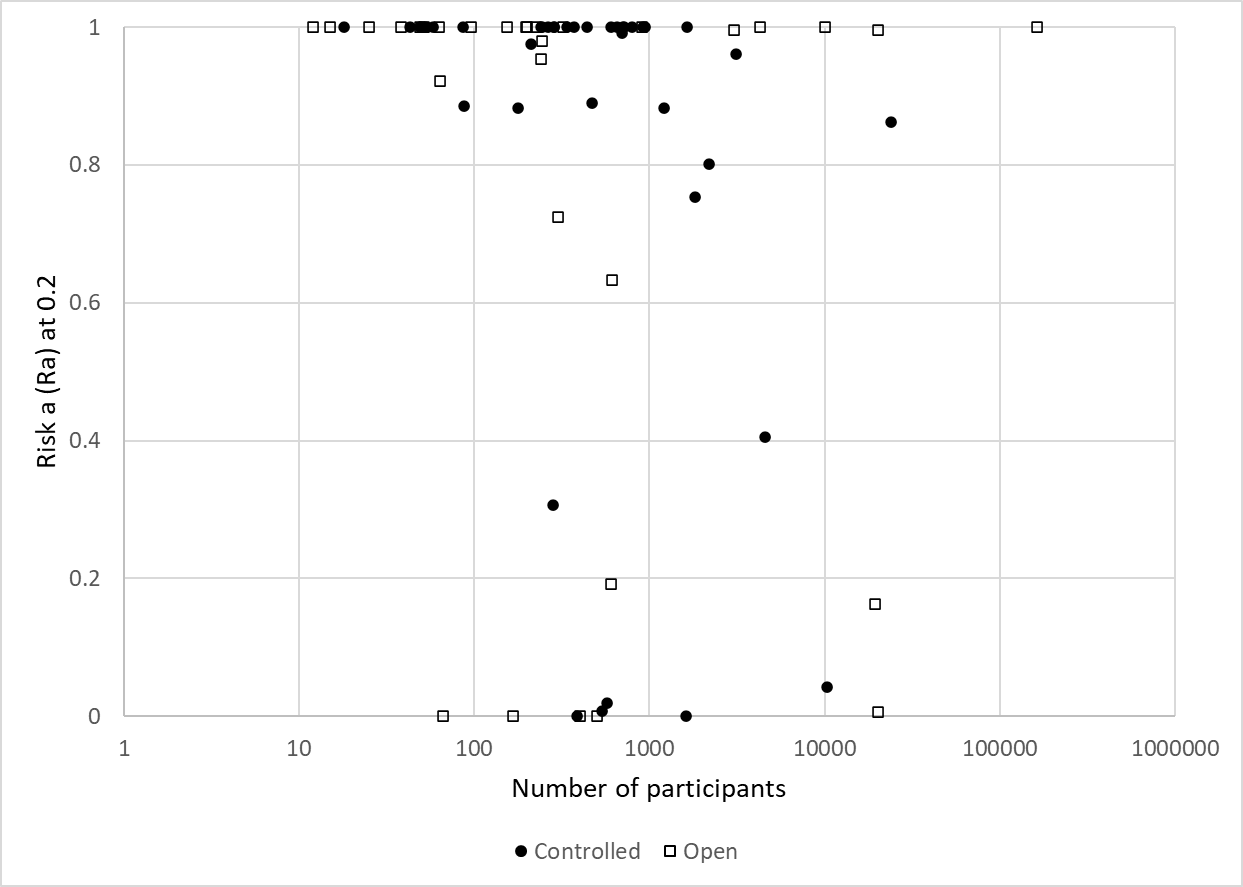 | | 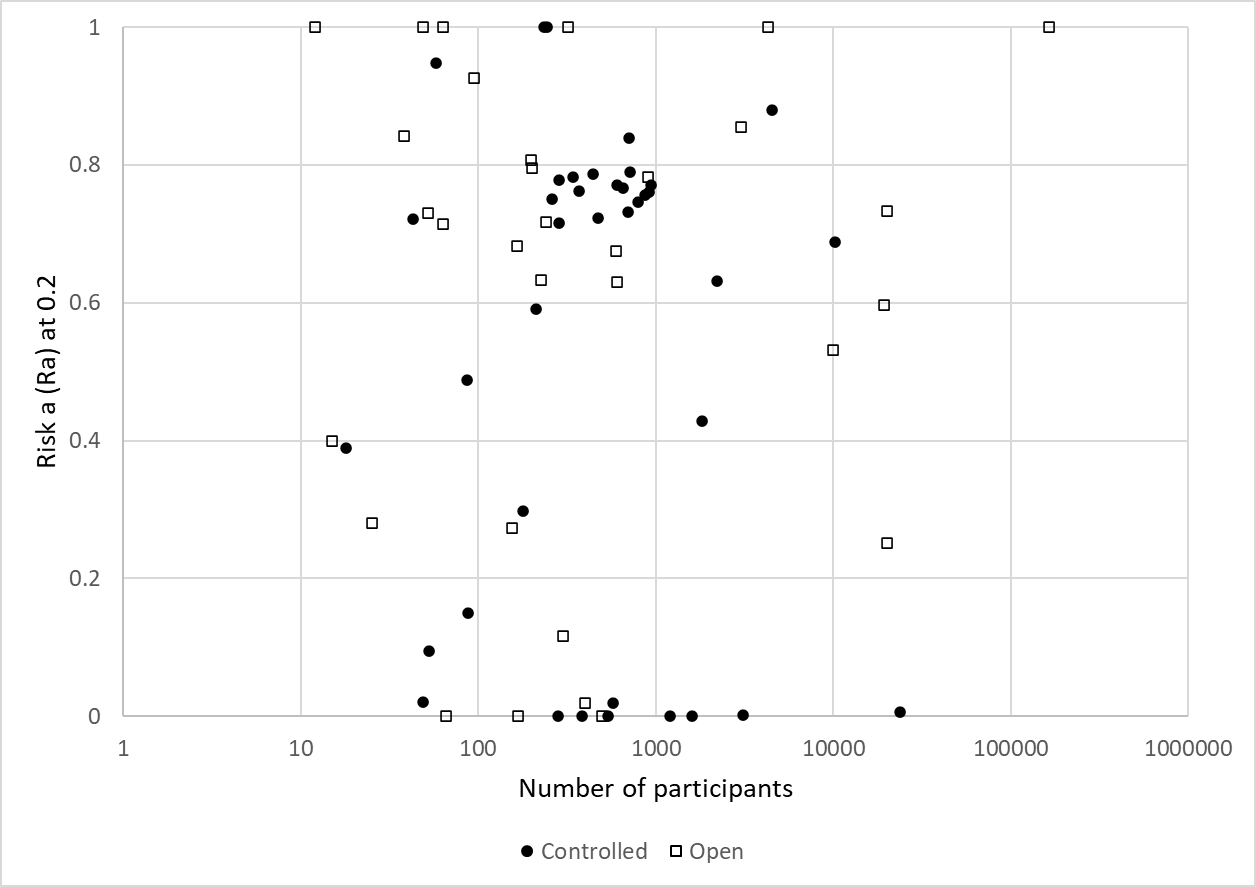 |
| 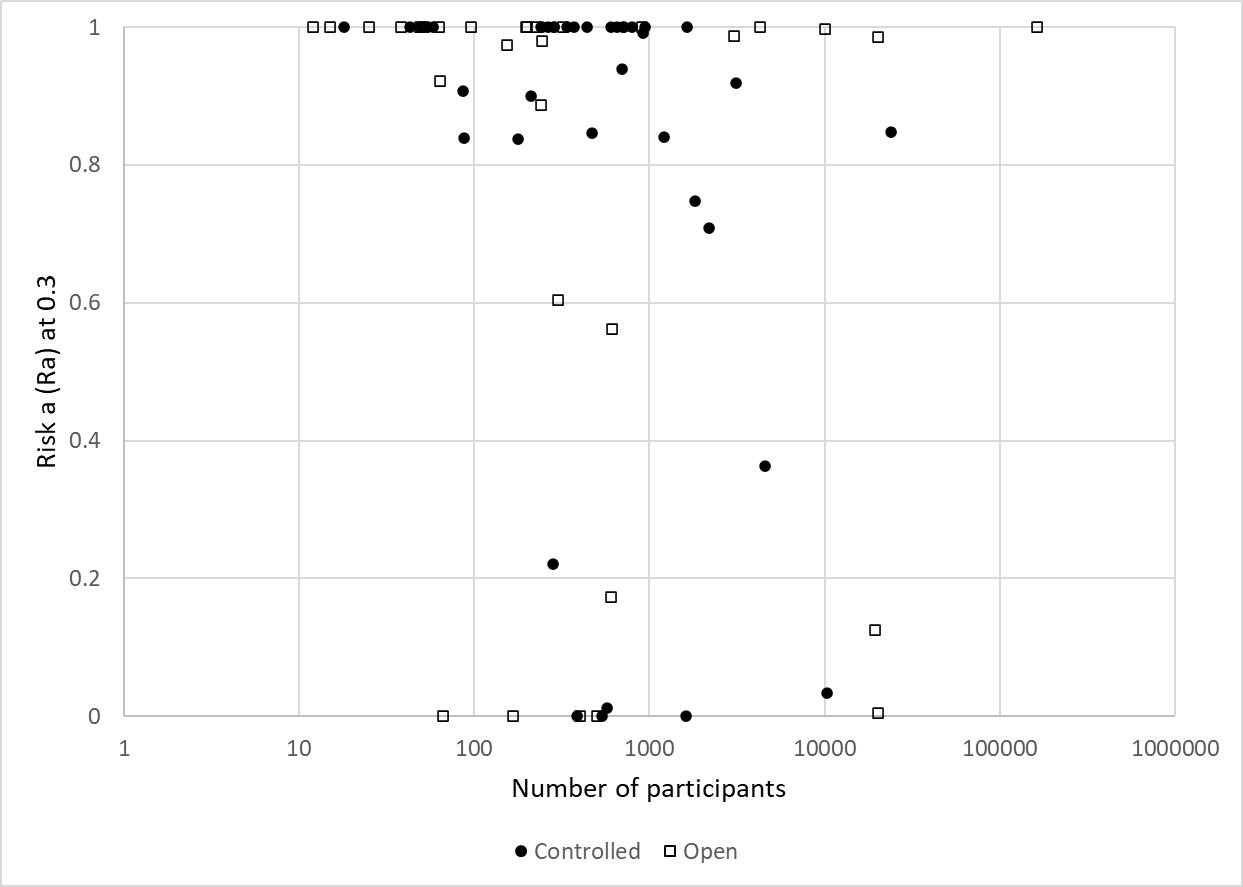 | | 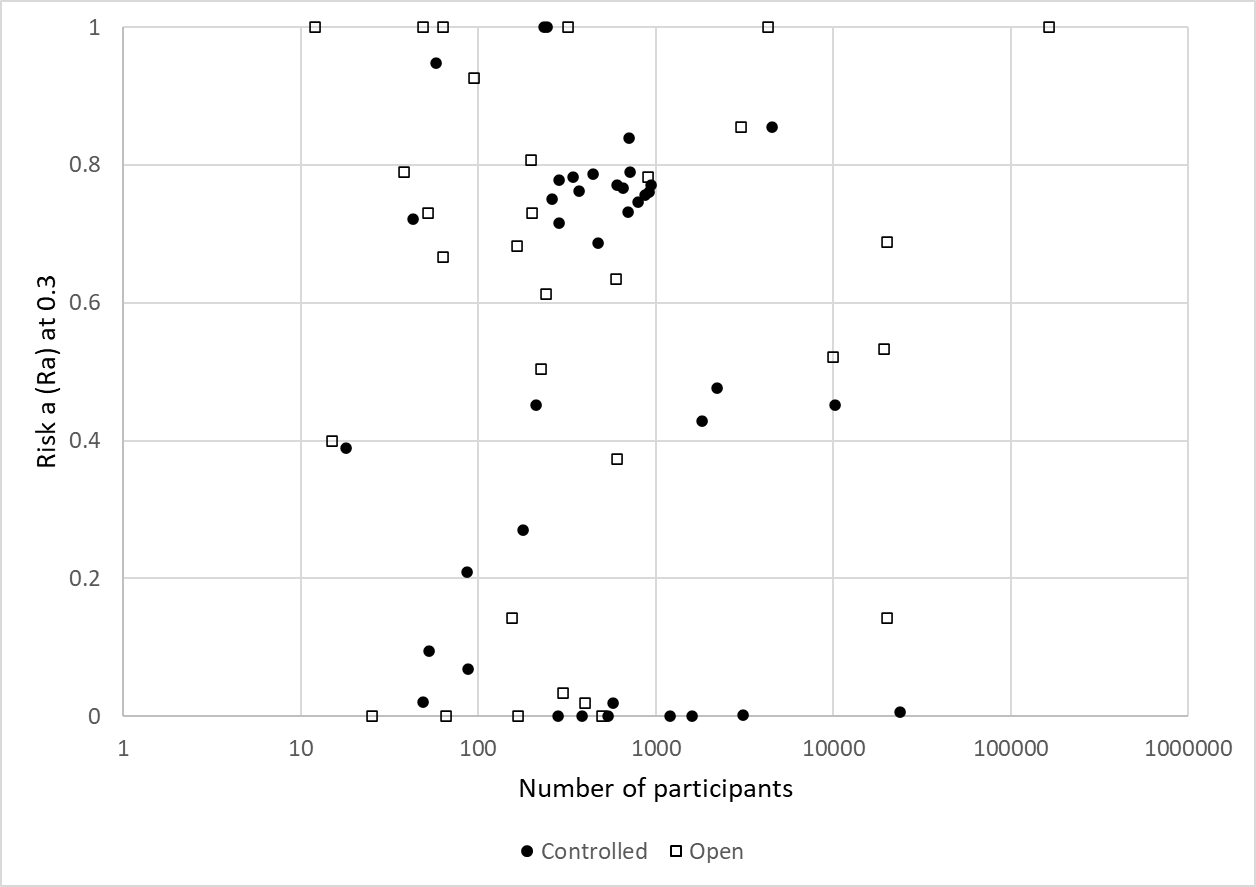 |
| 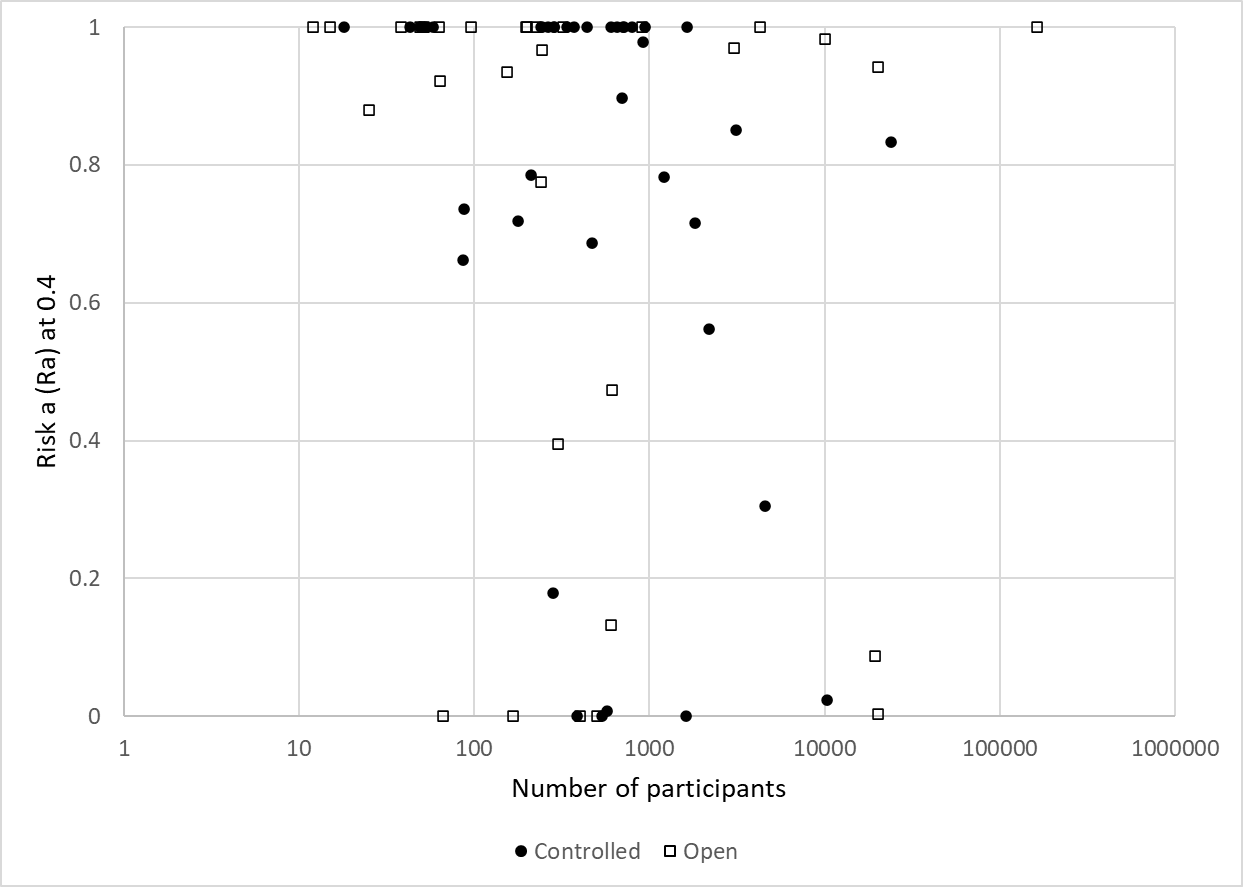 | | 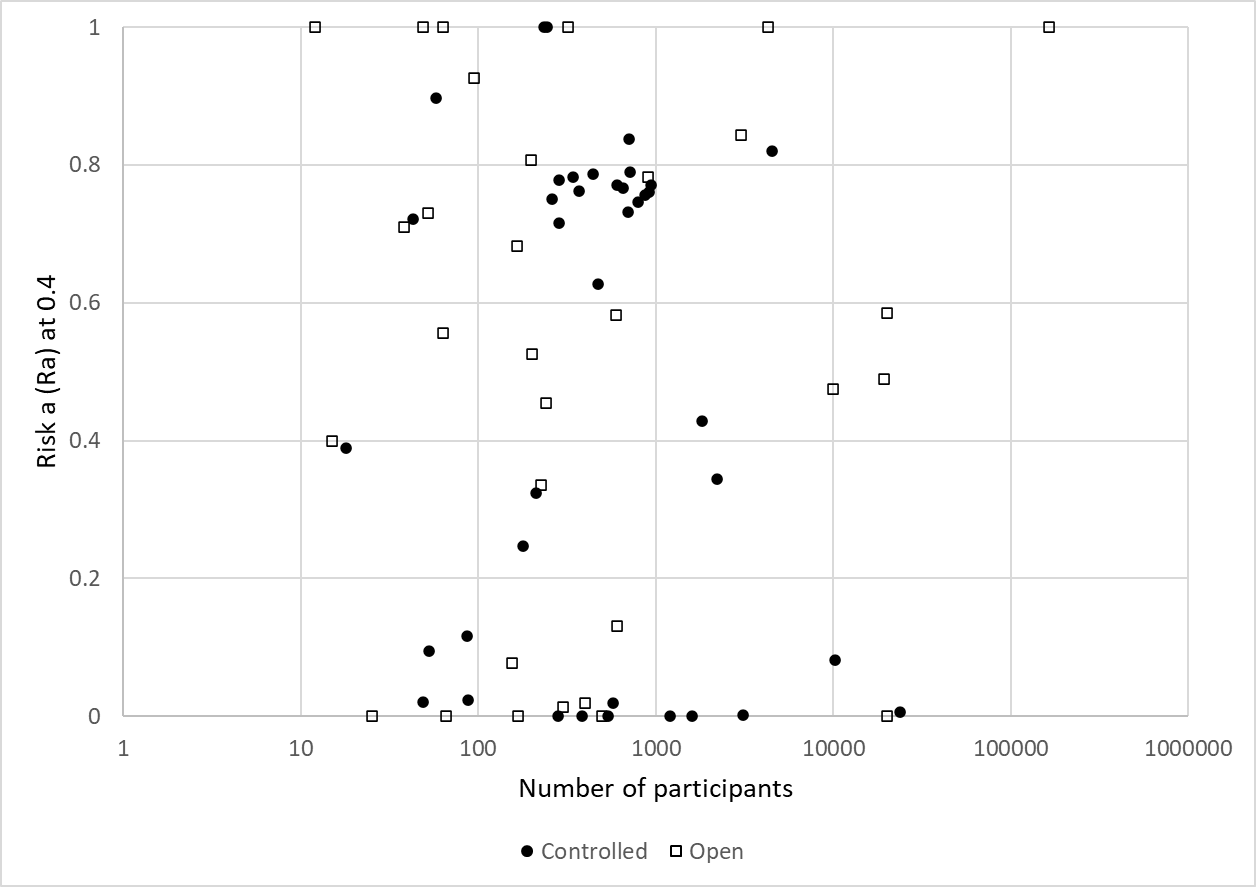 |
| 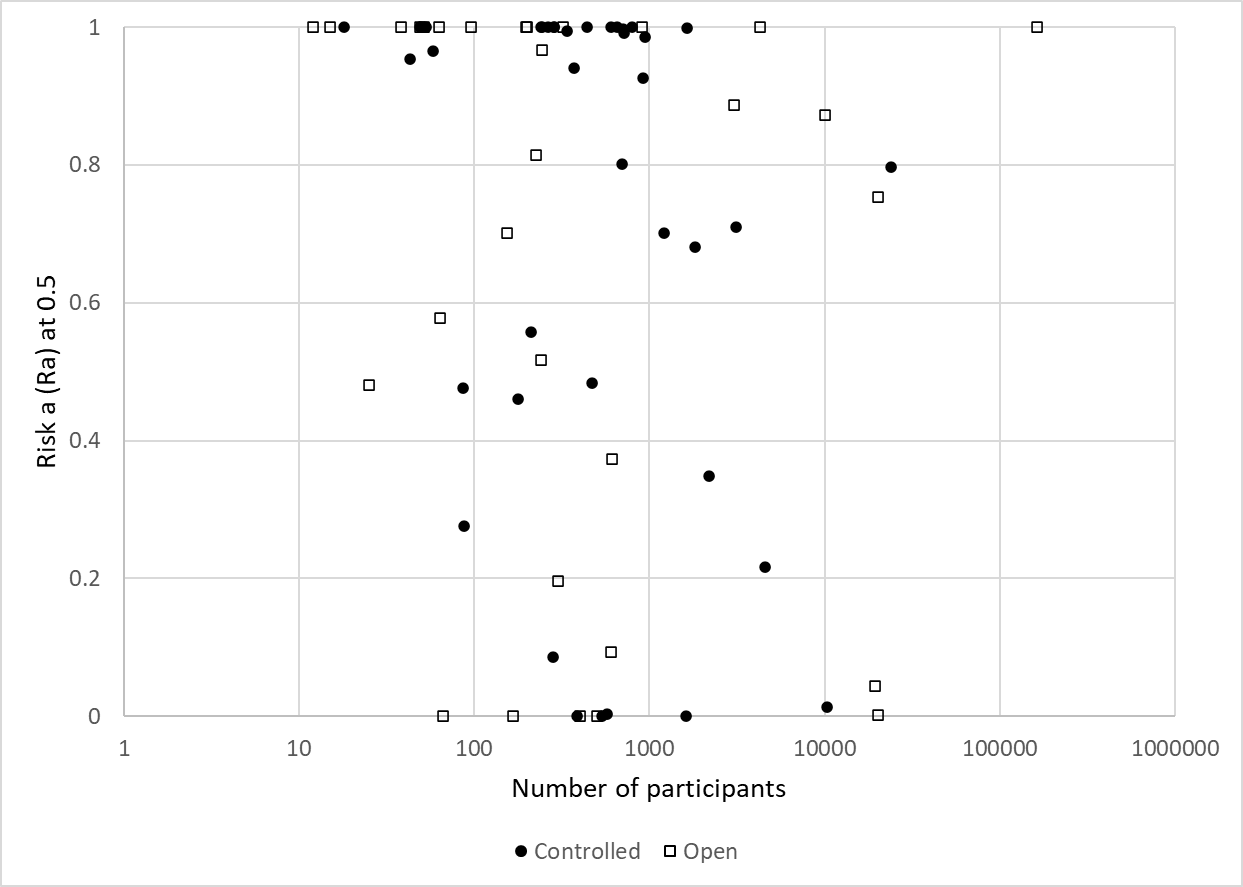 | | 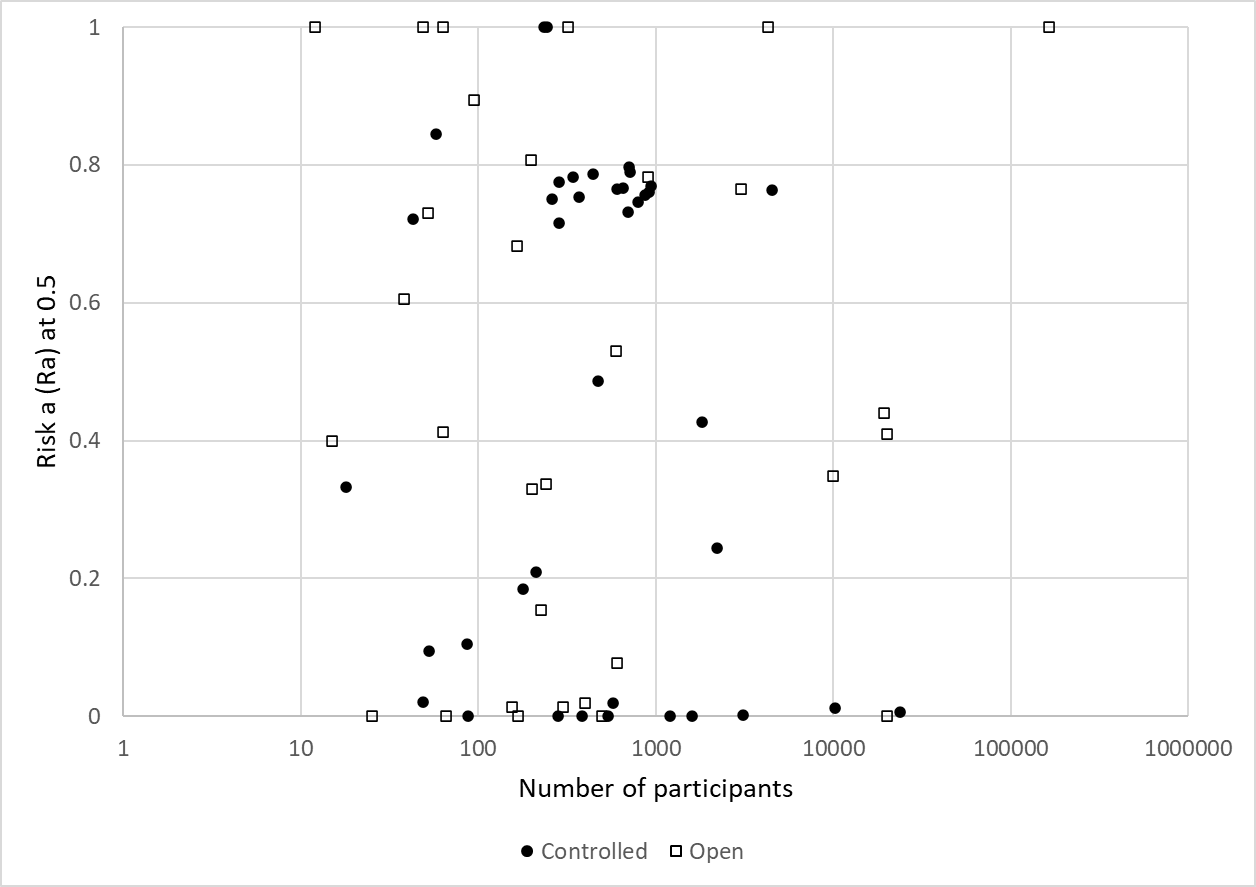 |

| Figure S6.2.2 | Box-and-whisker plots of Ra vs number of indirect identifiers | |
| --- | --- | --- |
| Prosecutor scenario | | Journalist scenario |
| 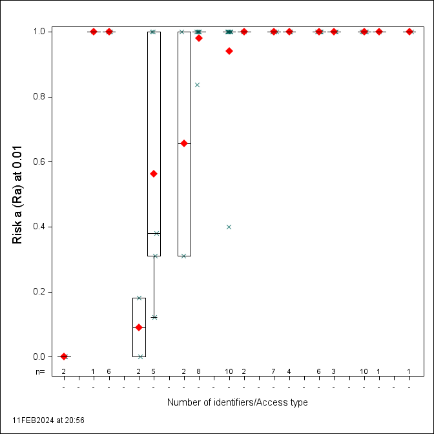 | | 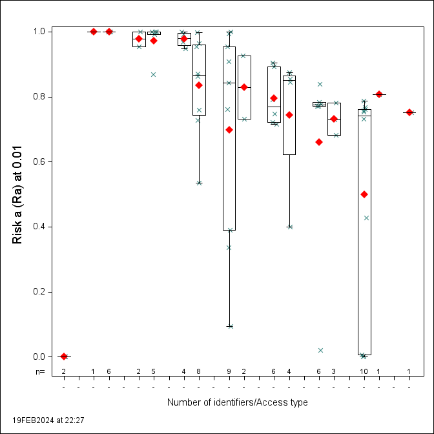 |
| 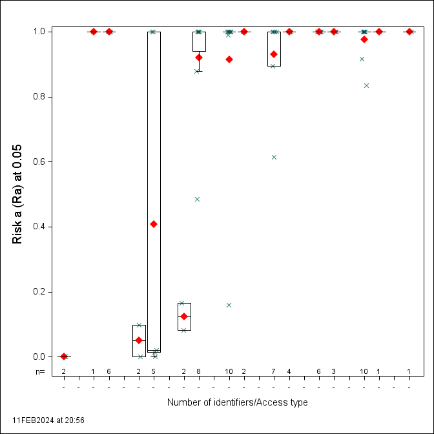 | | 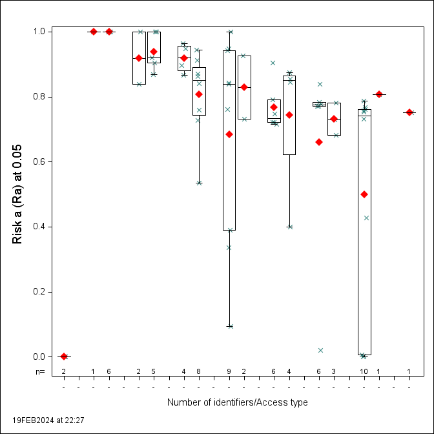 |
| 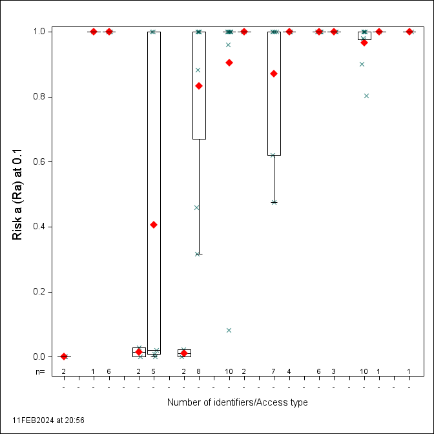 | | 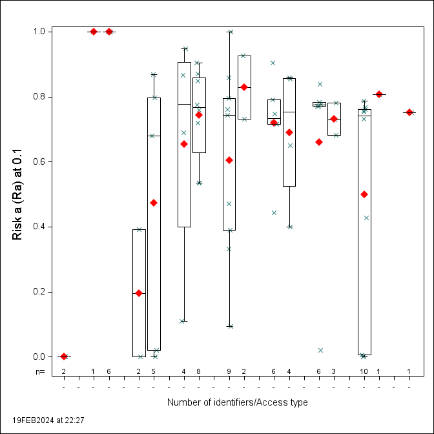 |
| 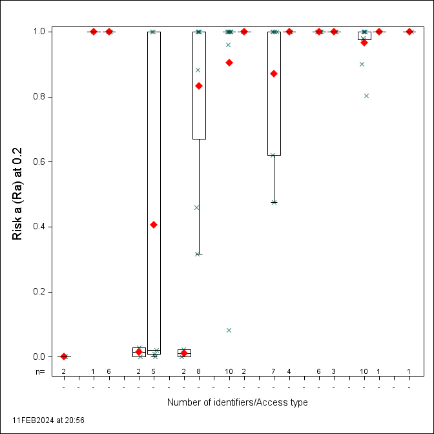 | | 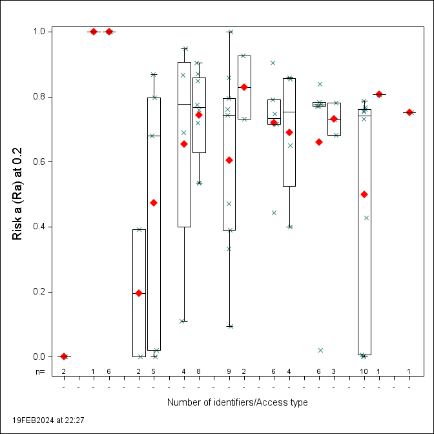 |
| 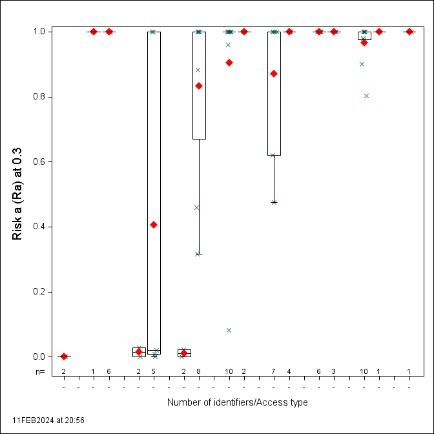 | | 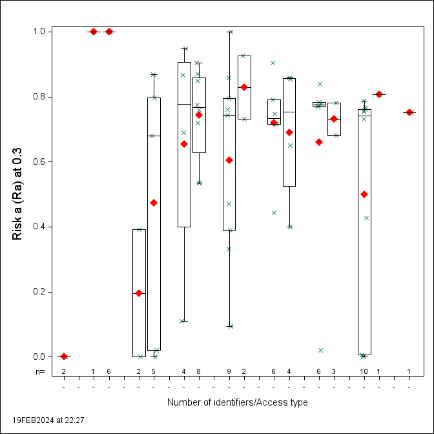 |
| 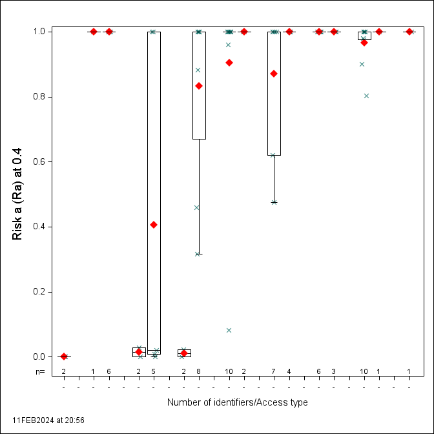 | | 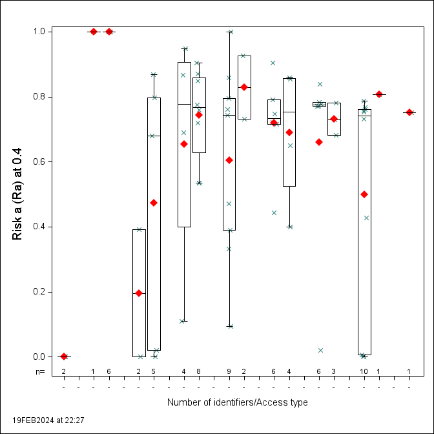 |
| 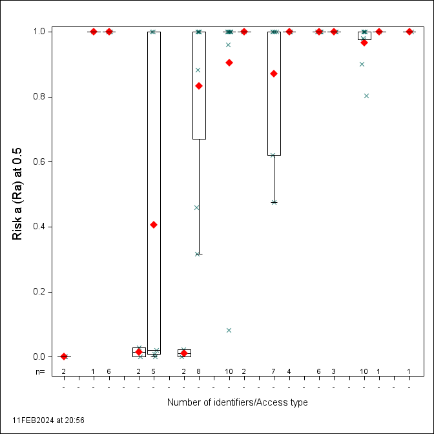 | | 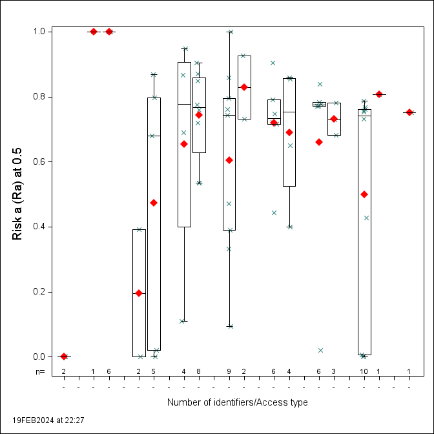 |

| Figure S6.2.3 | Scatterplots of Rb vs anonymised clinical trial datasets’ sample size | |
| --- | --- | --- |
| Prosecutor scenario | | Journalist scenario |
| 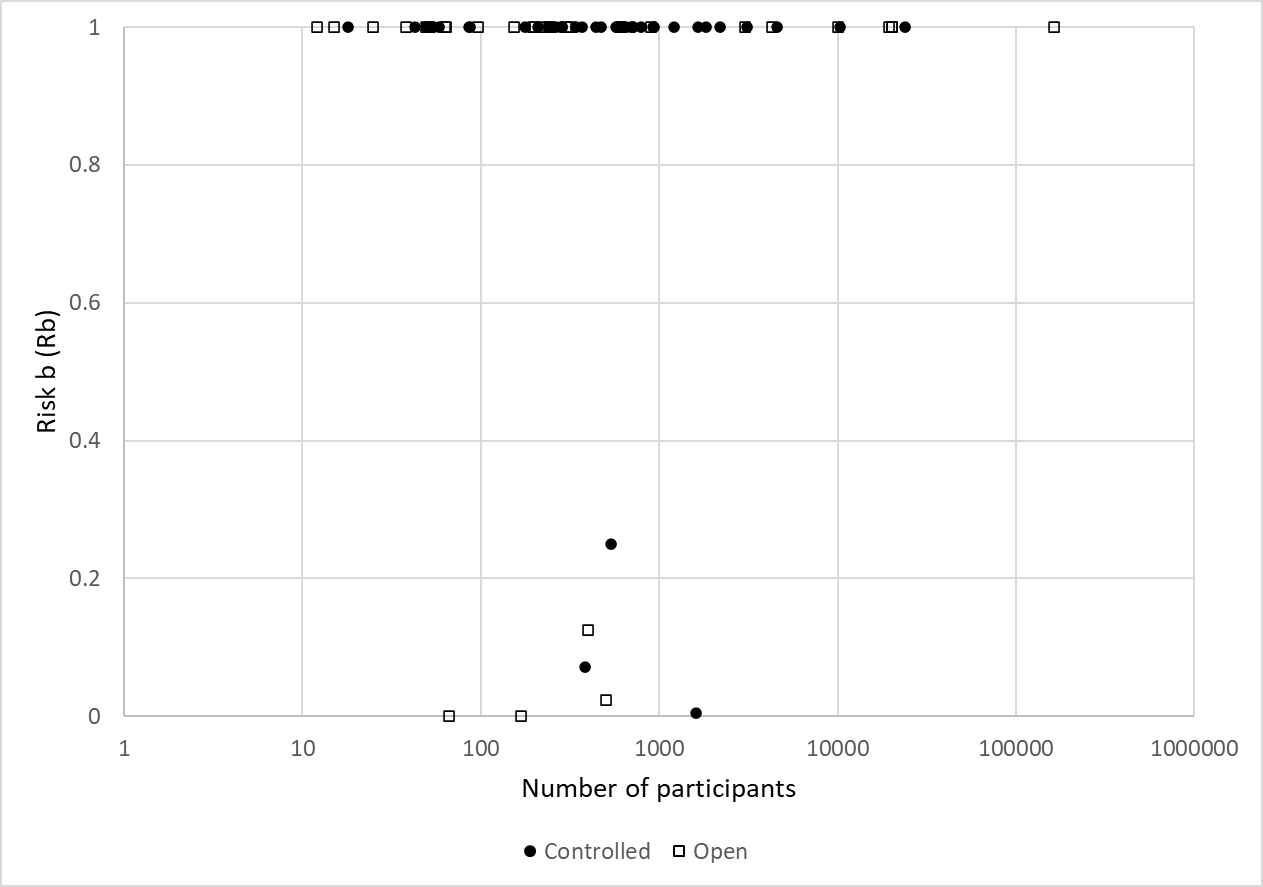 | | 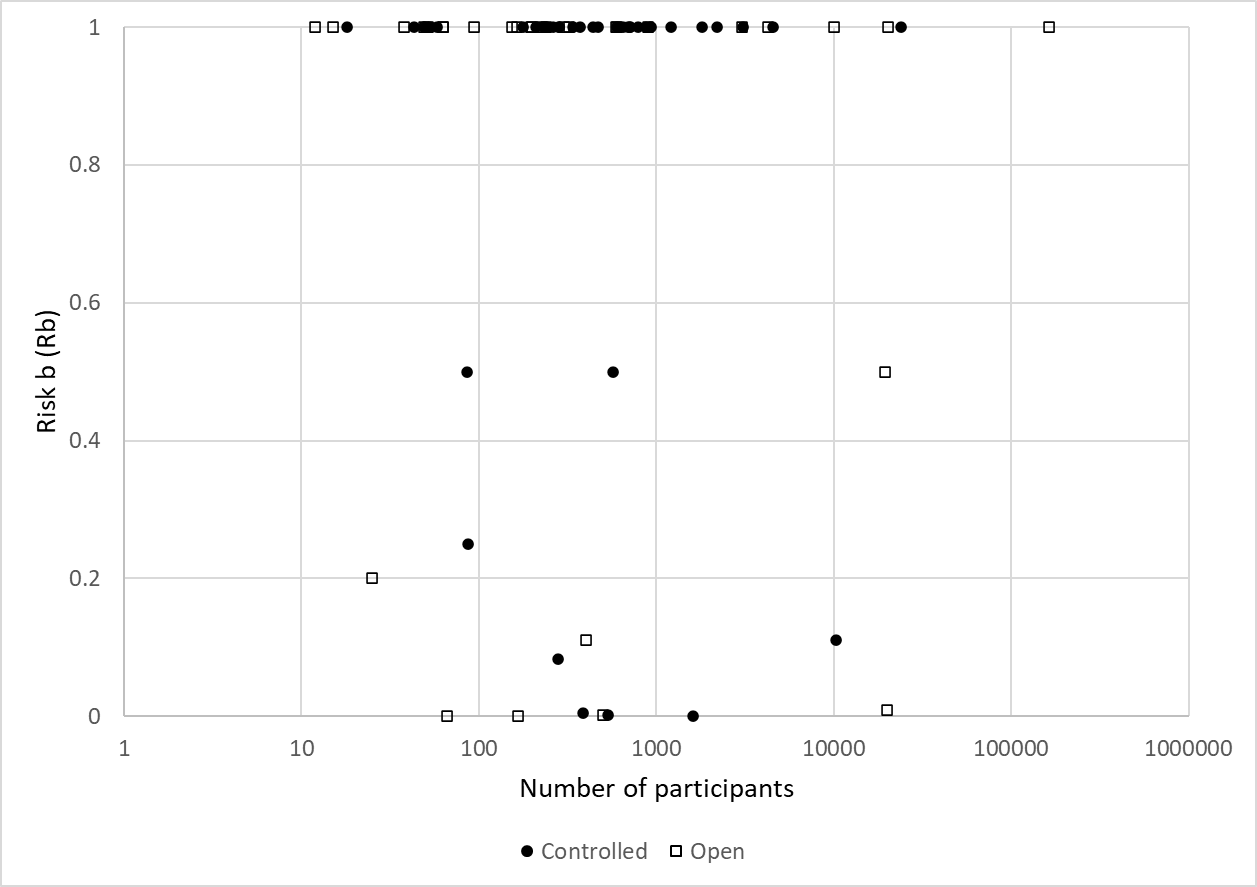 |

| Figure S6.2.4 | Box-and-whisker plots of Rb vs number of indirect identifiers | |
| --- | --- | --- |
| Prosecutor scenario | | Journalist scenario |
| 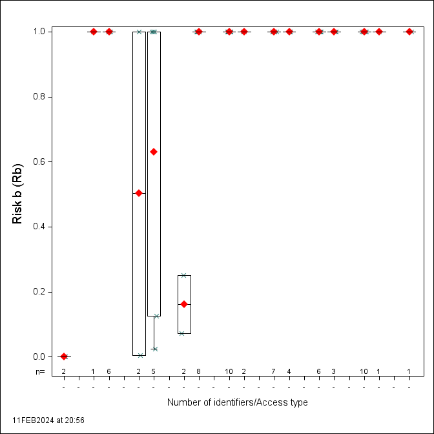 | | 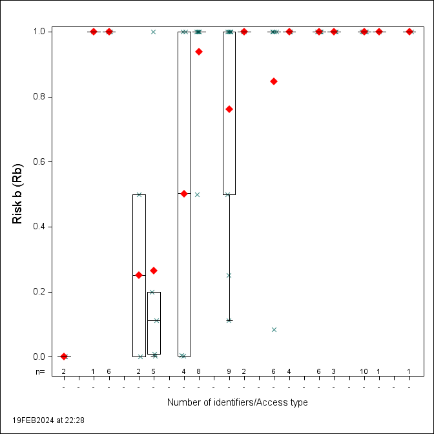 |

| Figure S6.2.5 | Scatterplots of Rc vs anonymised clinical trial datasets’ sample size | |
| --- | --- | --- |
| Prosecutor scenario | | Journalist scenario |
| 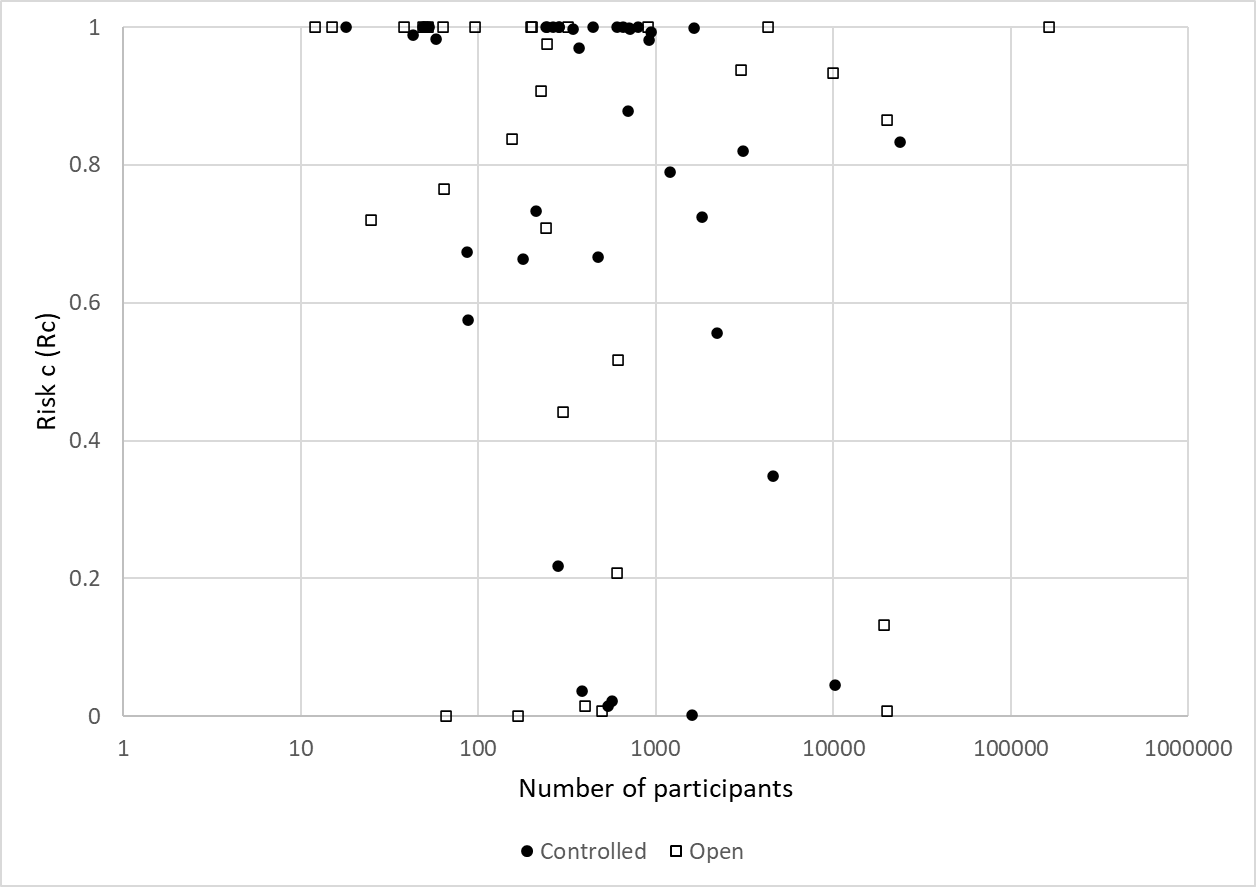 | | 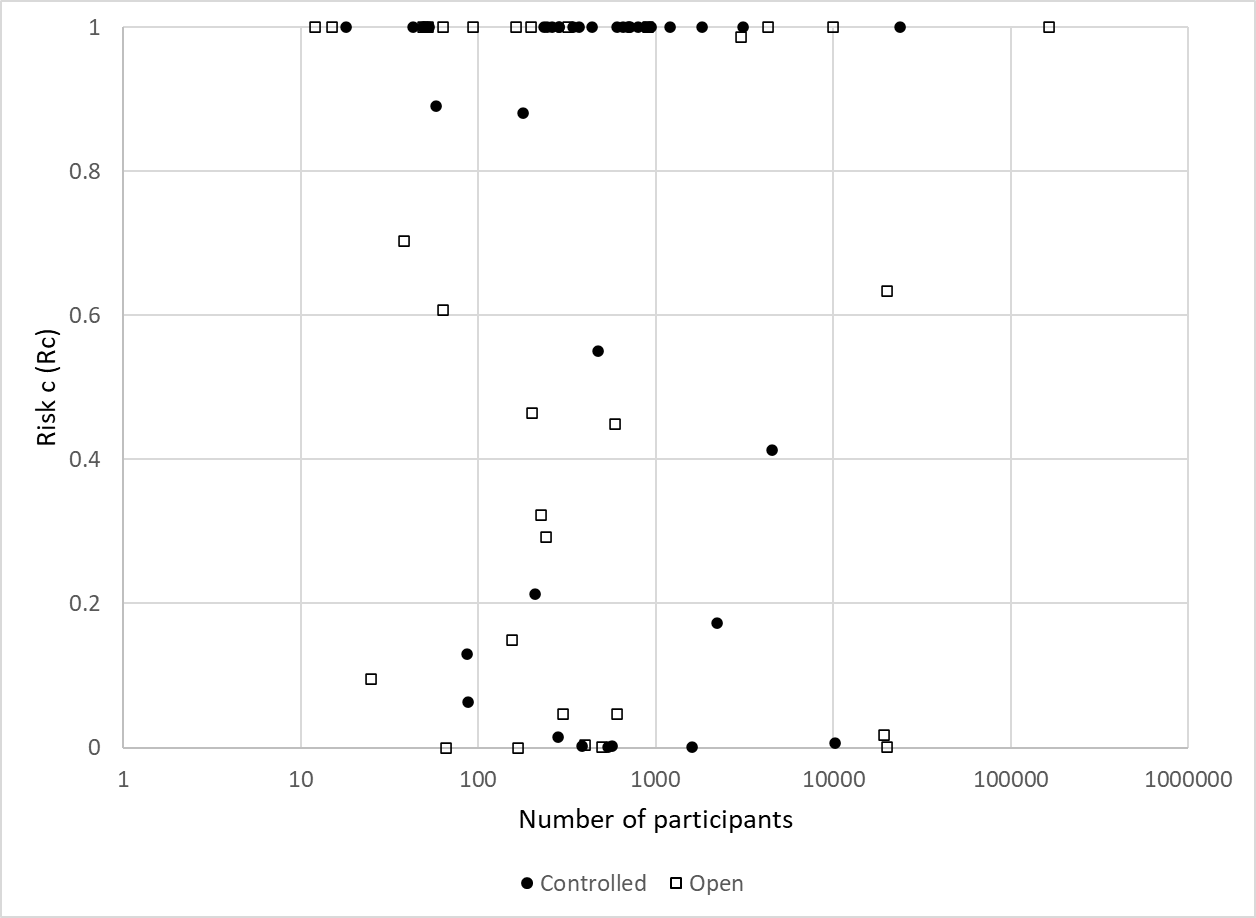 |

| Figure S6.2.6 | Box-and-whisker plots of Rc vs number of indirect identifiers | |
| --- | --- | --- |
| Prosecutor scenario | | Journalist scenario |
| 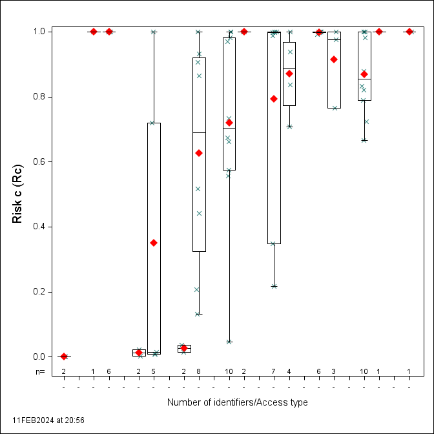 | | 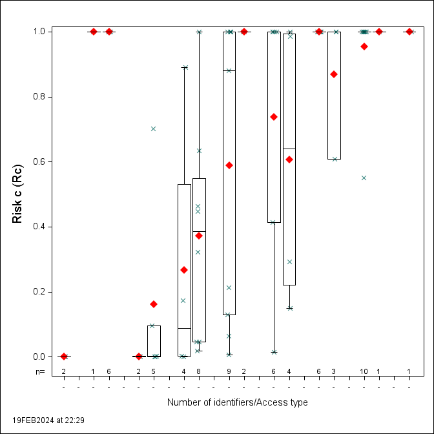 |

| Figure S6.2.7 | Scatterplots of Rb vs Ra | |
| --- | --- | --- |
| Prosecutor scenario | | Journalist scenario |
| 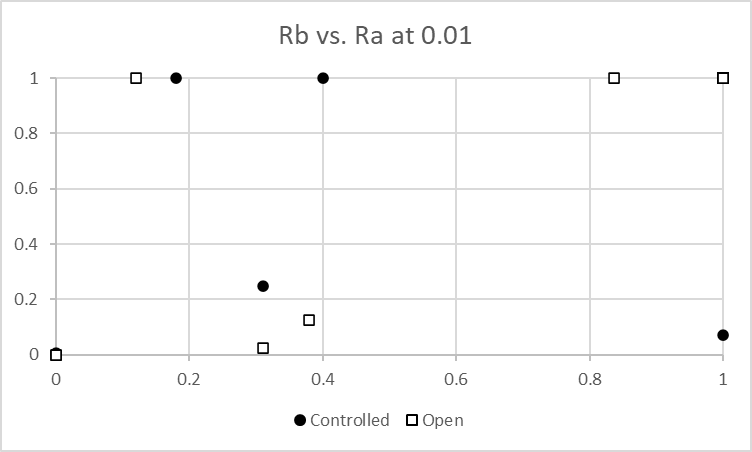 | | 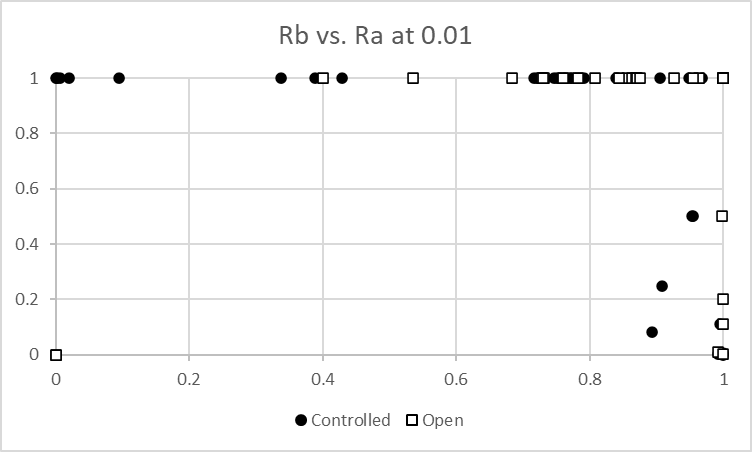 |
| 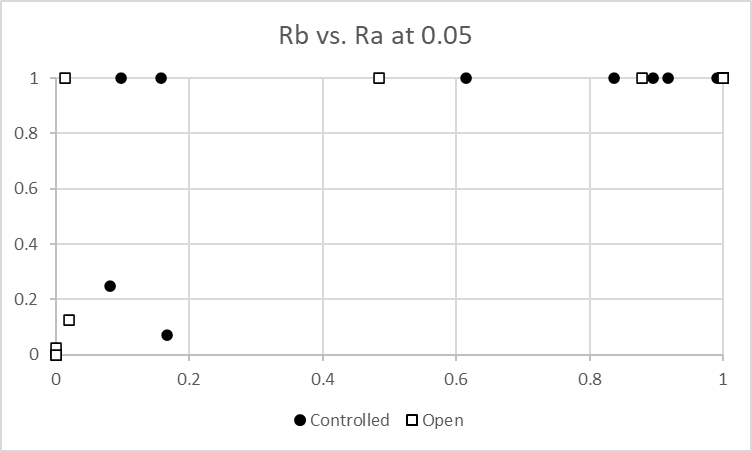 | | 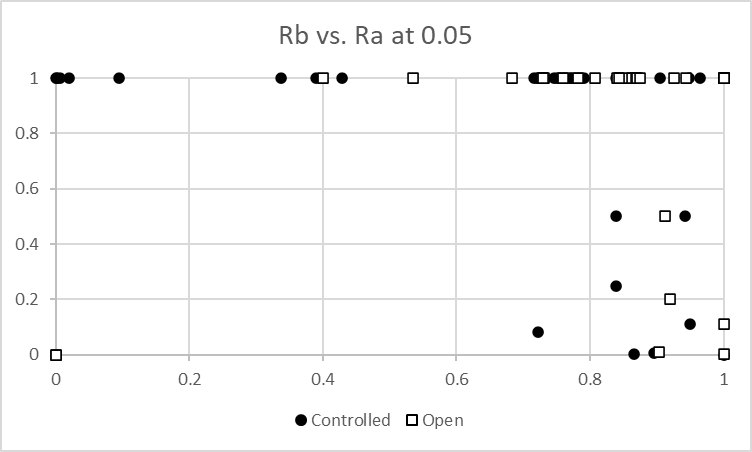 |
| 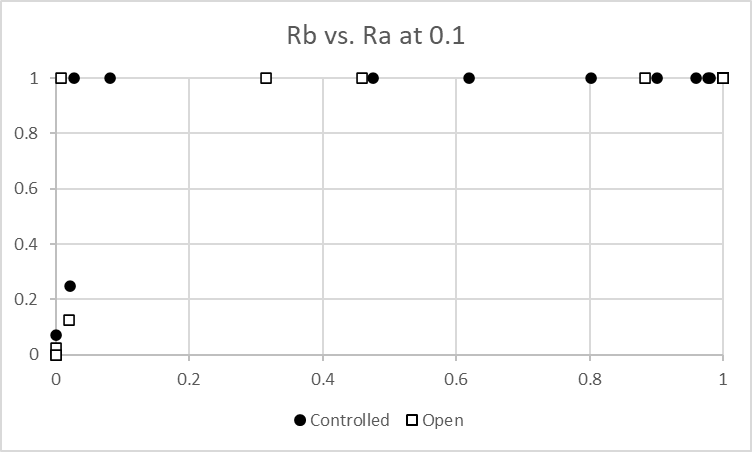 | | 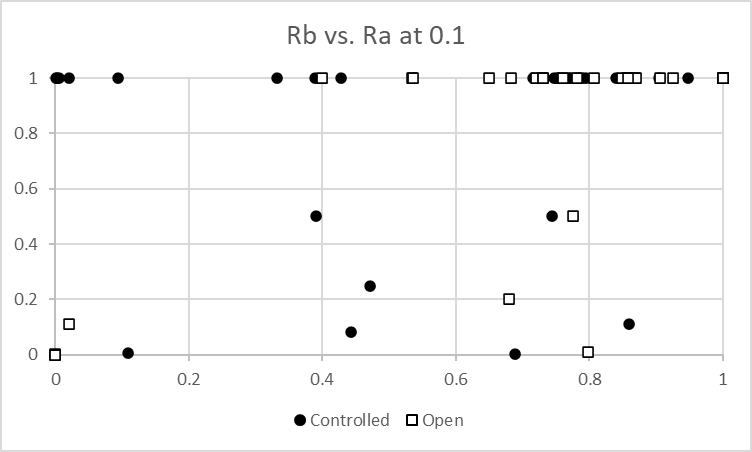 |
| 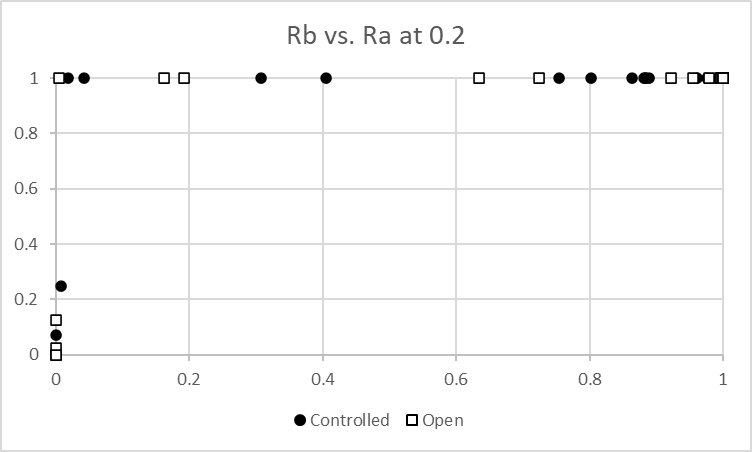 | | 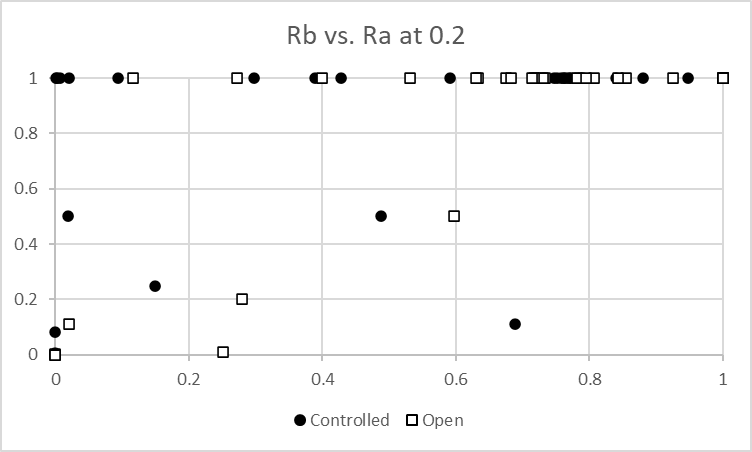 |
| 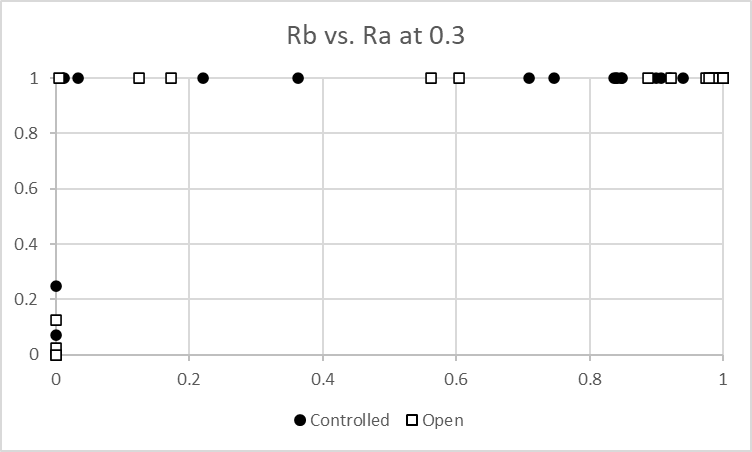 | | 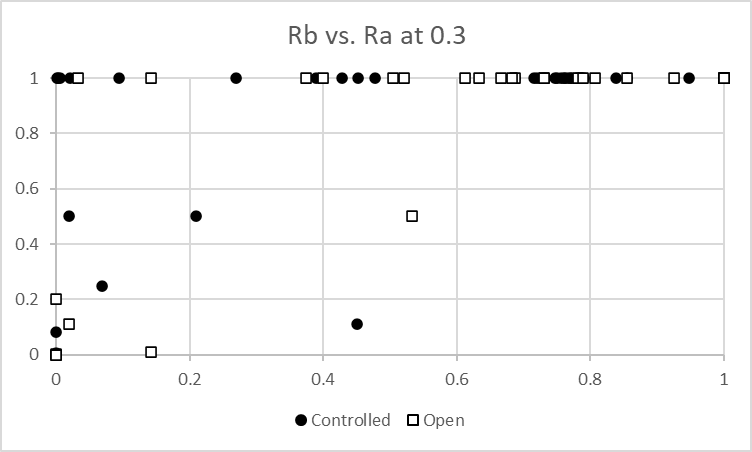 |
| 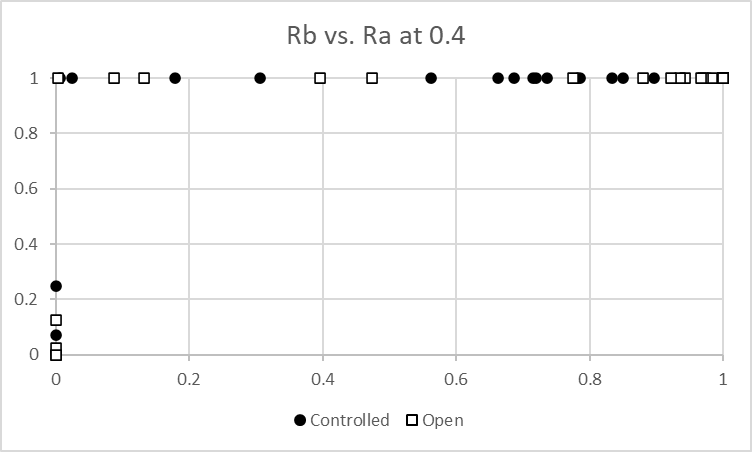 | | 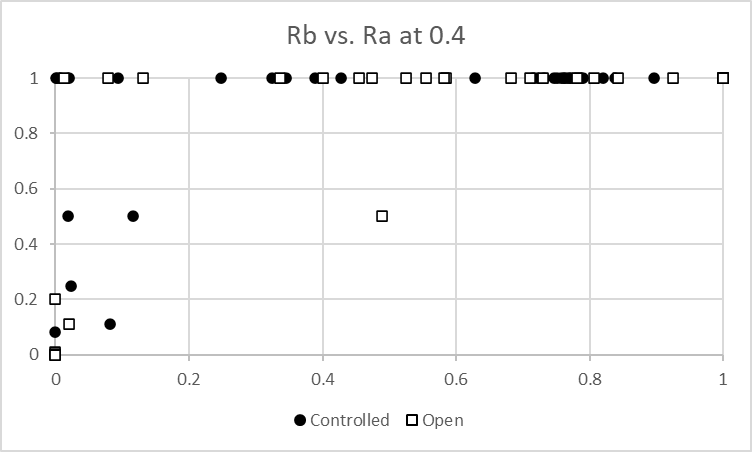 |
| 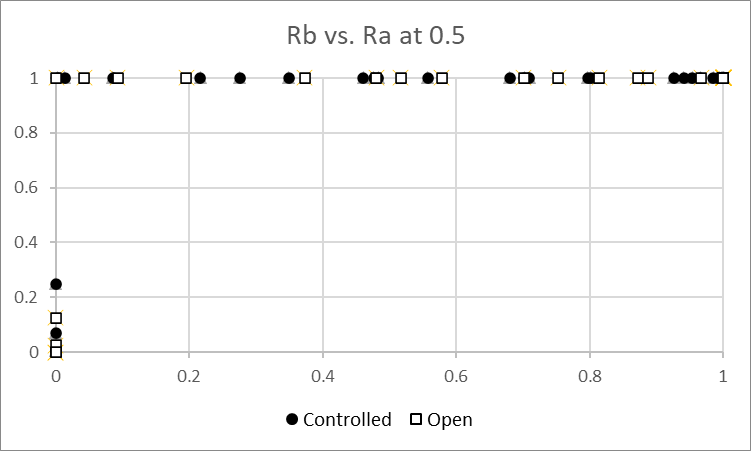 | | 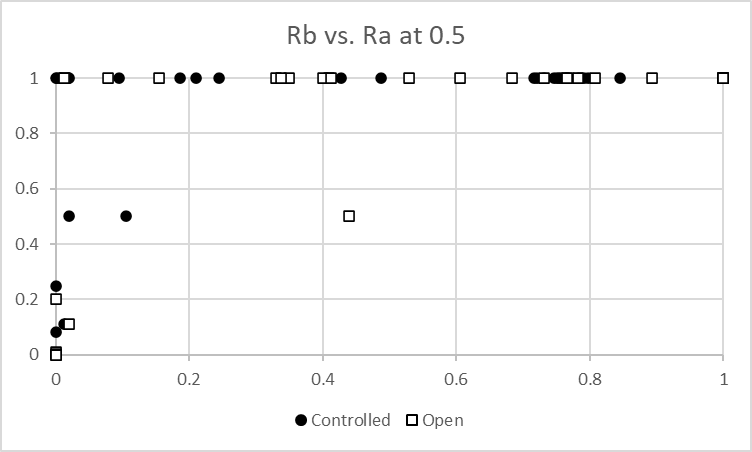 |

| Figure S6.2.8 | Scatterplots of Rc vs Ra | |
| --- | --- | --- |
| Prosecutor scenario | | Journalist scenario |
| 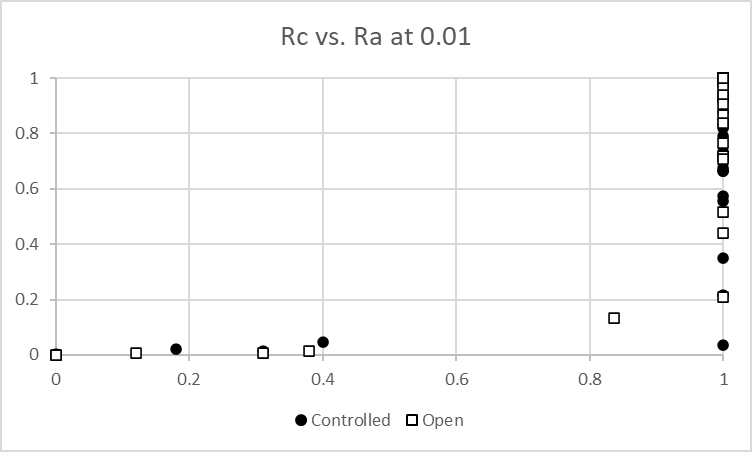 | | 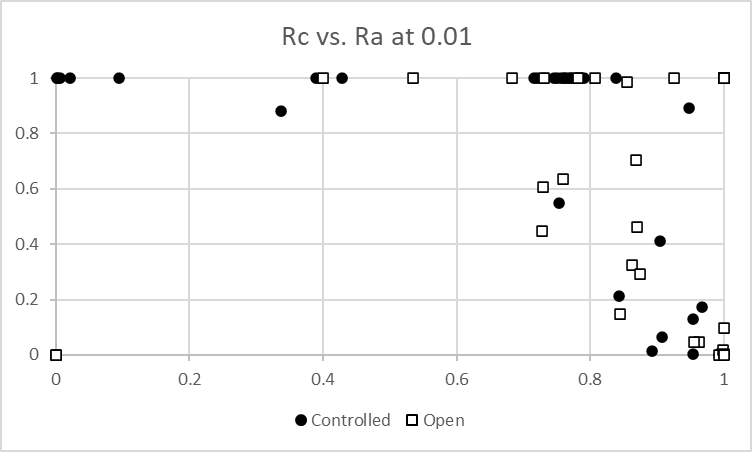 |
| 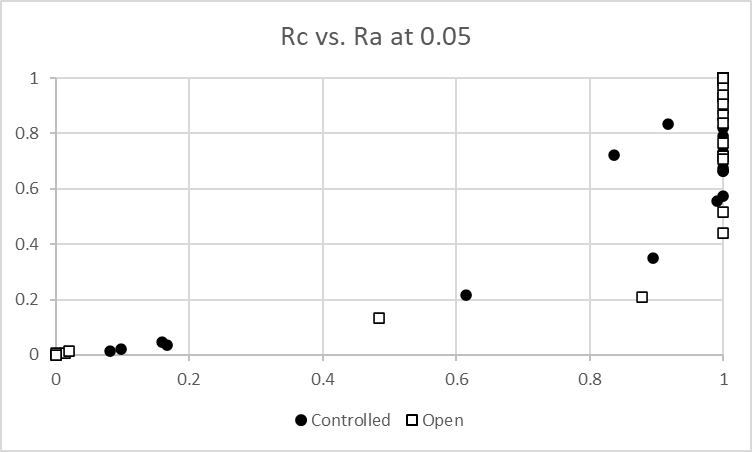 | | 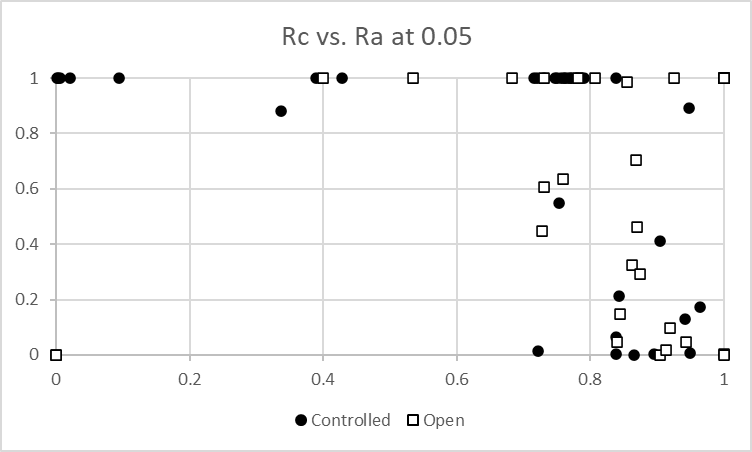 |
| 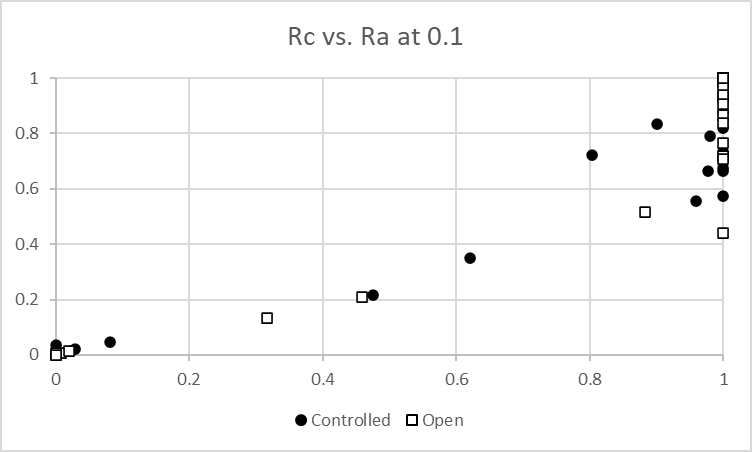 | | 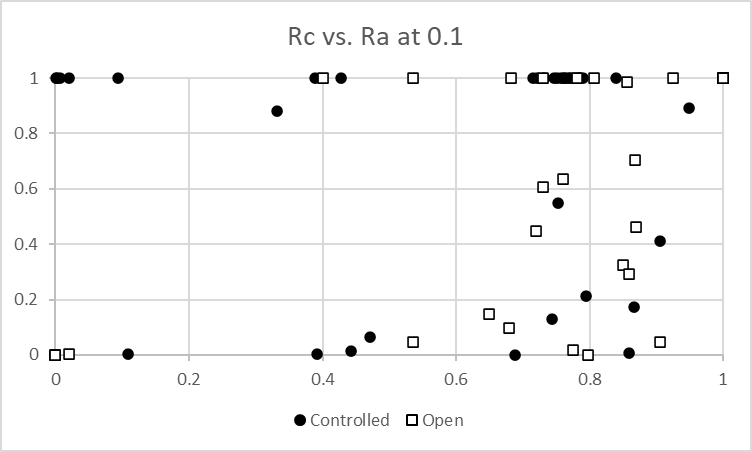 |
| 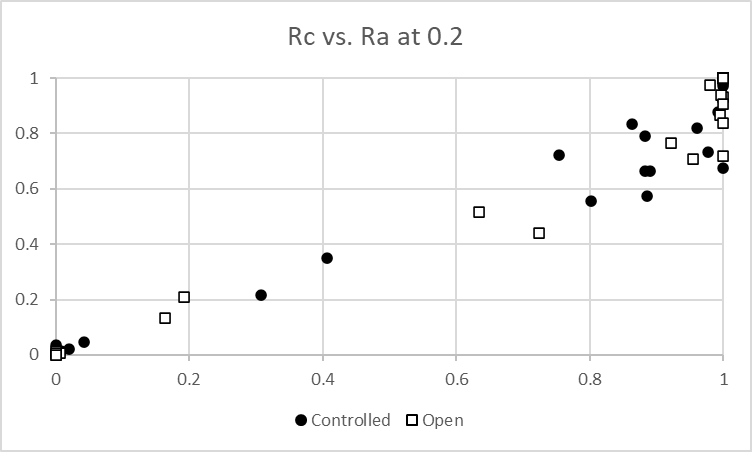 | | 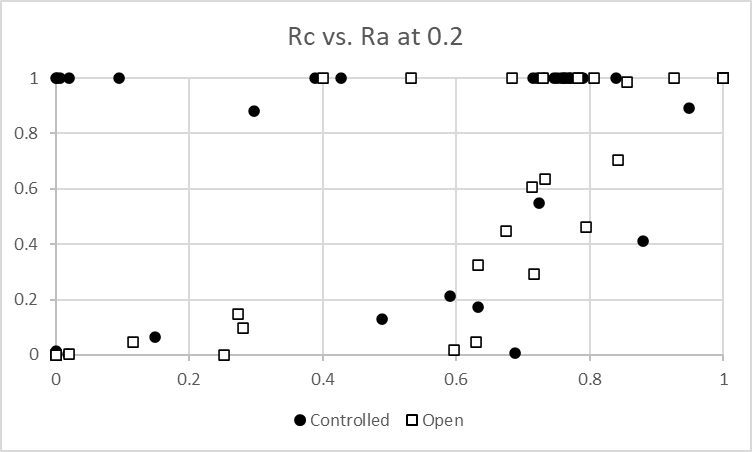 |
| 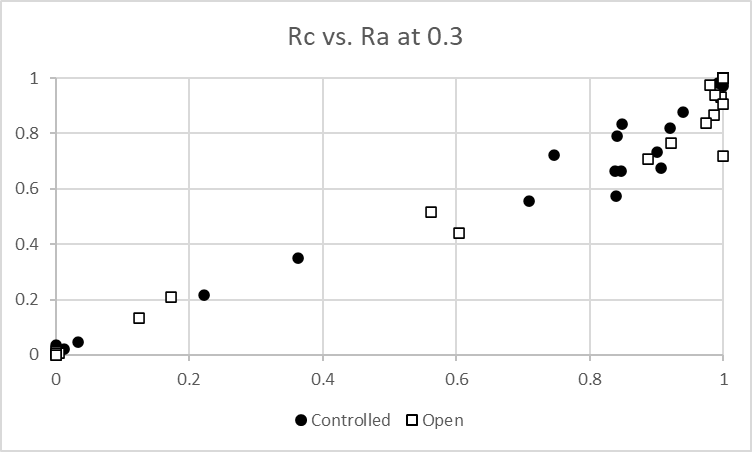 | | 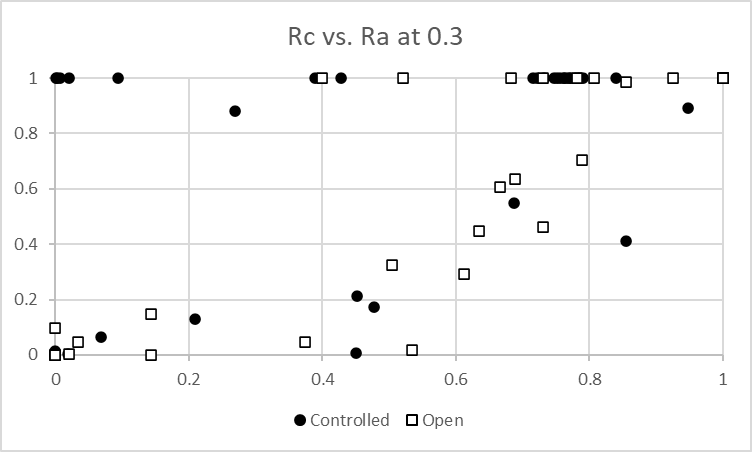 |
| 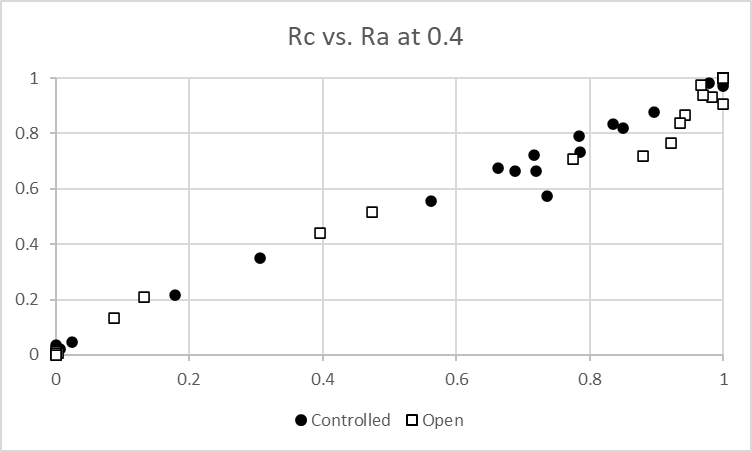 | | 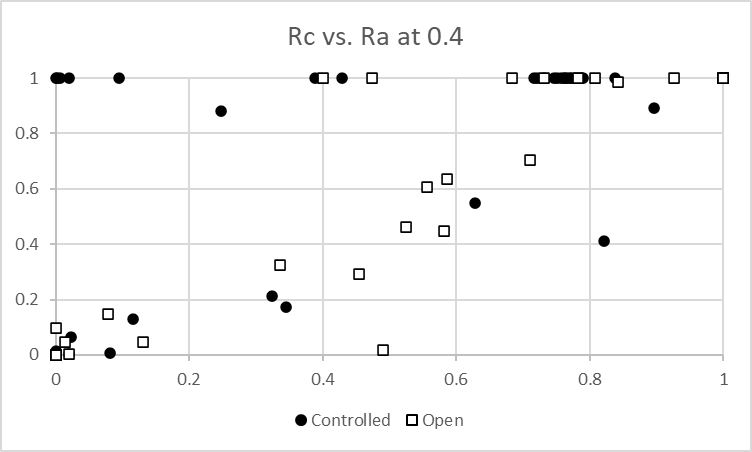 |
| 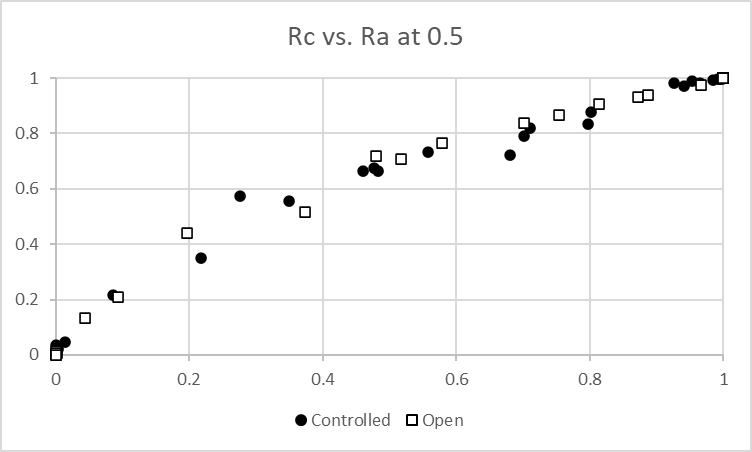 | | 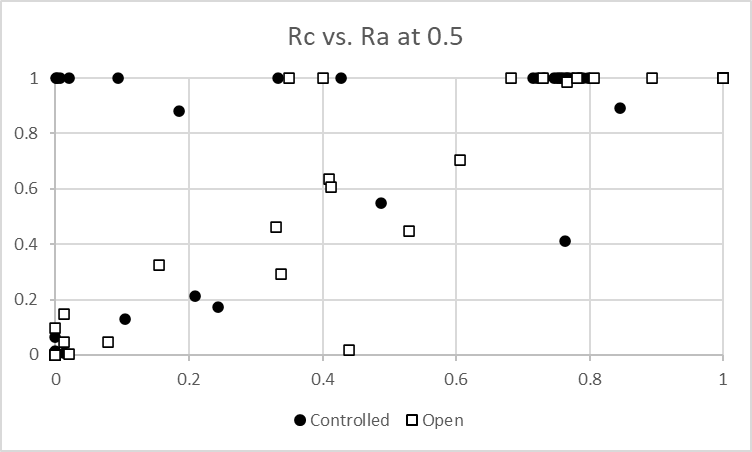 |

| Figure S6.2.9 | Scatterplot of Rb vs Rc | |
| --- | --- | --- |
| Prosecutor scenario | | Journalist scenario |
| 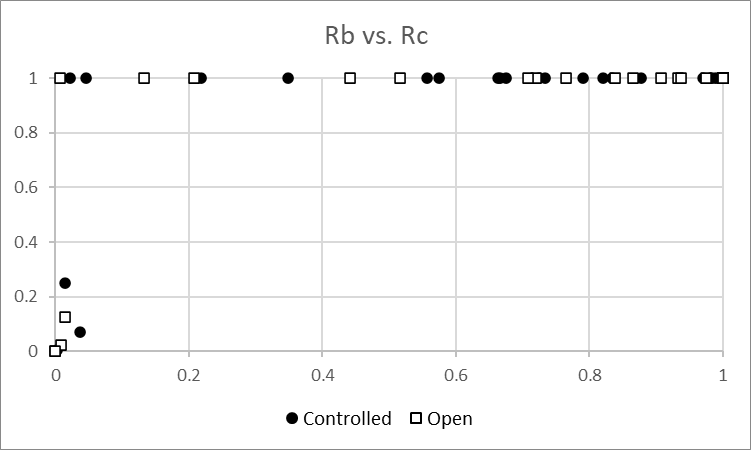 | | 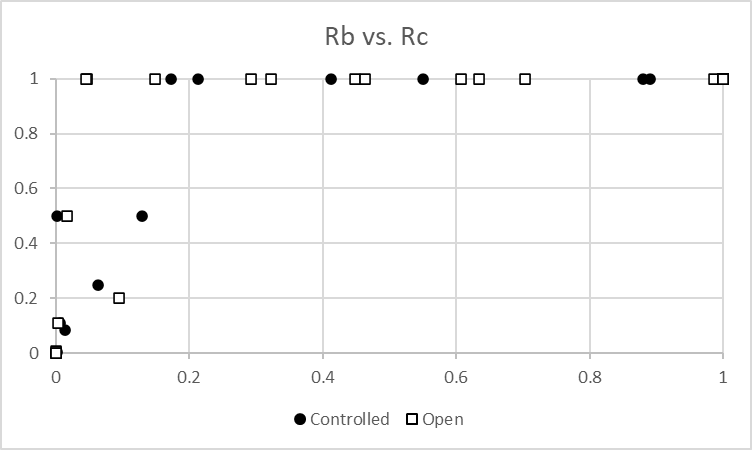 |

| Table S6.3 – **Toppic^1^ trial re-identification risk scores under prosecutor scenario.** | | | | | | | | | | | | | | | |
| --- | --- | --- | --- | --- | --- | --- | --- | --- | --- | --- | --- | --- | --- | --- | --- |
|  | | Indirect Identifiers | | | | Unique levels | Re-identification risk scores | | | | | | | | |
| Categorisation | | Age  (scale / participants) | Sex  (scale / participants) | Height  (scale / participants) | Weight  (scale / participants) |  | Ra 0.01 | Ra 0.05 | Ra 0.1 | Ra 0.2 | Ra 0.3 | Ra 0.4 | Ra 0.5 | Rb | Rc |
| Original  Anonymised dataset | | 235 levels | 2 levels   \| F \| 146 \| \| --- \| --- \| \| M \| 94 \| | 33 levels | 159 levels | 240 | 1 | 1 | 1 | 1 | 1 | 1 | 1 | 1 | 1 |
| Fine - continuous indirect identifiers (age, height and weight) categorised in bands of 10 units | | 8 levels   \| <18 \| 4 \| \| --- \| --- \| \| 18-24 \| 36 \| \| 25-34 \| 64 \| \| 35-44 \| 45 \| \| 45-54 \| 53 \| \| 55-64 \| 28 \| \| 65-74 \| 4 \| \| Missing \| 6 \| | 2 levels   \| F \| 146 \| \| --- \| --- \| \| M \| 94 \| | 6 levels   \| <150 \| 3 \| \| --- \| --- \| \| 150-159 \| 40 \| \| 160-169 \| 89 \| \| 170-179 \| 76 \| \| 180-189 \| 29 \| \| 190-199 \| 3 \| | 9 levels   \| <50 \| 7 \| \| --- \| --- \| \| 50-59 \| 51 \| \| 60-69 \| 62 \| \| 70-79 \| 52 \| \| 80-89 \| 34 \| \| 90-99 \| 14 \| \| 100-109 \| 6 \| \| 110=> \| 3 \| \| Missing \| 11 \| | 123 | 1 | 1 | 1 | 0.82 | 0.75 | 0.49 | 0.27 | 1 | 0.51 |
| Medium - continuous indirect identifiers (age, height and weight) categorised in bands of 20 units | | 5 levels   \| <25 \| 40 \| \| --- \| --- \| \| 25-44 \| 109 \| \| 45-64 \| 81 \| \| 65-84 \| 4 \| \| Missing \| 6 \| | 2 levels   \| F \| 146 \| \| --- \| --- \| \| M \| 94 \| | 4 levels   \| <150 \| 3 \| \| --- \| --- \| \| 150-169 \| 129 \| \| 170-189 \| 105 \| \| 190=> \| 3 \| | 5 levels   \| <60 \| 58 \| \| --- \| --- \| \| 60-79 \| 114 \| \| 80-99 \| 48 \| \| 100=> \| 9 \| \| Missing \| 11 \| | 52 | 1 | 0.73 | 0.50 | 0.29 | 0.24 | 0.15 | 0.07 | 1 | 0.22 |
| Coarse – as medium but strata with counts less than 5 collapsed with their most adjacent stratum | | 4 levels   \| <25 \| 40 \| \| --- \| --- \| \| 25-44 \| 109 \| \| 45=> \| 85 \| \| Missing \| 6 \| | 2 levels   \| F \| 146 \| \| --- \| --- \| \| M \| 94 \| | 2 levels   \| <170 \| 132 \| \| --- \| --- \| \| 170=> \| 108 \| | 5 levels   \| <60 \| 58 \| \| --- \| --- \| \| 60-79 \| 114 \| \| 80-99 \| 48 \| \| 100=> \| 9 \| \| Missing \| 11 \| | 44 | 1 | 0.70 | 0.46 | 0.26 | 0.18 | 0.13 | 0.05 | 1 | 0.18 |
| Coarse with age removed | | Removed | 2 levels   \| F \| 146 \| \| --- \| --- \| \| M \| 94 \| | 2 levels   \| <170 \| 132 \| \| --- \| --- \| \| 170=> \| 108 \| | 5 levels   \| <60 \| 58 \| \| --- \| --- \| \| 60-79 \| 114 \| \| 80-99 \| 48 \| \| 100=> \| 9 \| \| Missing \| 11 \| | 17 | 1 | 0.31 | 0.20 | 0.04 | 0.04 | 0.02 | 0.01 | 1 | 0.07 |
| Coarse with weight removed | | 4 levels   \| <25 \| 40 \| \| --- \| --- \| \| 25-44 \| 109 \| \| 45=> \| 85 \| \| Missing \| 6 \| | 2 levels   \| F \| 146 \| \| --- \| --- \| \| M \| 94 \| | 2 levels   \| <170 \| 132 \| \| --- \| --- \| \| 170=> \| 108 \| | Removed | 15 | 1 | 0.30 | 0.13 | 0.08 | 0.03 | 0.01 | 0.004 | 1 | 0.06 |
| Notes | 1 Mowat C, Arnott I, Cahill A, et al. Mercaptopurine versus placebo to prevent recurrence of Crohn's disease after surgical resection (TOPPIC): a multicentre, double-blind, randomised controlled trial. The lancet Gastroenterology & hepatology 2016; 1: 273-282. (This trial has 240 participants) | | | | | | | | | | | | | | |
| Where | Risk a (Ra): the proportions of participants in strata above a predetermined risk threshold, Risk b (Rb): the stratum with the smallest membership in the anonymised dataset, and Risk c (Rc): the average risk score across the whole strata of the anonymised dataset, using all indirect identifiers, calculated with the formulas in chapter 16 from “Guide to the de-identification of personal health information” by Khaled El Emam (2013) | | | | | | | | | | | | | | |

| Table S6.4 – **Re-identification risk scores under prosecutor scenario for two datasets with three indirect identifiers.** | | | | | | | | | | | | | | | | |
| --- | --- | --- | --- | --- | --- | --- | --- | --- | --- | --- | --- | --- | --- | --- | --- | --- |
|  | | Indirect identifiers | | | | Number of participants | Unique levels | Re-identification risk scores | | | | | | | | |
| ID | | Age | Sex | Country | Ethnicity |  |  | Ra  0.01 | Ra  0.05 | Ra  0.1 | Ra 0.2 | Ra 0.3 | Ra  0.4 | Ra  0.5 | Rb | Rc |
| Dataset 1^1^  (RESTART) | | 2 levels | 2 levels | -- | 2 levels | 537 | 8 | 0.31 | 0.08 | 0.02 | 0.007 | 0 | 0 | 0 | 0.25 | 0.015 |
| Dataset 2^2^  (IST) | | 82 levels | 2 levels | 32 levels | -- | 19435 | 2570 | 0.83 | 0.48 | 0.31 | 0.16 | 0.12 | 0.09 | 0.04 | 1 | 0.13 |
| Notes: | 1 Salman, R.A.-S., et al., Effects of antiplatelet therapy after stroke due to intracerebral haemorrhage (RESTART): a randomised, open-label trial. The Lancet, 2019. 393(10191): p. 2613-2623  2 Group, I.S.T.C., The International Stroke Trial (IST): a randomised trial of aspirin, subcutaneous heparin, both, or neither among 19 435 patients with acute ischaemic stroke. The Lancet, 1997. 349(9065): p. 1569-1581 | | | | | | | | | | | | | | | |
| Where: | Risk a (Ra): the proportions of participants in strata above a predetermined risk threshold, Risk b (Rb): the stratum with the smallest membership in the anonymised dataset, and Risk c (Rc): the average risk score across the whole strata of the anonymised dataset, using all indirect identifiers, calculated with the formulas in chapter 16 from “Guide to the de-identification of personal health information” by Khaled El Emam (2013) | | | | | | | | | | | | | | | |
