# Supplementary material for: Evaluating re-identification risks scores in publicly available clinical trial datasets: Insights and implications
Source: Clin Trials. 2025 Aug 22;22(6):649–66. doi: 10.1177/17407745251356423 (PMC12647387; doi:10.1177/17407745251356423)
Supplement: sj-docx-4-ctj-10.1177_17407745251356423 – Supplemental material for Evaluating re-identification risks scores in publicly available clinical trial datasets: Insights and implications [file sj-docx-4-ctj-10.1177_17407745251356423.docx]

Appendix 5 Data sources requests details and datasets included

| Table S5.1 – Data sources requests details and datasets included |
| --- |

| id | Repository | Country | Pre selected | Requested | Access Provided | | Included Studies |
| --- | --- | --- | --- | --- | --- | --- | --- |
|  |  |  |  |  | Controlled | Open |  |
| 1 | https://datacompass.lshtm.ac.uk ^1^ | UK | 36 | 7 | 2 | 1 | NCT02104232^2^  NCT02111915^3^  ISRCTN36436933^4^ |
| 2 | https://ctu-app.lshtm.ac.uk/freebird ^5^ | UK | 6 | 6 | -- | 5 | ISRCTN7445979^6^  NCT00375258^7^  NCT00872469^8^  NCT00872469^9^  NCT03777488^10^ |
| 3 | https://datashare.is.ed.ac.uk ^11^ | UK | 31 | 5 | 2 | 3 | ISRCTN45178534^12^  ISRCTN25765518^13^  IST (Registration not required)^14^  ISRCTN71907627^15^  ISRCTN89489788^16^ |
| 4 | https://www.clinicalstudydatarequest.com ^17^ | USA | 3058 | 6 | 5 | -- | Registration not required^18^  NCT01822899^19^  NCT01842607^20^  NCT01405053^21^  NCT00948766^22^ |
| 5 | http://datadryad.org ^23^ | UK | 223 | 5 | -- | 4 | ACTRN12616000888460^24^  HKCTR-1848^25^  No registered^26^  NCT04523831^27^ |
| 6 | http://yoda.yale.edu ^28^ | USA | 410 | 5 | 4 | -- | NCT01715285^29^  NCT00903331^30^  NCT01004432^31^  NCT00211133^32^ |
| 7 | https://www.projectdatasphere.org ^33^ | USA | 192 | 9 | 9 | -- | NCT00058474^34^  NCT00033293^35^  NCT00310180^36^  NCT00693992^37^  NCT00312208^38^  NCT00143455^39^  NCT00113763^40^  NCT00617669^41^  NCT00676650^42^ |
| 8 | https://biolincc.nhlbi.nih.gov/studies ^43^ | USA | 195 | 6 | 6 | -- | NCT00650091^44^  NCT00000589^45^  NCT00075829^46^  NCT01982968^47^  NCT01134783^48^  NCT00004562^49^ |
| 9 | https://nda.nih.gov/get/access-data.html ^50^ | USA | 181 | 6 | 5 | -- | NCT00012558^51^  Registration not found^52^  NCT01927276^53^  NCT01944046^54^  NCT00005013^55^ |
| 10 | https://vivli.org/ ^56^ | USA | 3394 | 8 | 5 | -- | NCT01198756^57^  NCT01573767^58^  NCT01313676^59^  NCT00400855^60^  NCT01498822^61^ |
| 11 | https://beta.ukdataservice.ac.uk/datacatalogue/studies ^62^ | UK | 21 | 5 | 1 | 4 | ISRCTN11288961^63^  ISRCTN90749868^64^  NCT01801410^65^  Registration not required^66^  ISRCTN24081411^67^ |
| 12 | https://med.data.edu.au/find-data/ ^68^ | Australia | -- | 0 | -- | -- | -- |
| 13 | https://dcri.org/our-approach/data-sharing/soar-data ^69^ | USA | 2 | 2 | -- | -- | -- |

| Table S5.1 – Data sources requests details and datasets selected |
| --- |

| id | Repository | Country | Pre selected | Requested | Access Provided | | Studies |
| --- | --- | --- | --- | --- | --- | --- | --- |
|  |  |  |  |  | Controlled | Open |  |
| 14 | https://journals.plos.org/plosone/search ^70^ | UK | 5944 | 6 | -- | 6 | NCT02700490^71^  TCTR20201005002^72^  NCT02185196^73^  ACTRN12616000538448^74^  ISRCTN 71217488^75^  NCT02747524^76^ |
| 15 | https://www.bmj.com/search/advanced ^77^ | UK | 934 | 5 | -- | 3 | NCT02068885^78^  NCT01953549^79^  ISRCTN11980540^80^ |
| 16 | https://dataverse.harvard.edu/ ^81^ | USA | 271 | 5 | -- | 5 | CTRI/2016/09/007240^82^  PACTR201901905832601^83^  NCT02148952^84^  SLCTR/2019/015^85^  ANZCTR12616001367437^86^ |
| 17 | https://arlg.org/studies-in-progress/ ^87^ | USA | -- | 0 | -- | -- | -- |
| 18 | https://repository.niddk.nih.gov/studies/ ^88^ | USA | -- | 0 | -- | -- | -- |

**Reference List**

1. London School of Hygiene & Tropical Medicine. LSHTM Data Compass, <https://datacompass.lshtm.ac.uk>.

2. Fuhr DC, Weobong B, Lazarus A, et al. Delivering the Thinking Healthy Programme for perinatal depression through peers: an individually randomised controlled trial in India. *The Lancet Psychiatry* 2019; 6: 115-127.

3. Sikander S, Ahmad I, Atif N, et al. Delivering the Thinking Healthy Programme for perinatal depression through volunteer peers: a cluster randomised controlled trial in Pakistan. *The Lancet Psychiatry* 2019; 6: 128-139.

4. Dhalla K, Cousens S, Bowman R, et al. Is beta radiation better than 5 flurouracil as an adjunct for trabeculectomy surgery when combined with cataract surgery? A randomised controlled trial. *PLoS One* 2016; 11: e0161674.

5. Clinical Trials Unit London School of Hygiene & Tropical Medicine. The FreeBIRD Bank of Injury and Emergency Research Data, <https://freebird.lshtm.ac.uk/>.

6. Collaborators CT. Effect of intravenous corticosteroids on death within 14 days in 10 008 adults with clinically significant head injury (MRC CRASH trial): randomised placebo-controlled trial. *The Lancet* 2004; 364: 1321-1328.

7. collaborators C-t. Effects of tranexamic acid on death, vascular occlusive events, and blood transfusion in trauma patients with significant haemorrhage (CRASH-2): A randomised, placebo-controlled trial. *the Lancet* 2010; 376: 376: 323-332. DOI: <https://doi.org/10.1016/S0140-6736(10)60835-5>.

8. Shakur H, Roberts I, Fawole B, et al. Effect of early tranexamic acid administration on mortality, hysterectomy, and other morbidities in women with post-partum haemorrhage (WOMAN): an international, randomised, double-blind, placebo-controlled trial. *The Lancet* 2017; 389: 2105-2116.

9. Shakur-Still H, Roberts I, Fawole B, et al. Effect of tranexamic acid on coagulation and fibrinolysis in women with postpartum haemorrhage (WOMAN-ETAC): a single-centre, randomised, double-blind, placebo-controlled trial. *Wellcome open research* 2018; 3.

10. Grassin-Delyle S, Semeraro M, Lamy E, et al. Pharmacokinetics of tranexamic acid after intravenous, intramuscular, and oral routes: a prospective, randomised, crossover trial in healthy volunteers. *British Journal of Anaesthesia* 2022; 128: 465-472.

11. The University of Edinburgh. Edinburgh DataShare, <https://datashare.ed.ac.uk/>.

12. Lewis SC, Bhattacharya S, Wu O, et al. Gabapentin for the management of chronic pelvic pain in women (GaPP1): a pilot randomised controlled trial. *PloS one* 2016; 11: e0153037.

13. Group I-C. The benefits and harms of intravenous thrombolysis with recombinant tissue plasminogen activator within 6 h of acute ischaemic stroke (the third international stroke trial [IST-3]): a randomised controlled trial. *The Lancet* 2012; 379: 2352-2363.

14. Group ISTC. The International Stroke Trial (IST): a randomised trial of aspirin, subcutaneous heparin, both, or neither among 19 435 patients with acute ischaemic stroke. *The Lancet* 1997; 349: 1569-1581.

15. Salman RA-S, Dennis M, Sandercock P, et al. Effects of antiplatelet therapy after stroke due to intracerebral haemorrhage (RESTART): a randomised, open-label trial. *The Lancet* 2019; 393: 2613-2623.

16. Mowat C, Arnott I, Cahill A, et al. Mercaptopurine versus placebo to prevent recurrence of Crohn's disease after surgical resection (TOPPIC): a multicentre, double-blind, randomised controlled trial. *The lancet Gastroenterology & hepatology* 2016; 1: 273-282.

17. Clinical Study Data Request (CSDR). Clinical Study Data Request, <https://clinicalstudydatarequest.com/> (2020, accessed 26 Oct 2020 2020).

18. Hayden FG, Osterhaus AD, Treanor JJ, et al. Efficacy and safety of the neuraminidase inhibitor zanamivir in the treatment of influenzavirus infections. *New England Journal of Medicine* 1997; 337: 874-880.

19. Singh D, Worsley S, Zhu C-Q, et al. Umeclidinium/vilanterol versus fluticasone propionate/salmeterol in COPD: a randomised trial. *BMC Pulmonary Medicine* 2015; 15: 1-12.

20. Lugogo N, Domingo C, Chanez P, et al. Long-term efficacy and safety of mepolizumab in patients with severe eosinophilic asthma: a multi-center, open-label, phase IIIb study. *Clinical therapeutics* 2016; 38: 2058-2070. e2051.

21. Arzimanoglou A, Ferreira J, Satlin A, et al. Evaluation of long-term safety, tolerability, and behavioral outcomes with adjunctive rufinamide in pediatric patients (≥ 1 to< 4 years old) with Lennox-Gastaut syndrome: final results from randomized study 303. *European Journal of Paediatric Neurology* 2019; 23: 126-135.

22. T Grossberg G, R Farlow M, Meng X, et al. Evaluating high-dose rivastigmine patch in severe Alzheimer’s disease: analyses with concomitant memantine usage as a factor. *Current Alzheimer Research* 2015; 12: 53-60.

23. Dryad. Data Dryad, <https://datadryad.org/>.

24. Darlow B, Stanley J, Dean S, et al. The Fear Reduction Exercised Early (FREE) approach to management of low back pain in general practice: a pragmatic cluster-randomised controlled trial. *PLoS medicine* 2019; 16: e1002897.

25. Zee K-Y, Chan PS, Ho JCS, et al. Adjunctive use of modified Yunu-Jian in the non-surgical treatment of male smokers with chronic periodontitis: a randomized double-blind, placebo-controlled clinical trial. *Chinese Medicine* 2016; 11: 1-13.

26. Christopher PP, Appelbaum PS, Truong D, et al. Reducing therapeutic misconception: A randomized intervention trial in hypothetical clinical trials. *PLoS One* 2017; 12: e0184224.

27. Mahmud R, Rahman MM, Alam I, et al. Ivermectin in combination with doxycycline for treating COVID-19 symptoms: a randomized trial. *Journal of International Medical Research* 2021; 49: 03000605211013550.

28. The Yale University. Yale University Open Data Access (YODA) Project, <http://yoda.yale.edu/> (2020, accessed 26 Oct 2020 2020).

29. Fizazi K, Tran N, Fein L, et al. Abiraterone plus prednisone in metastatic, castration-sensitive prostate cancer. *New England Journal of Medicine* 2017; 377: 352-360.

30. Raghu G, Million-Rousseau R, Morganti A, et al. Macitentan for the treatment of idiopathic pulmonary fibrosis: the randomised controlled MUSIC trial. *European Respiratory Journal* 2013; 42: 1622-1632.

31. Huffstutter JE, Kafka S, Brent LH, et al. Clinical response to golimumab in rheumatoid arthritis patients who were receiving etanercept or adalimumab: results of a multicenter active treatment study. *Current Medical Research and Opinion* 2017; 33: 657-666.

32. Leyland-Jones B, Semiglazov V, Pawlicki M, et al. Maintaining normal hemoglobin levels with epoetin alfa in mainly nonanemic patients with metastatic breast cancer receiving first-line chemotherapy: a survival study. *Journal of Clinical Oncology* 2005; 23: 5960-5972.

33. CEO Roundtable on Cancer Inc. Project Data Sphere, <https://www.projectdatasphere.org/>.

34. O'Connell MJ, Colangelo LH, Beart RW, et al. Capecitabine and oxaliplatin in the preoperative multimodality treatment of rectal cancer: surgical end points from National Surgical Adjuvant Breast and Bowel Project trial R-04. *Journal of clinical oncology* 2014; 32: 1927.

35. de Alarcon PA, Matthay KK, London WB, et al. Intravenous immunoglobulin with prednisone and risk-adapted chemotherapy for children with opsoclonus myoclonus ataxia syndrome associated with neuroblastoma (ANBL00P3): a randomised, open-label, phase 3 trial. *The Lancet Child & Adolescent Health* 2018; 2: 25-34.

36. Sparano JA, Gray RJ, Makower DF, et al. Adjuvant chemotherapy guided by a 21-gene expression assay in breast cancer. *New England Journal of Medicine* 2018; 379: 111-121.

37. Baggstrom MQ, Socinski MA, Wang XF, et al. Maintenance sunitinib following initial platinum-based combination chemotherapy in advanced-stage IIIB/IV non–small cell lung cancer: a randomized, double-blind, placebo-controlled phase III study—CALGB 30607 (Alliance). *Journal of Thoracic Oncology* 2017; 12: 843-849.

38. Eiermann W, Pienkowski T, Crown J, et al. Phase III study of doxorubicin/cyclophosphamide with concomitant versus sequential docetaxel as adjuvant treatment in patients with human epidermal growth factor receptor 2-normal, node-positive breast cancer: BCIRG-005 trial. *J Clin Oncol* 2011; 29: 3877-3884.

39. Pfizer. *Online report for Open Label, Randomised Multicentre Phase III Study Of Irinotecan Hydrochloride (Campto (Registered)) And Cisplatin Versus Etoposide And Cisplatin In Chemotherapy Naive Patients With Extensive Disease - Small Cell Lung Cancer*. 2010.

40. Poulin-Costello M, Azoulay L, Van Cutsem E, et al. An analysis of the treatment effect of panitumumab on overall survival from a phase 3, randomized, controlled, multicenter trial (20020408) in patients with chemotherapy refractory metastatic colorectal cancer. *Targeted oncology* 2013; 8: 127-136.

41. Fizazi K, Higano CS, Nelson JB, et al. Phase III, randomized, placebo-controlled study of docetaxel in combination with zibotentan in patients with metastatic castration-resistant prostate cancer. *Journal of Clinical Oncology* 2013; 31: 1740-1747.

42. Michaelson MD, Oudard S, Ou Y-C, et al. Randomized, placebo-controlled, phase III trial of sunitinib plus prednisone versus prednisone alone in progressive, metastatic, castration-resistant prostate cancer. *J Clin Oncol* 2014; 32: 76-82.

43. The National Heart LaBIN. BioLINCC, <https://biolincc.nhlbi.nih.gov/>.

44. Network IPFCR. Randomized trial of acetylcysteine in idiopathic pulmonary fibrosis. *New England Journal of Medicine* 2014; 370: 2093-2101.

45. Group TtRAtPS. Leukocyte reduction and ultraviolet B irradiation of platelets to prevent alloimmunization and refractoriness to platelet transfusions. *New England Journal of Medicine* 1997; 337: 1861-1870.

46. Krishnan A, Pasquini MC, Logan B, et al. Autologous haemopoietic stem-cell transplantation followed by allogeneic or autologous haemopoietic stem-cell transplantation in patients with multiple myeloma (BMT CTN 0102): a phase 3 biological assignment trial. *The lancet oncology* 2011; 12: 1195-1203.

47. Raghu G, Pellegrini CA, Yow E, et al. Laparoscopic anti-reflux surgery for the treatment of idiopathic pulmonary fibrosis (WRAP-IPF): a multicentre, randomised, controlled phase 2 trial. *The Lancet Respiratory Medicine* 2018; 6: 707-714.

48. Lytle LA, Laska MN, Linde JA, et al. Weight-gain reduction among 2-year college students: the CHOICES RCT. *American Journal of Preventive Medicine* 2017; 52: 183-191.

49. Hochman JS, Lamas GA, Buller CE, et al. Coronary intervention for persistent occlusion after myocardial infarction. *New England Journal of Medicine* 2006; 355: 2395-2407.

50. The National Institute of Mental Health. The NIMH Data Archive (NDA), <https://nda.nih.gov/>.

51. Sachs GS, Nierenberg AA, Calabrese JR, et al. Effectiveness of adjunctive antidepressant treatment for bipolar depression. *New England Journal of Medicine* 2007; 356: 1711-1722.

52. Kerwin ML. Using SMART Treatment Design to Evaluate Applied Behavior Analysis Interventions on Communication in Preschool Children with Autism.

53. Kelly DL, Demyanovich HK, Rodriguez KM, et al. Randomized controlled trial of a gluten-free diet in patients with schizophrenia positive for antigliadin antibodies (AGA IgG): a pilot feasibility study. *Journal of Psychiatry and Neuroscience* 2019; 44: 269-276.

54. Sikich L, Kolevzon A, King BH, et al. Intranasal oxytocin in children and adolescents with autism spectrum disorder. *New England Journal of Medicine* 2021; 385: 1462-1473.

55. Group HDTS and Group HDTS. Effect of Hypericum perforatum (St John's wort) in major depressive disorder: a randomized controlled trial. *Jama* 2002; 287: 1807-1814.

56. Vivli Center for Global Clinical Research Data. Vivli, a global data-sharing and analytics platform. , <https://vivli.org/> (2020, accessed 30 Oct 2020 2020).

57. Langley JM, Carmona Martinez A, Chatterjee A, et al. Immunogenicity and safety of an inactivated quadrivalent influenza vaccine candidate: a phase III randomized controlled trial in children. *The Journal of infectious diseases* 2013; 208: 544-553.

58. Oliver AJ, Covar RA, Goldfrad CH, et al. Randomised trial of once-daily vilanterol in children with asthma on inhaled corticosteroid therapy. *Respiratory Research* 2016; 17: 1-11.

59. Calverley PM, Anderson JA, Brook RD, et al. Fluticasone furoate, vilanterol, and lung function decline in patients with moderate chronic obstructive pulmonary disease and heightened cardiovascular risk. *American Journal of Respiratory and Critical Care Medicine* 2018; 197: 47-55.

60. GlaxoSminthKline group of companies. *A Randomised, Double-blind, Placebo-controlled, Incomplete Block, 4-period Crossover, Study to Investigate the Effects of 5-day Repeat Inhaled Doses of Fluticasone Propionate (BID, 50-2000 mcg) on Airway Responsiveness to Adenosine 5-monophosphate (AMP) Challenge When Delivered After the Last Dose in Mild Asthmatic Subjects. (GSK 04_SIG103337). Clinical Summary Report* 20 October 2006 2006.

61. Kim JH, Lee SK, Loesch C, et al. Comparison of levetiracetam and oxcarbazepine monotherapy among Korean patients with newly diagnosed focal epilepsy: A long‐term, randomized, open‐label trial. *Epilepsia* 2017; 58: e70-e74.

62. UK Data Service - University of Essex. UK Data Service: data Catalogue, <https://beta.ukdataservice.ac.uk/datacatalogue/studies>.

63. Csipke E, Shafayat A, Sprange K, et al. Promoting independence in dementia (PRIDE): A feasibility randomized controlled trial. *Clinical Interventions in Aging* 2021: 363-378.

64. Yiend J, Lam CL, Schmidt N, et al. Cognitive bias modification for paranoia (CBM-pa): a randomised controlled feasibility study in patients with distressing paranoid beliefs. *Psychological medicine* 2023; 53: 4614-4626.

65. Bracken H, Mundle S, Faragher B, et al. Induction of labour in pre-eclamptic women: a randomised trial comparing the Foley balloon catheter with oral misoprostol. *BMC pregnancy and childbirth* 2014; 14: 1-5.

66. McEwan K, Richardson M, Sheffield D, et al. A smartphone app for improving mental health through connecting with urban nature. *International journal of environmental research and public health* 2019; 16: 3373.

67. Murphy AW, Cupples M, Smith S, et al. Effect of tailored practice and patient care plans on secondary prevention of heart disease in general practice: cluster randomised controlled trial. *Bmj* 2009; 339.

68. Intersect Australia Limited - Queensland Cyber Infrastructure Foundation Ltd. Australian National Medical Research Data Storage Facility, <https://med.data.edu.au/find-data/>.

69. Institute DCR. SOAR DATA™, <https://dcri.org/our-approach/data-sharing/soar-data>.

70. PLOS is a nonprofit 501(c)(3) corporation. PLOS ONE: An inclusive journal community working together to advance science by making all rigorous research accessible without barriers, <https://journals.plos.org/plosone/search>.

71. Anjara SG, Bonetto C, Ganguli P, et al. Can General Practitioners manage mental disorders in primary care? A partially randomised, pragmatic, cluster trial. *PLoS One* 2019; 14: e0224724.

72. Vijitpavan A, Kittikunakorn N and Komonhirun R. Comparison between intrathecal morphine and intravenous patient control analgesia for pain control after video-assisted thoracoscopic surgery: A pilot randomized controlled study. *Plos one* 2022; 17: e0266324.

73. Chowdhury F, Shahid ASMSB, Tabassum M, et al. Vitamin D supplementation among Bangladeshi children under-five years of age hospitalised for severe pneumonia: A randomised placebo controlled trial. *Plos one* 2021; 16: e0246460.

74. Weinberg L, Ianno D, Churilov L, et al. Restrictive intraoperative fluid optimisation algorithm improves outcomes in patients undergoing pancreaticoduodenectomy: a prospective multicentre randomized controlled trial. *PLoS One* 2017; 12: e0183313.

75. Choi W, Kim JC, Kim WS, et al. Clinical effect of antioxidant glasses containing extracts of medicinal plants in patients with dry eye disease: a multi-center, prospective, randomized, double-blind, placebo-controlled trial. *PLoS One* 2015; 10: e0139761.

76. Iannotti L, Dulience SJ-L, Joseph S, et al. Fortified snack reduced anemia in rural school-aged children of Haiti: a cluster-randomized, controlled trial. *PloS one* 2016; 11: e0168121.

77. BMJ Publishing Group Ltd. BMJ is a global healthcare knowledge provider with a vision for a healthier world. We share knowledge and expertise to improve healthcare outcomes., <https://www.bmj.com/search/advanced>.

78. Ebbeling CB, Feldman HA, Klein GL, et al. Effects of a low carbohydrate diet on energy expenditure during weight loss maintenance: randomized trial. *bmj* 2018; 363.

79. Nave AH, Rackoll T, Grittner U, et al. Physical Fitness Training in Patients with Subacute Stroke (PHYS-STROKE): multicentre, randomised controlled, endpoint blinded trial. *Bmj* 2019; 366.

80. Costa ML, Achten J, Ooms A, et al. Surgical fixation with K-wires versus casting in adults with fracture of distal radius: DRAFFT2 multicentre randomised clinical trial. *bmj* 2022; 376.

81. Harvard University. Harvard Dataverse Repository. Deposit and share your data. Get academic credit. Harvard Dataverse is a repository for research data. Deposit data and code here., <https://dataverse.harvard.edu/>.

82. Gehani M, Kapur S, Madhuri SD, et al. Effectiveness of antenatal screening of asymptomatic bacteriuria in reduction of prematurity and low birth weight: Evaluating a point-of-care rapid test in a pragmatic randomized controlled study. *EClinicalMedicine* 2021; 33.

83. Elson L, Randu K, Feldmeier H, et al. Efficacy of a mixture of neem seed oil (Azadirachta indica) and coconut oil (Cocos nucifera) for topical treatment of tungiasis. A randomized controlled, proof-of-principle study. *PLoS Neglected Tropical Diseases* 2019; 13: e0007822.

84. Semrau KE, Hirschhorn LR, Marx Delaney M, et al. Outcomes of a coaching-based WHO safe childbirth checklist program in India. *New England Journal of Medicine* 2017; 377: 2313-2324.

85. Jayawardane M, Piyadigama I and Chandradeva U. Will a preoperative theatre visit reduce anxiety? A randomised controlled trial. *Journal of Obstetrics and Gynaecology* 2022; 42: 1498-1503.

86. Stitely ML, Harlow K and MacKenzie E. Oral riboflavin to assess ureteral patency during cystoscopy: a randomized clinical trial. *Obstetrics & Gynecology* 2019; 133: 301-307.

87. Antibacterial Resistance Leadership Group (ARLG) ARLG studies, <https://arlg.org/summary-of-results/>.

88. National Institute of Diabetes and Digestive and Kidney Diseases (NIDDK). NIDDK Central Repository, <https://repository.niddk.nih.gov/studies/dpp/>.
