# Supplementary material for: Evaluating re-identification risks scores in publicly available clinical trial datasets: Insights and implications
Source: Clin Trials. 2025 Aug 22;22(6):649–66. doi: 10.1177/17407745251356423 (PMC12647387; doi:10.1177/17407745251356423)
Supplement: sj-docx-5-ctj-10.1177_17407745251356423 – Supplemental material for Evaluating re-identification risks scores in publicly available clinical trial datasets: Insights and implications [file sj-docx-5-ctj-10.1177_17407745251356423.docx]

Appendix 4 Worked example for Calculating Re-identification risk scores

- **Background**

El-Emam Risk calculation – The equations in table S4.1.1 and S4.1.2 to calculate re-identification risks scores were taken from El Emam K. Guide to the de-identification of personal health information. Chapter 16. CRC Press; 2013 May 6. (El Emam 2013)

Table S4.1.1 shows the general equations to calculation the re-identification risk scores.

| Table S4.1.1. Re-identification Risk Scores. | | | |
| --- | --- | --- | --- |
| **Id** | **Type of Risk Score** | **Equation for Risk Score** | **Dichotomous decision rule** |
| Ra | The proportion of records in the anonymised dataset that have a re-identification probability higher than a priori predetermined threshold. | $R_{a}=\frac{1}{n}(\sum_{j\in J} f_{j} \times I(\theta_{j}>\tau))$ | $D_{a}=\left\{ \begin{aligned} HIGH, R_{a}>\alpha\\ LOW, R_{a}\leq\alpha\end{aligned} \right.$ |
| Rb | The maximum probability of re-identification among all records in the anonymised dataset. | $R_{b}=\max_{j\in J} (\theta_{j})$ | $D_{b}=\left\{ \begin{aligned} HIGH, R_{b}>\tau\\ LOW, R_{b}\leq\tau\end{aligned} \right.$ |
| Rc | The proportion of records in the anonymised dataset that could be correctly re-identified on average. | $R_{c}= \frac{1}{n} (\sum_{j\in J} f_{j}\theta_{j})$ | $D_{c}=\left\{ \begin{aligned} HIGH, R_{c}>\text{λ} \\ LOW, R_{c}\leq\lambda\end{aligned} \right.$ |
| Where τ = the highest allowable probability of correctly re-identifying a single record  α= the proportion of records that have a high probability of re-identification that would be acceptable to the data custodian  λ = the average proportion of records that can be correctly re-identified that would be acceptable to the data custodian  *I*(.)= the indicator function (this returns 1 if the parameter is true and 0 otherwise)  $f_{j}$ = the number of individuals in an equivalence class j in the anonymised dataset  $J$= the set of equivalence classes in the anonymised dataset  $\theta_{j}$= the probability of re-identification of an equivalence class $j$ (all of the records in the same equivalence class will have the same probability value)  $n$= the total number of records in the anonymised dataset | | | |

The equations in table S4.1.1 need to be adjusted to take into consideration if we are under prosecutor or journalist re-identification risk scenarios. Table S4.1.2 shows how the equations in table S4.1.1 are adapted to the mentioned re-identification scenarios.

| Table S4.1.2 Derived Metrics by Risk Scenario | | |
| --- | --- | --- |
| **Id** | **Type Scenario** | **Equation for Risk** |
| p_Ra | Prosecutor | ${p\_R}_{a}=\frac{1}{n}(\sum_{j\in J} f_{j} \times I(\frac{1}{f_{j}}>\tau)$ |
| p_Rb |  | ${p\_R}_{b}=\frac{1}{\min_{j\in J} (f_{j})}=\max_{j\in J} \left( \frac{1}{f_{j}} \right)$ |
| p_Rc |  | ${p\_R}_{c}=\frac{1}{n}(\sum_{j\in J} f_{j} \times\frac{1}{f_{j}})= \frac{\left\vert J \right\vert}{n}$ |
| j_Ra | Journalist* | ${j\_R}_{a}=\frac{1}{n}(\sum_{j\in J} f_{j} \times I(\frac{1}{F_{j}}>\tau)$ |
| j_Rb |  | ${j\_R}_{b}=\frac{1}{\min_{j\in J} (F_{j})}=\max_{j\in J} \left( \frac{1}{F_{j}} \right)$ |
| j_Rc |  | $j\_R_{c}= \max_{} \left( \frac{\left\vert J \right\vert}{\sum_{j\in J} F_{j}} , \frac{1}{n}\sum_{j\in J} \frac{f_{j}}{Fj} \right)$ |
| Where τ = the highest allowable probability of correctly re-identifying a single record.  $f_{j}$ = the number of individuals in an equivalence class j in the anonymised dataset.  $J$= the set of equivalence classes in the anonymised dataset.  $\left\vert J \right\vert$= the number of unique equivalence classes in the anonymised dataset.  *I*(.)= the indicator function (this returns 1 if the parameter is true and 0 otherwise).  $F_{j}$ = the number of individuals in an equivalence class j in the matching dataset  $n$= the total number of records in the anonymised dataset.  $N=$ the total number of records in the matching dataset. | | |
| *These metrics are suitable for the situation where the anonymised dataset is a proper subset of a theoretical or actual matching dataset. | | |

- **Calculated example**

This example shows how the calculations were executed on the datasets that were made available through our proposed protocol. Table S4.2 displays a mock de-identified dataset with 25 observations and two independent indirect identifiers: gender_coded and age_group. The variables gender, year_of_birth and age are not expected to be in actual anonymised datasets if gender_coded and age_group are present, but are shown here for completeness.

| Table S4.2. Mock Anonymised/De-identified dataset (25 observations, 11 unique classes) | |
| --- | --- |
|  |  |

Prosecutor risk calculation (table S4.3.1) – We obtained three measures of risk under prosecutor risk for the mock anonymised/de-identified dataset presented in table S4.2.

| Table S4.3.1 | |
| --- | --- |
| Classes in De-identified dataset and interim calculations for Risk Scores | Prosecutor Risk Scores Calculation* |
|  |  |
| *Note that for R1a, the tau (0.33) is set a priory | |

Journalist risk (table S4.3.2) – We assumed that a matching dataset exists (with only 340 observations in order to keep the calculations for this example simple; in reality, matching datasets, when they exist, are much larger) for deterministic matching. We obtained three measures of risk under journalist risk for the mock anonymised/de-identified dataset presented in table S4.2.

| Table S4.3.2 | |
| --- | --- |
| Classes in Anonymised/De-identified dataset and interim calculations for Risk Scores | Journalist Risk Scores Calculations* |
|  |  |
| *Note that for R2a, a response curve will be generated, as several values of tau will be explored | |

References

El Emam, K. (2013). Chapter 16 - Measuring the Probability of Re-Identification. Guide to the de-identification of personal health information, CRC Press.
