# Supplementary material for: Evaluating re-identification risks scores in publicly available clinical trial datasets: Insights and implications
Source: Clin Trials. 2025 Aug 22;22(6):649–66. doi: 10.1177/17407745251356423 (PMC12647387; doi:10.1177/17407745251356423)
Supplement: sj-docx-6-ctj-10.1177_17407745251356423 – Supplemental material for Evaluating re-identification risks scores in publicly available clinical trial datasets: Insights and implications [file sj-docx-6-ctj-10.1177_17407745251356423.docx]

Appendix 3 Variables in datasets’ metadata

Metadata items from eligible datasets included:

• Study name/acronym

• Study description

• Study design

• Study objectives

• Study patient population/ Therapeutic area

• Study time frames (e.g., trial duration, follow-up duration)

• Study number of participants/patients (sample size)

• Type of access (Open vs Controlled)

• Clinical phase (i.e., I to IV)

• Number and location of sites

• Date of associated main publication

• Country of lead author in main publication

• Primary outcome statistically significant (Yes/No)

• Nature of active Intervention (e.g., investigational medicinal product, surgery or therapy, route of administration)

• Nature of control intervention (e.g., placebo, routine care)

• Funding source

• Sponsor

• Documentation availability (e.g., protocol, analysis plan, results, data dictionary)
