# Supplementary material for: Evaluating re-identification risks scores in publicly available clinical trial datasets: Insights and implications
Source: Clin Trials. 2025 Aug 22;22(6):649–66. doi: 10.1177/17407745251356423 (PMC12647387; doi:10.1177/17407745251356423)
Supplement: sj-docx-7-ctj-10.1177_17407745251356423 – Supplemental material for Evaluating re-identification risks scores in publicly available clinical trial datasets: Insights and implications [file sj-docx-7-ctj-10.1177_17407745251356423.docx]

Appendix 2 Dataset repositories

Repositories/data sources from 1-15 were identified during “Current recommendations/practices for anonymising data from clinical trials in order to make it available for sharing: A scoping review” ^1^, repositories from 16-18 were found through alternative sources (web searches or word of mouth)

| *Id* | *Data sharing repository* | *Country* | *Funding* | *Type of Access* | *Inclusion* |
| --- | --- | --- | --- | --- | --- |
| *1* | <https://datacompass.lshtm.ac.uk>^2^ | *UK* | *public* | *Controlled* | *Yes* |
| *2* | <https://ctu-app.lshtm.ac.uk/freebird>^3^ | *UK* | *public* | *Open* | *Yes* |
| *3* | <https://datashare.is.ed.ac.uk>^4^ | *UK* | *public* | *Controlled* | *Yes* |
| *4* | <https://www.clinicalstudydatarequest.com>^5^ | *UK* | *public and private* | *Controlled* | *Yes* |
| *5* | <http://datadryad.org>^6^ | *USA* | *public* | *Open* | *Yes* |
| *6* | <http://yoda.yale.edu>^7^ | *USA* | *public and private* | *Controlled* | *Yes* |
| *7* | <https://www.projectdatasphere.org>^8^ | *USA* | *public and private* | *Controlled* | *Yes* |
| *8* | <https://biolincc.nhlbi.nih.gov/studies>^9^ | *USA* | *public* | *Controlled* | *Yes* |
| *9* | <https://nda.nih.gov/get/access-data.html>^10^ | *USA* | *public* | *Controlled* | *Yes* |
| *10* | <https://vivli.org/>^11^ | *USA* | *public and private* | *Controlled* | *Yes* |
| *11* | <https://beta.ukdataservice.ac.uk/datacatalogue/studie>s  (reshare.ukdataservice.ac.uk)  (<https://www.ukdataservice.ac.uk/deposit-data>) ^12^ | *UK* | *public and private* | *Hybrid (1)* | *Yes* |
| *12* | <https://med.data.edu.au/find-data/>^13^ | *Australia* | *public* | *Controlled* | *No (2)* |
| *13* | <https://dcri.org/our-approach/data-sharing/soar-data>  SOAR data: Available datasets: Duke cardiac catheterization datasets. ^14^ | *USA* | *public* | *Controlled* | *No (3)* |
| *14* | https://journals.plos.org/plosone/search ^15^ | *USA* | *public and private* | *Hybrid (1)* | *Yes* |
| *15* | https://www.bmj.com/search/advanced ^16^ | *UK* | *private* | *Hybrid (1)* | *Yes* |
| *16* | [https://dataverse.harvard.edu/](https://dataverse.harvard.edu/%20) ^17^ | *USA* | *public* | *Open* | *Yes* |
| *17* | [https://arlg.org/studies-in-progress/](https://arlg.org/studies-in-progress/%20) ^18^ | *USA* | *private* | *NA* | *No (3)* |
| *18* | https://repository.niddk.nih.gov/studies/ ^19^ | *USA* | *public* | *Hybrid (1)* | *No (3)* |

*(1) Some items are under controlled other seems to be open access*

*(2) Excluded as the repository does not longer exists*

*(3) Excluded because suitable Randomised Controlled Trials datasets could not be located at the moment of search.*

**Reference List**

1. Rodriguez A, Tuck C, Dozier MF, et al. Current recommendations/practices for anonymising data from clinical trials in order to make it available for sharing: A scoping review. *Clinical Trials* 2022; 19: 452-463.

2. London School of Hygiene & Tropical Medicine. LSHTM Data Compass, <https://datacompass.lshtm.ac.uk> (2020).

3. Clinical Trials Unit London School of Hygiene & Tropical Medicine. The FreeBIRD Bank of Injury and Emergency Research Data, <https://freebird.lshtm.ac.uk/> (2020).

4. The University of Edinburgh. Edinburgh DataShare, <https://datashare.ed.ac.uk/> (2020).

5. Clinical Study Data Request (CSDR). Clinical Study Data Request, <https://clinicalstudydatarequest.com/> (2020, 2020).

6. Dryad. Data Dryad, <https://datadryad.org/> (accessed 2020).

7. The Yale University. Yale University Open Data Access (YODA) Project, <http://yoda.yale.edu/> (2020, 2020).

8. CEO Roundtable on Cancer Inc. Project Data Sphere, <https://www.projectdatasphere.org/> (2020).

9. The National Heart LaBIN. BioLINCC, <https://biolincc.nhlbi.nih.gov/> (2020).

10. The National Institute of Mental Health. The NIMH Data Archive (NDA), <https://nda.nih.gov/> (2020).

11. Vivli Center for Global Clinical Research Data. Vivli, a global data-sharing and analytics platform. , <https://vivli.org/> (2020, 2020).

12. UK Data Service. UK Data Service: data Catalogue, <https://beta.ukdataservice.ac.uk/datacatalogue/studies> (accessed 2020).

13. Intersect Australia Limited - Queensland Cyber Infrastructure Foundation Ltd. Australian National Medical Research Data Storage Facility, <https://med.data.edu.au/find-data/> (accessed 2020).

14. Institute DCR. SOAR DATA™, <https://dcri.org/our-approach/data-sharing/soar-data> (2020).

15. PLOS is a nonprofit 501(c)(3) corporation. PLOS ONE: An inclusive journal community working together to advance science by making all rigorous research accessible without barriers, <https://journals.plos.org/plosone/search>.

16. BMJ Publishing Group Ltd. BMJ is a global healthcare knowledge provider with a vision for a healthier world. We share knowledge and expertise to improve healthcare outcomes., <https://www.bmj.com/search/advanced>.

17. Harvard University. Harvard Dataverse Repository. Deposit and share your data. Get academic credit. Harvard Dataverse is a repository for research data. Deposit data and code here., <https://dataverse.harvard.edu/>.

18. Antibacterial Resistance Leadership Group (ARLG) ARLG studies, <https://arlg.org/summary-of-results/>.

19. National Institute of Diabetes and Digestive and Kidney Diseases (NIDDK). NIDDK Central Repository, <https://repository.niddk.nih.gov/studies/dpp/>.
